# Terms of specialized nursing language in the care of the newborn with central venous catheter

**DOI:** 10.1590/0034-7167-2021-0572

**Published:** 2022-07-29

**Authors:** Nanete Caroline da Costa Prado, Harlon França de Menezes, Paulino Artur Ferreira Sousa, Donatila Cristina Lima Lopes, Fernanda Rafaela dos Santos, Rebecca Stefany da Costa Santos, Romanniny Hévillyn Silva Costa Almino, Richardson Augusto Rosendo da Silva

**Affiliations:** I Universidade Federal do Rio Grande do Norte. Natal, Rio Grande do Norte, Brazil; II Universidade Federal Fluminense. Niterói, Rio de Janeiro, Brazil; III Escola Superior de Enfermagem do Porto. Porto, Portugal

**Keywords:** Nursing, Standardized Nursing Terminology, Catheterization, Peripheral, Infant, Newborn, Child Health, Enfermería, Terminología Normalizada de Enfermería, Cateterismo Periférico, Recién Nacido, Salud del Niño, Enfermagem, Terminologia Padronizada em Enfermagem, Cateterismo Periférico, Recém-Nascido, Saúde da Criança

## Abstract

**Objective:**

To construct and validate a specialized nursing terminology for the care of newborns with peripherally inserted central venous catheters (PICC), based on the Betty Neuman Systems Model.

**Methods:**

Methodological study, carried out in a public maternity hospital, operationalized by the steps: extraction of terms from medical records of neonates using PICC; normalization; cross-mapping with the 2019/2020 version of ICNP^®^; organization in the Seven Axes; and content validation with nurses using content validity index and kappa coefficient.

**Results:**

1,718 terms were extracted, and 372 relevant terms were normalized, with 265 constants and 107 non-constants. A total of 335 terms were validated, 246 of which were constant and 89 were not constant, which reached an agreement index and kappa ≥ 0.80.

**Conclusion:**

Relevant terms were identified, which aid newborns using central venous catheters; thus, a terminological subset will be contributed to information in nursing practice.

## RESUMO

**Objetivo:**

Construir e validar uma terminologia especializada de enfermagem para o cuidado a recém-nascidos com cateter venoso central de inserção periférica (PICC), com base no Modelo de Sistemas de Betty Neuman. Métodos: Estudo metodológico, realizado em uma maternidade pública, operacionalizado pelas etapas: extração dos termos de prontuários de neonatos em uso de PICC; normalização; mapeamento cruzado com a versão 2019/2020 da CIPE^®^; organização nos Sete Eixos; e validação de conteúdo com enfermeiros usando-se índice de validade de conteúdo e coeficiente *kappa*.

**Resultados:**

Extraíram-se 1.718 termos, sendo normalizados 372 termos pertinentes, estando 265 constantes e 107 não constantes. Foram validados 335 termos, sendo 246 constantes e 89 não constantes, os quais atingiram um índice de concordância e *kappa* ≥ 0,80.

**Conclusão:**

Identificaramse termos relevantes, que oportunizam a assistência aos recém-nascidos em uso de cateter venoso central; assim se contribuirá com um subconjunto terminológico para a informação na prática de enfermagem.

## RESUMEN

**Objetivo:**

Construir y validar terminología especializada de enfermería para el cuidado a neonatos con catéter central de inserción periférica (PICC), basado en el Modelo de Sistemas de Betty Neuman. Métodos: Estudio metodológico, realizado en una maternidad pública, instrumentalizado por las etapas: extracción de términos de prontuarios de neonatos en uso de PICC; normalización; mapeo cruzado con la versión 2019/2020 de la CIPE^®^; organización en los Siete Ejes; y validez de contenido con enfermeros usándose índice de validez de contenido y coeficiente *kappa*.

**Resultados:**

Extraídos 1.718 términos, siendo normalizados 372 términos pertinentes, estando 265 constantes y 107 no constantes. Fueron validados 335 términos, siendo 246 constantes y 89 no constantes, cuales alcanzaron un índice de concordancia y *kappa* ≥ 0,80.

**Conclusión:**

Identificados términos relevantes, que propician la asistencia a neonatos en uso de catéter venoso central; así se contribuirá con un subconjunto terminológico para la información en la práctica de enfermería.

## INTRODUCTION

The peripherally inserted central venous catheter (PICC) has become an outstanding tool in the care of critically ill patients, as it provides long-term intravenous access and allows different advantages, such as: ease of insertion, short time procedure and few complications, being considered safe and reliable^(1)^.

The role of nurses in the context of care for patients using PICCs has changed and become complex due to the high demand for education, skills and new technologies related to care and its management^(2)^. In this context, nurses need to develop comprehensive and systematized care for patients using this type of catheter, to prevent complications and promote PICC success, given the numerous stressful demands during intravenous therapy.

The Systems Model proposed by Nurse Betty Neuman encompasses the perspectives of factors, whether environmental, emotional, or everyday, that can lead to anxiety and stress. The dynamic balance of human beings is vital through prevention as an intervention designed by the nurse, accompanying the person as a whole, that is, attending within their context of life, in order to maintain a good level of well-being^(3)^.

In this way, nurses need to add a theoretical framework that focuses on their specific practice and base their technical and intellectual skills, to identify and document the care provided through standardized terminology and linked to essential questions of professional practice. In this sense, the International Classification for Nursing Practice (ICNP^®^) is composed of a standardized terminology of nursing language, structured with terms and definitions that allow the systematic collection, description, and documentation of the phenomena of nursing practice^(4)^.

The use of ICNP^®^ in health care provides critical thinking and clinical decision-making, as well as contributing to the recording of nurses’ actions and allowing communication between nursing professionals and other areas^(5)^. It is noteworthy that the record of this care consists of a valuable work instrument, which, in addition to guiding measures for nursing conduct, enables the evaluation of the evolution of assisted users.

Concerning the scientific production on specialized nursing terminology, the literature points to a trend towards studies focused on the care of cancer patients, the elderly and children/adolescents; followed by people living with HIV/AIDS, patients with congestive heart failure and patients in a surgical clinic^(6)^. There is, therefore, a gap in the literature about terminologies specialized in the care of newborns using PICCs.

Given the above, the development of this study is justified by its innovative potential for the construction of a specialized language terminology to assist the NB in the use of PICC. This can support the construction of nursing diagnoses, results, and interventions to be inserted into information systems, facilitating clinical decision-making, and impacting patient quality and safety. In addition, its relevance is highlighted when seeking to advance knowledge about ICNP^®^, strengthening nursing as a science.

The study started from the assumption that it is possible to identify useful terms from the medical records and usable by nurses in the care of newborns using central venous catheters. Thus, the following question arose: Which terms recorded in medical records by nurses can compose a specialized nursing terminology that contributes to the care of NB using PICC?

## OBJECTIVE

To build and validate a specialized nursing terminology for the care of newborns with peripherally inserted central venous catheters (PICC), based on the Betty Neuman Systems Model.

## METHODS

### Ethical aspects

This study was approved by the Research Ethics Committee on August 10, 2020, and the guidelines and regulatory standards for research involving human beings were observed.

### Study design, period, and location

Methodological study, carried out between September 2020 and April 2021, with a high-risk public maternity hospital located in a large city in Northeast Brazil as the setting. The steps recommended for the elaboration of the specialized terminology were followed: 1) process of extracting the terms from medical records; 2) standardization of terms; 3) cross mapping between extracted terms and the terms contained in the ICNP^®^, version 2019/2020, according to the ICNP^®^ Seven Axis Model; 4) content validation, with specialist nurses, of non-constant terms.

### Population and eligibility criteria

In order to determine the number of medical records for the first stage of the study, we used the sample calculation for finite populations with a sampling error of 10%, a confidence level of 95% (Z∞ = 1.96) and a prevalence of 50 %, thus constituting a sample of 65 medical records^(7)^. However, to avoid selection bias, the systematized sampling process was used, with one chart being selected for every three sequentially listed. The interval every three was determined by dividing the total number of patients hospitalized in 2019 (198) by the sample of medical records (65) needed for the study.

The following inclusion criteria were adopted: medical records with nurses’ records during the hospitalization period of newborns using the PICC. The exclusion criteria were: catheter insertion in another institution and medical records with no record in at least one shift of hospitalization, indicating interruption of records.

To calculate the number of specialists^(8)^, the formula was used: n = Zα2 * P* (1 - P)/e^2^, where “Zα” refers to the adopted confidence level (95%); “P” represents the proportion of experts who indicate the adequacy of the items (85%); and “e” represents the acceptable proportional difference from what can be expected (15%). Therefore, as the coefficient “Zα” according to the normal distribution pattern assumes a tabulated value of 1.96 for a 95% confidence level, the final sample size calculation was defined by: n = 1.962*0, 85*0.15/0.152 = 22 specialists.

To identify the specialists, the criteria of a study^(9)^ were used in an adapted way. Thus, nurses called specialists from a research group on ICNP^®^ studies from a federal university in Brazil participated in the research, needing to present at least two of the following criteria: nurse with a master’s or doctorate degree; researchers in the ICNP^®^ area; assistance nurses in the area of neonatology; and have published an article on terminology or Nursing Process using the ICNP^®^.

After refining the established criteria, they were invited to participate in the research through an invitation letter, via e-mail, with the forwarding of the Free and Informed Consent Form and an electronic form, via Google Forms, composed of the terms, definitions, and analysis. The evaluation took place in two rounds. The 22 specialists who participated in the study were mostly female (75%), between 35 and 55 years of age (65%), had a master’s degree in Nursing (65%) and worked in the hospital area (90%).

### Study protocol

For the first stage, records made by nurses were selected from the physical records of patients hospitalized in the year 2019 at that hospital. In the established period, 198 neonates who used the catheter were hospitalized at the institution, according to data obtained by the Medical Archives Service.

Data collection was performed by the main researcher, being supervised by the study advisor. In order to extract the terms, a Microsoft Office Excel^®^ 2016 spreadsheet was created, consisting of two columns for each medical record: transcription of the paragraphs of the records and terms extracted from that paragraph. The content was grouped into a file in Word^®^ format, which was converted to Portable Document Format - PDF. Subsequently, the terms were extracted using a tool called PorOnto, which processes information by using ontologies on a large scale, being widely used in the health area, since it has a complexity of its knowledge^(10)^. This generated a list of terms organized in order of frequency, arranged in an Excel spreadsheet^®^.

After excluding repetitions, the terms were organized in alphabetical order. Only the first time the term appeared was considered, and the number of repetitions was counted; finally, the terms found were normalized. As a result, spelling correction, standardization of verb tenses, grammatical genres and number, adequacy of acronyms and exclusion of pseudo-terminological expressions were carried out.

Subsequently, cross-mapping was carried out to identify constant and non-constant terms in the ICNP^®^ 2019/2020 version, which made it possible to compare and identify similarities, in addition to promoting the necessary adaptations to the standardized terminology. This step was based on the ISO 12300:2016^(11)^ Standard, which discusses the mapping process and its scope to enable the creation of clinical terminologies or subsets for specific use. Thus, the terms extracted from the medical records were crossed with those from the ICNP^®^, through Microsoft Office Access^®^ 2016.

With the mapping completed, operational definitions were created for all non-constant terms, using ICNP^®^, scientific articles and dictionaries in Portuguese and technical health terms, in order to correspond to the validation stage by specialists. The construction of these definitions occurred as follows^(12)^: 1) development of a preliminary definition; 2) literature review; 3) development or identification of specific characteristics; 4) mapping the meaning of the concept; and 5) affirmation of the operational definition.

It is noteworthy that, for terms not included in the ICNP^®^, the degree of equivalence was determined, as proposed in ISO/TR 12300:2016^(11)^, which establishes a scale for assessing meanings: 1 - Equivalence of lexical meaning and also conceptual; 2 - Equivalence of meaning, but with synonymy; 3 - The source concept is broader and has less specific meaning than the target concept/term; 4 - The source concept is more restricted and has more specific meaning than the target concept/term; 5 - No mapping is possible.

The last step was the content validation of the terms identified in the study. Therefore, the group of specialist nurses could indicate whether they considered them applicable or not, as well as insert suggestions. The adjustments/adjustments made were re-discussed with the specialists.

### Data analysis and processing

The terms were distributed according to the Action, Customer, Focus, Judgment, Location, Means and Time axes, considering agreement on the meaning of the term and the definitions of each axis. With this, a form was organized composed of the terms, their allocations in the ICNP^®^ axes and definitions, in line with Betty Neuman’s theoretical framework, in the three levels of stressors - intrapersonal, interpersonal and extra-personal - in order to support the study.

For data analysis, the Content Validity Index (CVI) was adopted. The indices were calculated for the scores assigned to the terms, based on a five-point Likert scale (1 - not at all relevant; 2 - little relevant; 3 - relevant; 4 - very relevant; 5 - very relevant), with the terms being validated with CVI ≥ 0.80. The CVI was calculated based on dividing the number of judges who judged the item as adequate by the total number of experts. For the global assessment of terms, the calculation was performed through the ratio between the number of items considered adequate by the experts and the total number of items. The reliability of the agreement between the assessment of the items in the evaluation of the judges was also analyzed, using the kappa coefficient (k), indicated as a complement to the CVI. As an acceptance criterion, an agreement > 0.80 was established for the CVI and kappa coefficient, both for the evaluation of each item and for the general evaluation of the terms^(13-14)^.

## RESULTS

A total of 1,718 terms were extracted from the nurses’ records from the medical records of neonates with the PICC catheter, which, after the normalization process, resulted in 372 useful terms. Afterwards, these terms were cross-mapped, accounting for 265 constant terms and 107 not constant terms in the ICNP^®^ 335 were validated, 246 of which were constant (Chart 1) and 87 were not constant (CVI > 0.80/k > 0.80) (Chart 2). As for the constant terms, those related to the Focus (33.7%) and Action (31.3%) axes prevailed, followed respectively by the Middle axes (9.3%), Location (8.1%), Judgment (7, 3%), Time (6.9%) and Client (3.2%). The frequencies of appearance (f) of the most relevant terms are presented throughout the text.

**Chart 1:** Terms contained in the CIPE^®^ 2019/2020, Natal, Rio Grande do Norte, Brazil, 2021

Axis	n^*^	Constant terms and CIPE codes^®^
Focus	(n = 83)	Access (10000340); Intravenous (or Intravenous) Access (10010780); Adaptation (10001741), Agitation (10002035); Allergy (10041119); Apnea (10035012); Family Support (10023680), Social Support (10024074), Arrhythmia (10002536); Attention (10002924), Psychomotor Activity (10016008); Bradycardia (10003613); Cry (10005415); Comfort (10004655); Complication (10025459); Complication Associated with Health Care (10041277); Length (10011312); Communication (10004705); Condition (10018793); Cardiovascular Condition (10033946); Tissue Perfusion Condition (10019750); Nutritional Condition (10013419); Control (10005135), Pain Control (10005157); Growth (10008563); Caring (or Taking Care) (10004002); Discomfort (10023835); Human Development (10009200), Electrolyte Imbalance (10031186); Liquid Imbalance (10031309); Dehydration (10041876); Dyspnea (10006461); Pain (10013950); Edema (10041951); Peripheral Edema (10027476); Liquid Balance (or Water Balance) (10034114); Stress (10018888), Heart Rate (10008833); Respiratory Rate (10016904); Hematoma (10008931); Hydration, Adequate (10042342); Hyperglycemia (10027521), Hypoglycemia (10027513); Hypothermia (10009547); Disability (or Limitation) (10005980), Infection (10010104); Food Intake (10006517); Skin integrity (10018241); Tissue Integrity (10003530); Drug Interaction, Adverse (10042716); Hand Hygiene (10041190); Hypothermia (10009547); Integrity (10010416); Injury (10010284), Need (10012495); Need for Care (10030878); Obstruction (10013555); Weight (10021034), Blood Pressure (10003335); Procedure (10034409); Immune System Process, Impaired (10041093); Vascular Process (10020620); Scheme (10016609); Parenteral Nutrition Regimen (10032215); Medication Regimen (10011884); Response to Thermoregulation (10017062); Response to Treatment (10017070); Laboratory Result (10011074); Routine (10017384); Bleeding (10003303); Health (10008711); Service (10017908); Vital Sign (10020829); Symptom (10019368); Cardiovascular System (10003936); Body System (10003480); Sleep (10041399); Infection Susceptibility (10019306); Tachycardia (10019415); Rate (10016390); Body Temperature (10003507); Thermoregulation (10019644); Link (10003548).
Judgment	(n = 18)	High (10009007); Late (or Slow) (10022089); Low (10011438); Dependency (10026671); Effective (10014956); Large (10011116); Light (10025854); Enhanced (10026692); Moderate (10025865); Negative (10010981); Small (10018315); Positive (10010981); Potential for Risk (10017252); Impaired (10012938), Real (10000420) Risk (10015007), Severe (10025877); Simple (10024061).
Client	(n = 8)	Community (10004733); Child (10004266); Family (10007554); Group (10008544); Individual (10010018); Family Member (10007596); Patient (10014132); Newborn (10013187).
Action	(n = 77)	Action (10000386); Follow (10042609); Administer (10001773); Adjust (10001760); Relieve (10002171); Change (10002185); Analyze (10002298); Apply (10002464); Arrange (10002527); Answer (10002911); Attend through intervention (10034612); Attenuate (10016716); Increase (10009961); Auscultate (10003012); Auxiliary (10002850); Evaluate (10007066); Cover (10005296); Put or put (10016201); Compress (10004877); Check (10004189); Confirm (or Verify) (10020727); Count (10005265); Contain (or Limit) (10017155); Describe (10005797); Determine (10005824); Determine Intervention (10034620); Decrease (10005600); Document (10006173); Establish (10024813); Sterilize (10018826); Stimulate (10018842); Avoid (10003077); Examine (10007256); Execute (10014291); Run Surveillance (10019277); Rubbing (10017397); Guarantee (or Assure) (10006950); Manage (10011625); Sanitize (or Take Care of Hygiene) (10009285); Identify (10009631); Implement (10009840); Start (10010221); Inspect (10010348); Install (10010353); Clear (10004444); Manipulate (10011710); Keep (10011504); Maintain Intravenous (or Intravenous) Access (10036577); Measure (or verify) (10011813); Monitor (10012154); Notify (10001917); Observe (10013474); Get(10013572); Get Data (10002673); Arrange (10013806); Palpate (10013997); Position (10014757); Prepare (10015478); Prescribe (10015510); Prevent (10015620); Prevent Contamination (10005055); Promote (10015801); Protect (10015864); Provide (10015935); Puncture (10016152); Reinforce (10016650); Register (10016498); Remove (10016763); Require (or Require) (10016873); Restore (10017140); Restrict (or Contain) (10017172); Supervise (10019093); Suspend Usage (10036651); Treat (10019525); Swap(10004162); Watch (or Investigate) (10019283).
Middle	(n = 23)	Central Venous Access (Central Line) (10004115); Needle (10012509); Food (10008089); Analgesic (10002279); Antibiotic (10002383); Venous Cannula (10020677); Catheter (10004087); Compresses/Gauges (10008378); Security Device (10017425); Invasive Device (10034244); Vascular Access Device (10034484); Cover Device (10005306); Infusion Device (10033352); Device for Monitoring (10012177); Nurse (o) (10013333); Interprofessional Team (10039400); Incubator (10009988); Material (10011775); Medication (10011866); Patient Record (10014178); Protocol (10015926); Aseptic Technique (10002639); Intravenous (or Intravenous) Therapy (10010808).
Location	(n = 20)	Abdomen (10000023); Forearm (10008164); Cardiovascular System Component (10003943); Body System Component (10003498); Immune System Component (10025073); Heart (10008822); Body (10003388); Invasive Device Location (10010854); Neck (10012476); Position (10014788); Lung (10011486); Skin (10018239); Axillary Region (10003096); Body Region (10003451); Umbilical Region (10020259); Intensive Care Unit (10010444); Blood Vessel (10003374); Vein (10020665); Intravenous (or Intravenous) Route (10010798); Parenteral Route (10014047).
Time	(n = 17)	Admission (10001843); High (10006000); Continuous (10005086); Duration (10006379); Event or Episode (10007239); Exam (10007241); Frequency (10008234), Hospitalization (10009122); Gestational Age (10037063); Start (10013689); Intermittent (10010485); Morning (10012226), Evening (10013207); Present (10015581), Week (10021010); Status (10018202); Afternoon (10001955).

* *n - absolute number.*

**Chart 2:** Terms not included in the CIPE^®^ 2019/2020, Natal, Rio Grande do Norte, Brazil, 2021

Axis	n^*^	Constant terms and CIPE codes^®^
Focus	(n = 35)	Infectious Agent; Anemia; Bradypnea; Headboard; Comorbidity; Bat Counting; Leukocyte Count; Platelet Count; Caution; Oxygen Desaturation; Organic Dysfunction; Coagulation Disorder; Diuresis [Absent, Spontaneous, Present, Reduced]; Dosage of Electrolytes; Ecchymosis; General Status [Good, Compromised, Severe, Fair]; Sepsis Stage; Clinical Evolution; Risk factor; Phlebitis; Infectious Focus; blood glucose; hyperemia; Infiltration; Prevention; Mean Blood Pressure; Pulse [Full, Filiform, Thin, Impalpable]; Replacement; Peripheral Oxygen Saturation; sepsis; Signs of Organic Dysfunction; Signs of Tissue Hypoperfusion; Tachypnea; Pulse Pressure Variation; Infusion Flow.
Judgment	(n = 4)	Abundant; Absent; Inaudible; Prematurity.
Client	(n = 4)	Serious Patient; Newborn Patient; neonate; Premature Newborn.
Action	(n = 22)	Approach; To adopt; Reach; Absorb; Suit; Purchase; To help; set; Appear; administer; To understand; Compromise; structure; To focus; flush; Immunize; Clean; provide; Accomplish; reference; Non-Elective Removal; Require; Check.
Middle	(n = 19)	Antiseptic; Biochemistry; Infusion bomb; Peripherally Inserted Central Venous Catheter (PICC); Diet; Vasoactive Drug; Electrocardiogram; Pain Scale; Agitation Scale; Multiprofessional Team; radiography; Laboratory Examination; Microbiological Examination of Culture [Catheter Tip, Blood]; Arterial blood gas analysis; Guideline; Contact Isolation; Medicine; Result of Cultures; Venoclysis.
Location	(n = 1)	Laboratory.
Time	(n = 2)	Early Detection; Time.

* *n - absolute number.*

With regard to non-constant concepts, those related to the Focus (39.3%) and Action (24.7%) axes prevailed, followed respectively by the Medium axes (22.4%), Client (5.6%), Judgment (4.4%), Time (2.2%) and Location (1.1%) (Chart 2).

## DISCUSSION

In general, the PICC offers a circulating route for the rescue and successful treatment of newborns, especially those with very low birth weight and critically ill children, avoiding problems such as infections and pain caused by repeated punctures^(15)^. From the point of view of the catheter insertion process, the terms “Length” (f = 49), “Invasive Device Location” (f = 31) and “Aseptic Technique” (f = 57) were relevant. These terms demonstrate the need for specific technical knowledge for the installation of this type of device and the recognition of these as stressors for the child.

For newborns, especially those with very low birth weight, precise positioning of the PICC tip is extremely important. The occurrence of severe arrhythmia after device insertion, for example, may be associated with an excessively deep position of the catheter. Thus, radiography should be performed immediately to identify the position of the catheter tip^(15)^. A study pointed out that the non-central position of the PICC tip was the only independent risk factor for the non-selective removal of this device^(3)^. Therefore, estimating the insertion length before the procedure to reduce the rate of adjustment afterward is crucial in neonatal care; therefore, it is considerable that the terms “Length” (f = 49) and “Location of Invasive Device” (f = 31) have gained higher frequencies of appearance.

Another frequent term was “Aseptic Technique” (f = 57). PICC insertion is primarily indicated for premature or critically ill babies. These patients are susceptible to nosocomial infections due to immature tissue and organ function and low immune function; while the long-term use of a PICC also makes the incidence of catheter-related bloodstream infection (CRBSI) highly likely.

CRBSI is the most serious complication after PICC puncture^(14-15)^. An observational study^(16)^ revealed that the implementation of a maximum sterile barrier during device insertion in neonates independently contributed to a reduction in the risk of central catheter-associated bloodstream infection. Therefore, it is essential that the nurse in the care of the NB with PICC uses maximum barrier measures in the insertion of the catheter and carefully evaluates if the continuation of the insertion of the device is necessary^(15)^.

Regarding the catheter maintenance process, the constant terms “Monitor” (f = 28), “Evaluate” (f = 34), “Inspect” (f = 13), “Monitor” (f = 43) and “Prevent complications” (f = 55) were frequent in this study. These terms are relevant, possibly, because they provide for interventions that can prevent the occurrence of complications resulting from the use of PICC and contribute to the safety of catheter maintenance. For the nursing team, it is essential to constantly monitor the condition of the device in order for the intravenous therapy to be carried out successfully.

With regard to PICC complications, the terms “Non-elective Removal” (f = 16), “Complication” (f = 67), “Infection” (f = 105), “Obstruction” (f = 73), “Infiltration” (f = 22), “Phlebitis” (f = 45) were also frequent, which demonstrate the need to manage the neonate using the device, which may have an important influence on keeping the catheter patent and free of complications. A study^(17)^ points out that PICC is associated with a reduced incidence of complications, such as catheter occlusion and infiltration, compared to short peripheral catheters. Terms related to non-elective removal and complications associated with the use of PICC are relevant, given that these variables are associated with morbidity, so clinical data can help in efforts to improve the quality of care provided. In addition, identifying modifiable risk factors for complications is especially important so that the team can plan care to prevent catheter-associated complications^(3)^.

From a clinical point of view, the term “Hypothermia” (f = 36) was identified as prevalent. Thermal regulation is one of the critical factors in the survival and stability of the newborn, and the control of the newborn’s body temperature is a challenge, since, in this population, it is common for heat loss to be greater than production. The lower the gestational age and birth weight, the more significant these losses and the greater the risk of hypothermia. This phenomenon is due to the fact that very low birth weight preterm infants have a relatively large surface area compared to their weight, thin skin, dispersed subcutaneous tissue, low glycogen stores and almost no brown fat stores, inadequate skin keratinization and control. inadequate vascular supply for thermoregulation^(18)^.

Given this condition, nurses need to seek scientific knowledge about the physiology of NB thermal control, discuss the importance and identify key points of this control and develop strategies to improve it. Nursing measures to maintain body temperature combined with the assessment of blood vessels are important conditions to improve the success rate of a puncture in neonates^(15)^.

Terms such as “Edema” (f = 59), “Hematoma” (f = 37), “Skin Integrity” (f = 27) are linked to the neonate using the PICC. The most common skin complications in these scenarios are bruising, edema, and changes in skin integrity. Significant risk factors for these complications are related to both the device and poor skin integrity^(19)^.

Terms such as “Oxygen Desaturation” (f = 12) and “Tachycardia” (f = 23) were also identified in the present research. The literature points to the premise that stressful factors such as the performance of invasive procedures, individually or associated with the sensation of pain, can cause the disorganization of different systems, contributing to changes in the physiological parameters of hospitalized neonates^(20)^. During the insertion of intravenous devices, the neonate may experience changes in blood pressure, heart rate (HR), respiratory rate (RR) and peripheral oxygen saturation (SpO_2_). In addition, with the procedure, hormonal indicators of stress may be released, such as cortisol, adrenaline, and noradrenaline, being important the constant monitoring of the nurse during the insertion of the catheter^(21)^.

Terms related to the stress conditions of neonates using PICCs were: “Agitation” (f = 17), “Pain” (f = 33) and “Crying” (f = 21). These are mentioned when mentioning the handling of the NB during the PICC insertion procedure and dressing changes, and are frequently documented in the NICU^(22)^. The painful experience of neonatal results in physiological and behavioral changes and in the development of the nervous system, which can lead to future damages. However, it is necessary to continuously evaluate procedures that cause pain, given that many are necessary as diagnostic and therapeutic support^(23)^.

Regarding the development and growth of the neonate using the PICC, the most frequent terms were “Prematurity” (f = 97), “Parenteral Nutrition Regimen” (f = 19) and “Weight” (f = 09). Prematurity is seen as a health problem in the world, and Brazil is among the ten countries with the highest rates. According to the Ministry of Health, prematurity is the main cause of infant mortality in the first month of life^(24)^. In Brazil, there are about 931 premature births per day, which characterizes a rate of 12.4% of births, corresponding to twice the number of cases in some European countries^(25)^.

As a result, there are multiple considerable nuances of nursing care that need attention in the face of neonatal complexity. In this sense, a study^(26)^ suggests that birth weight is a risk factor for complications associated with PICC, and infectious and non-infectious complications of this device are associated with low body weight gain in premature babies.

Regarding the social context of the NB using PICC, the terms “Multi-professional Team” (f = 12) and “Family” (f = 61) were frequent. In the context of care for the NB using the PICC, this team has a significant role, and its actions must meet the National Policy for Humanized Care for the Newborn, which advocates qualified and humanized care for the newborn and his family^(27)^.

Admission to a neonatal unit is an event that can contribute negatively to the parent-infant interaction, with adverse consequences for the family nucleus. In this scenario, the family experiences feelings of concern and lack of confidence in their own ability to care for their child. Therefore, it is important to provide assistance to all family members, not just the hospitalized child and his mother^(28)^.

It is noteworthy that, for the development of a specialized terminology, it is essential to use a theory or conceptual model, to support and serve as a foundation for nursing practice. There are several theoretical models of nursing care, such as Betty Neuman’s systems model, which was used as a theoretical framework for the present study. Of a predominantly holistic nature^(29)^, the Neuman model is oriented towards the well-being of the patient, from an open system perspective and constant interaction with the environment, aiming to reduce the possible damages of internal and external stressors.

The neonate using the PICC is exposed to different stressors that can harm their safety^(30)^, and it is necessary for nursing to adopt practices that can minimize the risks of damage arising from these stressors. In this sense, in the present study, terms considered representative of stressors can be identified, which are subdivided into intrapersonal, inter-personal and extra-personal stressors. As intrapersonal, “Prematurity” was identified; among interpersonal factors, “Hypothermia”, “Oxygen Desaturation”, “Tachycardia” stood out; as extra-personal, we found “Edema”, “Hematoma”, “Infection”, “Obstruction”, “Infiltration”, “Phlebitis”. In this sense, it is important for nurses to identify stressors, in order to list care measures aimed at stabilizing the patient’s system to achieve well-being.

Finally, as nursing care and the profession have already reached the status of science, this is currently emphasizing the importance of records and awareness of the relevance of documenting nursing practice, under the aspect that information is the essential element to systematize a structured care model. Thus, the fundamental role of specialized terminologies is highlighted, which reveals considerable evolution for the documentation of practice through its methodology in the standardization of language^(31-33)^.

In the context of care for the NB using the PICC, the identification and use of these well-designed and standardized terms favor the unification of professional language, facilitate communication between health professionals, allow the use of terms that are more appropriate to the real health needs, enable the assessment of care and the generation of indicators and promote the safety of the care provided. Therefore, this study represents a contribution to the structuring of nursing diagnoses, results and interventions, thus contributing to the operationalization of the Nursing Process^(34)^.

### Study limitations

A limitation of the study was that it was carried out in only one service, which may not reflect the reality of other institutions that provide care to neonates using peripherally inserted central venous catheters.

### Contributions to the Nursing Area

The construction of a specialized terminology for the care of new-borns using PICCs contributes to: the process of consolidating the nursing language and its body of knowledge in this area; advancement in knowledge about ICNP^®^; and construction and implementation of care instruments, focusing on patient safety and supporting the development of the Nursing Process.

## CONCLUSION

The study allowed the construction and validation of the content of a specialized nursing terminology for the care of newborns using the PICC, with the terms contained in the ICNP^®^ 2019/2020 predominating. However, a significant number of terms were identified that are used in their clinical practice and not included in the terminology, which indicates the need for their expansion in order to involve, in some way, the specific phenomena of this practice. With this, it is suggested that such terms can be sent to the International Council of Nurses, to contribute to the updating of ICNP^®^. In short, potential new studies will enable the development of a terminological subset aimed at the care of this population, as well as the applicability of clinical nursing indicators and care models for this public.

## SUPPLEMENTARY MATERIAL

This manuscript is the result of the dissertation “Terminological subset of the International Classification for Nursing Practice (ICNP^®^) in neonates with peripherally inserted central venous catheters in the light of Betty Neuman’s theory” [Internet]. 2020. Graduate Program in Nursing, Federal University of Rio Grande do Norte. Available in: https://repositorio.ufrn.br/handle/123456789/31723

0034-7167-reben-75-06-e20210572-sup01
