# Supplementary material for: Terms of specialized nursing language in the care of the newborn with central venous catheter
Source: Rev Bras Enferm. 2022 Jul 29;75(6):e20210572. doi: 10.1590/0034-7167-2021-0572 (PMC9746261; doi:10.1590/0034-7167-2021-0572)
Supplement: 0034-7167-reben-75-06-e20210572-sup01 [file 0034-7167-reben-75-06-e20210572-sup01.pdf]

UNIVERSIDADE FEDERAL DO RIO GRANDE DO NORTE
CENTRO DE CIÊNCIAS DA SAÚDE
DEPARTAMENTO DE ENFERMAGEM
PROGRAMA DE PÓS-GRADUAÇÃO EM ENFERMAGEM
CURSO DE MESTRADO ACADÊMICO

NANETE CAROLINE DA COSTA PRADO

SUBCONJUNTO TERMINOLÓGICO DA CLASSIFICAÇÃO INTERNACIONAL PARA A PRÁTICA DE ENFERMAGEM (CIPE®) EM NEONATOS COM CATETER VENOSO CENTRAL DE INSERÇÃO PERIFÉRICA À LUZ DA TEORIA DE BETTY NEUMAN

NATAL
2020

NANETE CAROLINE DA COSTA PRADO

SUBCONJUNTO TERMINOLÓGICO DA CLASSIFICAÇÃO INTERNACIONAL PARA A PRÁTICA DE ENFERMAGEM (CIPE®) EM NEONATOS COM CATETER VENOSO CENTRAL DE INSERÇÃO PERIFÉRICA À LUZ DA TEORIA DE BETTY NEUMAN

Dissertação apresentada ao Programa de Pós Graduação em Enfermagem, nível Mestrado, do Centro de Ciências da Saúde, da Universidade Federal do Rio Grande do Norte – UFRN, na área de Enfermagem na Atenção à Saúde, inserido na linha de pesquisa Enfermagem na vigilância à saúde, como requisito para obtenção do título de Mestre em enfermagem.

Área de Concentração: Enfermagem na atenção à saúde.

Linha de Pesquisa: Enfermagem na vigilância à saúde.

Orientador: Prof. Dr. Richardson Augusto Rosendo da Silva.

NATAL

2020

Universidade Federal do Rio Grande do Norte - UFRN
Sistema de Bibliotecas - SISBI
Catalogação de Publicação na Fonte. UFRN - Biblioteca Central Zila Mamede

Prado, Nanete Caroline da Costa.
Subconjunto terminológico da classificação internacional para a prática de enfermagem (CIPE®) em neonatos com cateter venoso central de inserção periférica à luz da teoria de Betty Neuman / Nanete Caroline da Costa Prado. - 2020.
103 f.: il.

Dissertação (mestrado) - Universidade Federal do Rio Grande do Norte, Centro de Ciências da Saúde, Programa de Pós-Graduação em Enfermagem, Natal, RN, 2021.
Orientador: Prof. Dr. Richardson Augusto Rosendo da Silva.

1. Recém-nascido - Dissertação. 2. Diagnóstico de enfermagem - Dissertação. 3. Cateterismo venoso central - Dissertação. I. Silva, Richardson Augusto Rosendo da. II. Título.

RN/UF/BCZM CDU 616-083-071

Elaborado por Ana Cristina Cavalcanti Tinôco - CRB-15/262

SUBCONJUNTO TERMINOLÓGICO DA CLASSIFICAÇÃO INTERNACIONAL PARA A PRÁTICA DE ENFERMAGEM (CIPE®) EM NEONATOS COM CATETER VENOSO CENTRAL DE INSERÇÃO PERIFÉRICA À LUZ DA TEORIA DE BETTY NEUMAN

Dissertação apresentada ao Programa de Pós Graduação em Enfermagem do Departamento de Enfermagem da Universidade Federal do Rio Grande do Norte – UFRN, na área de Enfermagem na Atenção à Saúde, inserido na linha de pesquisa Enfermagem na vigilância à saúde, para obtenção do título de Mestre em enfermagem.

Local e Data: Natal/RN, 15 de dezembro de 2020.

Resultado: APROVADA

BANCA EXAMINADORA

____

Prof. Dr. Richardson Augusto Rosendo da Silva

____

Prof. Dr. Paulino Artur Ferreira Sousa

____

Prof. Nuno Damácio de Carvalho Félix

____

Profa. Maria Alzete de Lima

AGRADECIMENTOS

A **Deus**, pela dádiva da vida, pelo cuidado em cada detalhe passo meu, por Seus sonhos e planos melhores que os meus. Como forma de agradecer a vida e por minha chegada até aqui, cito o rei Davi no livro de Salmos capítulo 139 e versículo 16: “Os teus olhos me viram a substância ainda informe, e no teu livro foram escritos todos os meus dias, cada um deles escrito e determinado, quando nem um deles havia ainda”. Do mesmo modo, faço minhas, as palavras do escritor aos Romanos capítulo 11 e versículo 12: “(...) na esperança, sede alegres; na tribulação, pacientes; na oração, perseverantes.”

Ao **meu esposo Eduardo Rodrigues**, meu amor e companheiro nessa jornada chamada vida. Obrigada por ter acredita em meu potencial, por ter sonhado comigo e por me apoiar em meus sonhos e aspirações como mulher, esposa e enfermeira.

Aos **meus pais Walterlins e Neuza**, pela educação, pelo incentivo e por serem meu porto seguro. Obrigada por me tornarem o ser humano que sou. Obrigada por me permitiram sonhar e alcançar o sonho de ser enfermeira.

A **minha irmã Karine**, que compartilhou dos meus sonhos e me incentivou em todos os momentos. Obrigada pelos momentos de alegria e cumplicidade.

A **minha avó Chiquinha (*in memorian*)**, que sempre desejou as bençãos de Deus sobre a minha vida, que me ajudou a ser uma mulher forte e corajosa.

Aos **meus amigos**, que torceram por cada sonho e vibraram a cada conquista. Ao carinho de todos nos momentos de confraternização.

Ao **meu orientador Dr. Richardson Augusto Rosendo da Silva**, por ter me escolhido como orientanda e confiar em meu trabalho. Obrigada pelas oportunidades, por me ajudar a percorrer o caminho da pesquisa científica e por me incentivar cada dia a lutar em busca da valorização da Enfermagem. Juntos, produzimos e iremos produzir frutos de uma jornada de esforço coletivo.

A **Turma de Mestrandos**, pela amizade e aprendizado mútuo.

Ao **Programa de Pós-graduação em Enfermagem-PGENF/UFRN**, pela formação de alta qualidade, pelo foco no desenvolvimento científico e engrandecimento da Enfermagem.

Aos **professores do Programa** pelos saberes compartilhados e por serem uma inspiração na minha jornada acadêmica e profissional.

Aos **pequenos pacientes - bebês participantes do estudo**, e seus responsáveis legais, que me permitiram dar forma e vida a presente pesquisa, na busca pelo cuidado cada vez mais qualificado.

Aos **professores da Banca Examinadora** da dissertação pela disponibilidade, contribuições e feedbacks construtivos.

RESUMO

PRADO, Nanete Caroline da Costa. **SUBCONJUNTO TERMINOLÓGICO DA CLASSIFICAÇÃO INTERNACIONAL PARA A PRÁTICA DE ENFERMAGEM (CIPE®) EM NEONATOS COM CATETER VENOSO CENTRAL DE INSERÇÃO PERIFÉRICA À LUZ DA TEORIA DE BETTY NEUMAN. 2020. 103 fls.** Dissertação (Mestrado em Enfermagem) – Programa de Pós-Graduação em Enfermagem, Universidade Federal do Rio Grande do Norte, Natal/RN, 2020.

O Cateter Venoso Central de Inserção Periférica (CCIP) é destaque entre os cateteres centrais inseridos em unidades de terapia intensiva neonatal. O manejo do cateter PICC pressupõe habilidades e competências que favoreçam a segurança do recém-nascido e que reduzam os riscos de complicações associadas ao cateter. Nesta diretiva, como ferramenta que organiza a prática e direciona as necessidades do paciente, a Sistematização da Assistência de Enfermagem (SAE) possui significativo impacto na qualidade da assistência direcionada ao paciente. Nesse sentido, o estudo teve como objetivo, desenvolver um Subconjunto Terminológico da CIPE® para recém-nascidos com Cateter Venoso Central de Inserção Periférica, tendo como suporte teórico o Modelo de Sistemas desenvolvido por Betty Neuman. Trata-se de um estudo metodológico, com amostra de 124 neonatos, desenvolvido em quatro etapas: 1ª - identificação dos fenômenos da Prática de Enfermagem para neonatos em uso do PICC; 2ª - elaboração dos Diagnósticos, Resultados e Intervenções de Enfermagem; 3ª - Construção dos enunciados diagnósticos, resultados e intervenções; 4ª - validação e análise estatística dos enunciados de Diagnósticos, Resultados e Intervenções de Enfermagem; 5ª - Estrutura do subconjunto terminológico da CIPE. O projeto foi submetido ao Comitê de Ética em Pesquisa da Universidade Federal do Rio Grande do Norte, obtendo-se aprovação com o Certificado de Apresentação para Apreciação Ética (CAAE) 48990515.0.0000.5292. Procedeu-se à análise dos dados através do Índice de Validação de Concordância (IVC > 0.80). Foram validados 31 diagnósticos/resultados e 154 intervenções de enfermagem, as quais compuseram o Subconjunto terminológico, entre eles: Risco de hemorragia, Agitação, Dor, Risco de Infecção, Hipotermia, Termorregulação Prejudicada, Edema, Hematoma, Integridade da Pele Prejudicada e Sinal Vital Alterado. Conclui-se que a partir dos registros de enfermagem e identificação dos achados clínicos foi possível elaborar os diagnósticos, resultados e intervenções de enfermagem da CIPE® para recém-nascido com Cateter Venoso Central de Inserção Periférica. Ademais, a utilização do modelo de sistemas de Neuman representou um alicerce conceitual-filosófico capaz de direcionar a avaliação clínica para a identificação dos estressores e das suas eventuais causas.

Descritores: Processos de enfermagem; Terminologia; Diagnóstico de enfermagem; Enfermagem; Cateterismo Venoso Central; Recém-nascido.

ABSTRACT

PRADO, Nanete Caroline da Costa. TERMINOLOGICAL SUBCONJECT OF INTERNATIONAL CLASSIFICATION FOR NURSING PRACTICE (ICNP®) IN NEONATES WITH CENTRAL VENOUS CATHETER OF PERIPHERAL INSERTION IN THE LIGHT OF THE THEORY OF BETTY NEUMAN. 2020. 103 pages Dissertation (Master in Nursing) - Graduate Program in Nursing, Federal University of Rio Grande do Norte, Natal / RN, 2020.

The Peripherally Inserted Central Venous Catheter (PICC) stands out among the central catheters inserted in neonatal intensive care units. The handling of the PICC catheter presupposes skills and competencies that favor the safety of the newborn and that reduce the risks of complications associated with the catheter. In this directive, as a tool that organizes the practice and directs the patient's needs, the Nursing Care Systematization (SAE) has a significant impact on the quality of care directed to the patient. In this sense, the study aimed to develop a CIPE® Terminological Subset for newborns with peripherally inserted central venous catheters, with theoretical support for the Systems Model developed by Betty Neuman. This is a methodological study, with 124 newborns, developed in four stages: 1st - identification of the Nursing Practice phenomena for newborns using the PICC; 2nd - elaboration of Nursing Diagnostics, Results and Interventions; 3rd - Construction of the diagnostic statements, results and interventions; 4th - validation and statistical analysis of the statements of Diagnostics, Results and Nursing Interventions; 5th - CIPE terminology subset structure. The project was submitted to the Research Ethics Committee of the Federal University of Rio Grande do Norte, obtaining approval with the Certificate of Presentation for Ethical Appreciation (CAAE) 48990515.0.0000.5292. Data analysis was performed using the Concordance Validation Index (IVC> 0.80). 31 diagnoses / results and 154 nursing interventions were validated, which comprised the terminological subset, among them: Risk of hemorrhage, Agitation, Pain, Risk of infection, Hypothermia, Impaired thermoregulation, Edema, Hematoma, Impaired Skin Integrity and Vital Sign Changed. It is concluded that from the nursing records and identification of clinical findings it was possible to elaborate the ICNP® nursing diagnoses, results and interventions for newborns with peripherally inserted central venous catheters. In addition, the use of the Neuman systems model represented a conceptual-philosophical foundation capable of directing clinical assessment to identify stressors and their possible causes.

Descriptors: Nursing process; Terminology; Nursing Diagnosis; Nursing; Catheterization, Central Venous; Newborn.

LISTA DE QUADROS E DIAGRAMAS

QUADRO 1. Termos da linguagem especializada de enfermagem proveniente dos prontuários e achados clínicos constantes na CIPE® Versão 2019, segundo o Modelo dos Sete Eixos. Natal, RN, 2020. 43

QUADRO 2. Termos da linguagem especializada de enfermagem provenientes dos prontuários clínicos e achados clínicos, relevantes para a prática clínica direcionada ao RN em uso de PICC não constantes na CIPE® Versão 2019. Natal, RN, 2020. 46

QUADRO 3. Estressores, níveis dos estressores, Diagnósticos de Enfermagem da CIPE 2019 e suas definições operacionais em recém-nascidos com Cateter Venoso Central de Inserção Periférica. Natal/RN, 2020 47

QUADRO 4. Estressores, níveis dos estressores, Diagnósticos de Enfermagem da CIPE 2019 em recém-nascidos com Cateter Venoso Central de Inserção Periférica. Natal/RN, 2020. 52

QUADRO 5. IVC dos Diagnósticos, Resultados e Intervenções de Enfermagem da CIPE 2019 em recém-nascidos com Cateter Venoso Central de Inserção Periférica. Natal/RN, 2020. 55

QUADRO 6. Intervenções de Enfermagem da CIPE 2019 em recém-nascidos com Cateter Venoso Central de Inserção Periférica segundo Intervenções de prevenção. Natal/RN, 2020.

LISTA DE FIGURAS

**FIGURA 1.**	Elementos do Modelo de Sistemas de Betty Neuman.	25
**FIGURA 2.**	Modelo de Sistemas de Betty Neuman.	27
**FIGURA 3.**	Modelo de Sete Eixos da CIPE.	31
**FIGURA 4.**	Modelo de referência terminológica para diagnósticos de enfermagem	31
**FIGIRA 5.**	Fórmula de cálculo de amostras para populações finitas.	33
**FIGURA 6.**	Percurso metodológico da pesquisa.	34
**FIGURA 7.**	Diagrama dos estressores e estratégias de prevenção primária, secundária e terciária.	56

SUMÁRIO

1 INTRODUÇÃO 12
2 OBJETIVOS 17
3 FUNDAMENTAÇÃO TEÓRICA 18
3.1 Cateter Venoso Central de Inserção Periférica (PICC) 18
3.2 Sistematização da Assistência de Enfermagem (SAE)/Processo de Enfermagem (PE)..22
3.3 Teoria de Betty Neuman 24
3.4 Classificação Internacional para a Prática de Enfermagem (CIPE®) 28
4. MÉTODO 32
4.1 Tipo de estudo 32
4.2 Cenário da pesquisa 32
4.3 Sujeitos do estudo 32
4.4 Etapas do desenvolvimento da pesquisa 33
4.4.1 Primeira Etapa: identificação dos achados clínicos de neonatos em uso do PICC 35
4.4.2 Segunda Etapa: elaboração dos Diagnósticos, Resultados e Intervenções de Enfermagem 36
4.4.3 Terceira Etapa: Construção dos enunciados diagnósticos, resultados e intervenções 38
4.4.4 Quarta Etapa: Validação de conteúdo dos enunciados de diagnósticos, resultados e intervenções por especialistas 40
4.4.5 Quinta Etapa: Estruturação do subconjunto terminológico da CIPE para recém-nascido com PICC 41
4.5 Aspectos éticos 42
5. RESULTADOS 43
6. DISCUSSÃO 59
7. CONCLUSÃO 71
REFERÊNCIAS 73
ANEXOS 87
APÊNDICES 90

1. INTRODUÇÃO

Os avanços tecnológicos e no cuidado neonatal permitiram a sobrevida de recém-nascidos gravemente enfermos (LIU; CHEN; WANG, 2015). Como parte do processo assistencial intensivo e especializado, a Unidade de Terapia Intensiva Neonatal (UTIN) é um ambiente onde são realizadas condutas e procedimentos essenciais ao tratamento do neonato hospitalizado (PACHECO *et al*., 2020). Nesse contexto, a prática clínica demanda a realização de procedimentos invasivos, incluso a utilização de cateteres venosos centrais. As indicações incluem monitoramento hemodinâmico, administração de fluidos ou hemoderivados e infusão de medicamentos hiperosmolares (DUESING; FAWLEY; WAGNER, 2016).

O Cateter Venoso Central de Inserção Periférica (CCIP) ou *Peripherally Inserted Central Venous Catheter* (PICC) é destaque entre os cateteres centrais inseridos em UTIN. Seu uso é amplamente aceito por reduzir as complicações associadas aos cateteres centrais convencionalmente usados. Além disso, o PICC pode ser inserido à beira do leito do paciente sem a necessidade de intervenção cirúrgica. Por permitir que a terapêutica medicamentosa seja realizada, o dispositivo é capaz de salvar a vida de neonatos criticamente enfermos (YU *et al*., 2018).

Após o Cateter Venoso Umbilical (CVU), o CCIP é a primeira opção para tratamento de médio a longo prazo, desde o primeiro dia de vida ou a qualquer momento durante toda a internação na UTIN. Os locais comuns de inserção do PICC são as veias periféricas dos membros superiores (cefálica basilar, axilar, veias do antebraço), dos membros inferiores (veia safena parva ao nível da veia poplítea ou veia safena magna ao nível do maléolo interno) e raramente, outras, como a veia jugular externa e a veia auricular posterior (KONSTANTINIDI *et al*., 2019). O PICC tem sua ponta alojada na veia cava superior, ou veia cava inferior, se inserido nos membros inferiores (WESTERGAARD; CLASSEN; WALTHER-LARSEN, 2012).

Aliada a segurança na administração prolongada de soluções intravenosas, o cateter supracitado causa fenômenos inflamatórios menos irritativos ao endotélio vascular. Contudo, o PICC não é isento de riscos e os principais problemas são a infecção ou bacteremia a ele associada, juntamente com outras complicações do tipo mecânico, como obstrução, extravasamento, ruptura do cateter e remoção não eletiva (PADILLA-SÁNCHEZ *et al*., 2019).

O procedimento de inserção do cateter epicutâneo consiste em prática avançada e de alta complexidade. Assim o enfermeiro necessita de competência técnica e legal para realizá-lo. O enfermeiro capacitado é responsável pela inserção, fixação, manutenção e retirada do cateter. O respaldo ético e legal para realização do procedimento no País encontra-se amparado pela Resolução COFEN nº 258/2001, atualizada pelo Parecer COFEN N°243/2017 (COFEN, 2001; COFEN, 2017).

O manejo do cateter PICC pressupõe habilidades e competências que favoreçam a segurança do recém-nascido durante todo o tratamento e que reduzam os riscos de complicações associadas ao cateter. Nesse ínterim, o enfermeiro vivencia um desafio na edificação do conhecimento sobre o qual se fundamenta sua prática assistencial. Nesta diretiva, como ferramenta que organiza a prática e direciona as necessidades do paciente, a Sistematização da Assistência de Enfermagem (SAE) possui significativo impacto na qualidade da assistência direcionada ao paciente (SOARES *et al*., 2015).

No tocante a SAE, como forma de sistematizar a assistência de Enfermagem, tem-se o Processo de Enfermagem (PE), o qual é um instrumento metodológico que conduz a organização do cuidado. O PE oferece suporte para a tomada de decisão e organização do cuidado. A aplicação do PE constitui-se um saber específico e necessário à identidade profissional, o mesmo, é regulamento pela Resolução n. 358/2009 do Conselho Federal de Enfermagem (PIMENTA; SOUZA, 2017; COFEN 2009)

O Processo de Enfermagem é realizado pelo enfermeiro em sua prática clínica através das seguintes etapas: coleta de dados, diagnóstico, planejamento, implementação e avaliação; e, para isso, se apropria de terminologias de enfermagem na realização dos seus registros (VIEIRA *et al*., 2017).

Assim, pontua-se que o PE, é uma metodologia que proporciona uma prática de cuidados sistematizada e organizada, uma vez que garante especificidade e evidência às ações, em conjunto com seu caráter valorativo, organizativo e resolutivo da assistência (VIEIRA *et al*., 2017).

Para a operacionalização do Processo de Enfermagem, é fundamental que o enfermeiro utilize uma terminologia comum e padronizada. Para isso, o profissional pode fazer uso dos chamados sistemas de classificação conhecidos internacionalmente. Dentre os sistemas de classificação mais conhecidos no Brasil,

tem-se a Classificação Internacional para a Prática de Enfermagem (CIPE®) (GARCIA; NÓBREGA, 2013; MATTEI *et al*., 2011).

A CIPE® emerge como um instrumento que auxilia o raciocínio clínico e a tomada de decisão, favorece a comunicação entre enfermeiros e destes com outros profissionais de saúde, e permite a documentação do cuidado prestado ao paciente, fator relevante para a avaliação da assistência ofertada quanto para a visibilidade da Enfermagem (GARCIA, 2019).

A fim de otimizar a utilização da CIPE®, com intuito de tornar-se uma referência de fácil acesso ao profissional de enfermagem em seu contexto profissional, a CIE (Conselho Internacional de Enfermeiros) sugeriu a construção de subconjuntos terminológicos ou catálogos, representados pelos diagnósticos (DE), resultados (RE) e intervenções de enfermagem (IE) direcionados para as necessidades de saúde do paciente. Dessa forma, os subconjuntos terminológicos são considerados ferramenta de apoio à documentação sistemática e suporte à prática do profissional enfermeiro (CARVALHO; CUBAS; NÓBREGA, 2017; ICN, 2008).

O Centro de Pesquisa e Desenvolvimento da CIPE® no Brasil elaborou subconjuntos para o cuidado de enfermagem, e para padronizar a elaboração deles, propôs um método para os estudos em andamento. O método de Brasileiro oferece subsídio para que se tenha início à construção do subconjunto terminológico, e é composto por três etapas, a saber: identificação da clientela e/ou prioridade de saúde; justificativa da importância para a Enfermagem; e a escolha do modelo teórico. Em continuidade, estabelece outros passos: a coleta ou identificação dos termos necessários para o desenvolvimento dos enunciados; o mapeamento cruzado entre os termos coletados e os termos da versão da CIPE®; a construção dos novos enunciados, considerando a Terminologia Especializada, o Modelo de 7 eixos da CIPE®, a norma ISO 18.104 e o modelo teórico; o mapeamento cruzado entre os enunciados construídos e os conceitos pré-combinados da CIPE®; a validação dos enunciados; e a estruturação do subconjunto (CARVALHO; CUBAS; NÓBREGA, 2017).

Ressalta-se que para o uso de qualquer tipo de prática sistemática de enfermagem, a mesma deve ser sustentada por uma teoria. A presente pesquisa sustentada no terceiro pré-requisito do método supracitado elaborou um subconjunto

terminológico para a assistência de enfermagem ao RN com PICC orientada pela Teoria de Betty Neuman.

No Modelo de Sistemas desenvolvido por Betty Neuman, observa-se dois componentes principais, o estressor e a reação a esse estressor. Em sua teoria, Betty Neuman destaca que o indivíduo, família ou grupo de pessoas é visto como um sistema aberto que busca o equilíbrio entre as variáveis estressantes do meio ambiente, com o objetivo de manter a estabilidade necessária para o bem-estar ideal (NEUMAN, 1995).

As situações estressantes acarretadas pelo PICC podem levar ao desequilíbrio do sistema, logo se torna importante o conhecimento das barreiras protetoras deste sistema com a intenção de fortalecê-las na busca da estabilidade da saúde do recém-nascido.

Ao entender a dinâmica dos recém-nascidos em uso do cateter epicutâneo à luz da Teoria de Betty Neuman, o enfermeiro conseguirá reconhecer as necessidades desses pacientes através da identificação dos fatores estressores a que os mesmos estão expostos, como também as potencialidades promovidas pelas linhas de defesa do sistema, com o intuito de possibilitar o desenvolvimento dos diagnósticos de enfermagem e das intervenções necessárias para o enfrentamento satisfatório dos estressores e para a elaboração de um planejamento com vistas ao cuidado integral.

Como enfermeira e reconhecedora da relevância do cuidar do recém-nascido hospitalizado apoiado na Sistematização da Assistência de Enfermagem (SAE), floresce o progresso para o constructo deste estudo científico. Assim, o interesse pela temática deu-se por meio de inquietações e questionamentos da vivência diária em Unidade de Terapia Intensiva Neonatal.

A fim de analisar o campo da produção cientifica sobre a temática, realizou-se uma busca por produção cientifica sobre o neonato em uso do PICC e o Processo de Enfermagem nas bases de dados informatizadas da Biblioteca Virtual em Saúde: Literatura Latino-Americana e do Caribe (Lilacs) e Literatura Internacional em Ciências da Saúde e Biomédica (Medline); Scopus, CINAHL, utilizando os descritores Processos de enfermagem; Terminologia; Diagnóstico de enfermagem; Enfermagem; Classificação; Cateterismo Venoso Central. Verificou-se a predominância de estudos quantitativos voltados, em sua maior parte, às complicações relacionadas ao cateter (OLIVEIRA; RODAS, 2017; SILVA et al., 2017;

SPIRONELLO; CUMAN, 2019; FERREIRA et al., 2020; PRADO *et al*., 2020), o que aponta uma lacuna na literatura acerca da construção de diagnósticos de enfermagem e intervenções voltadas às suas necessidades.

Em vista de sua importância como prestador da assistência ao recém-nascido em uso do Cateter Central de Inserção Periférica e por ser responsável pelo manejo do cateter durante todo o tratamento do paciente, o enfermeiro possui papel relevante na construção de um subconjunto diagnóstico CIPE® para instrumentalizar o profissional na organização da assistência de enfermagem voltada a essa clientela. Destarte, a relevância dessa pesquisa fundamenta-se na necessidade de aprofundar o conhecimento sobre o processo de enfermagem, visando prestar ao RN com PICC uma assistência de qualidade e com foco na redução de danos.

Frente à lacuna encontrada na literatura, elaborou-se a seguinte questão norteadora: Que enunciados de diagnósticos/resultados e intervenções de enfermagem são úteis à assistência de enfermagem a recém-nascidos com Cateter Venoso Central de Inserção Periférica em Unidade de Terapia Intensiva Neonatal?

2. OBJETIVOS

2.1 Objetivo Geral

Desenvolver um Subconjunto Terminológico da CIPE® para recém-nascidos com Cateter Venoso Central de Inserção Periférica, tendo como suporte teórico o Modelo de Sistemas desenvolvido por Betty Neuman.

2.2 Objetivos Específicos:

- Identificar termos da linguagem especial de enfermagem para o cuidado de recém-nascido com Cateter Venoso Central de Inserção Periférica;
- Elaborar enunciados de diagnósticos, resultados e intervenções de enfermagem baseados na CIPE® para o cuidado de recém-nascido com Cateter Venoso Central de Inserção Periférica;
- Validar o conteúdo dos enunciados de diagnósticos, resultados e intervenções de enfermagem elaborados, em relação à sua pertinência, para o cuidado de recém-nascido com Cateter Venoso Central de Inserção Periférica, segundo a opinião de enfermeiros experts.

3. FUNDAMENTAÇÃO TEÓRICA

Nesta seção, serão abordados os seguintes temas: O recém-nascido hospitalizado em uso do Cateter Venoso Central de Inserção Periférica (PICC); Sistematização da assistência de enfermagem e o Processo de Enfermagem; Modelo de Sistemas de Betty Neuman; Classificação Internacional para a Prática de Enfermagem (CIPE®).

3.1. Cateter Venoso Central de Inserção Periférica (PICC)

No início do renascimento, houve a descoberta da circulação sanguínea, e a técnica da terapia venosa ocorreu no mesmo período do surgimento da primeira agulha hipodérmica. Por volta de 1929, foi descoberta à possibilidade de ter acesso a veias centrais, através de uma punção periférica, sendo esse o conceito da técnica de inserção do Cateter Central de Inserção Periférica (PICC) atualmente (DE FREITAS; NUNES, 2009; OLIVEIRA *et al*., 2014). Somente em 1950 foram comprovados os benefícios e a segurança desse procedimento, que iam de contrapartida com as complicações oriundas da punção em vasos centrais, ou seja, uma maior hemodiluição da droga, reduzindo a trombose química e reações inflamatórias na região (LIENEMAN; TAKASHASHI; DOS SANTOS, 2013).

O Cateter Central de Inserção Periférica (PICC) é um dispositivo intravenoso central longo, confeccionado em materiais bioestáveis e biocompatíveis e de baixa trombogenicidade, inserido através de uma veia periférica e posicionado a nível central na veia cava superior ou inferior (DE OLIVEIRA *et al*., 2014).

De acordo com Baggio (2010), o PICC pode conter lúmen único ou duplo. Além disso, os constituídos de silicone são mais flexíveis e causam menor irritação à parede dos vasos e interação medicamentosa. O cateter em tela pode permanecer por período prolongado para administração de antibióticos, analgésicos, nutrição parenteral, quimioterapia, transfusões sanguíneas, além de permitir monitorização hemodinâmica.

No que diz respeito à autonomia dos enfermeiros na terapia venosa, durante a Segunda Guerra Mundial (1941-1945), com a reduzida quantidade de profissionais médicos, as enfermeiras começaram a ser designados à essa atividade, esse grupo de enfermeiros eram chamados de “Enfermeiros IV” (DE FREITAS; NUNES, 2009).

A neonatologia se desenvolveu a partir de 1892 na França. Nessa época surge também a Unidade de Terapia Intensiva Neonatal (UTIN), com um amplo

aporte tecnológico, o que favoreceu a sobrevida de prematuros de baixo peso. Nesse contexto, uma das principais técnicas que proporcionaram a sobrevida dos neonatos prematuros foi o advento da terapia venosa (MONTES *et al*., 2011; MOTTA *et al*., 2011; VIEIRA *et al*., 2013).

Nos Estados Unidos, o PICC começou a ser usado em Unidades de Terapia Intensiva entre 1960 e 1970 e em Unidades de Terapia Intensiva Neonatal somente em 1973 deu-se início a este procedimento por Shaw. Já no Brasil, o PICC é amplamente utilizado desde a década de 1990, principalmente em unidades neonatais e pediatria (FREITAS e NUNES, 2009; OLIVEIRA *et al*., 2014).

No Brasil, a utilização do PICC teve início a partir da década de 90, entretanto, foi regulamentada somente em 2001, pela Resolução 258 de 2001 do Conselho Federal de Enfermagem. Desde então, sua utilização tem se tornado cada vez mais frequente, principalmente em recém-nascidos e crianças hospitalizadas que necessitam de terapia intravenosa por tempo prolongado. No caso do Brasil, a instrução, inserção, manutenção e retirada do PICC cabe particularmente aos enfermeiros, desde que tenham recebido a capacitação teórico-prática (DE OLIVEIRA *et al*., 2014).

A inserção do PICC implica perícia técnica, capacidade de raciocínio clínico e tomada de decisão consciente, segura e eficaz. O PICC é indicado em tratamentos de longa duração (acima de seis dias); manejo de nutrição parenteral, infusão de medicamentos vesicantes, irritantes ou vasoativos; soluções hiperosmolares ou com pH não fisiológico e administração de quimioterápicos (DE OLIVEIRA *et al*., 2014).

Em se tratando de procedimento de instalação do PICC, nota-se que é de autonomia e competência técnica do enfermeiro a instalação do PICC, desde que este detenha de habilitação teórico prática por meio de curso com certificação, porém a manipulação deste é realizada por toda a equipe de enfermagem, este manuseio justifica o processo de educação continuada eficaz para todos os profissionais envolvidos no processo de cuidados (BAGGIO; BAZZI; BILIBIO, 2010; MOTTA *et al*., 2011; VIEIRA *et al*., 2013).

O cuidado de crianças e recém-nascidos com necessidade de terapia intravenosa exige habilidades e conhecimentos específicos do enfermeiro, tais como: características anatômicas e fisiológicas, padrões de crescimento e desenvolvimento, bem como desenvolver diretrizes assistenciais que embasam a prática. Nessa perspectiva, é de grande importância que os enfermeiros utilizem

evidências científicas que norteiam uma prática segura na utilização dos diversos tipos de dispositivos intravenosos (DE OLIVEIRA *et al*., 2014).

O dispositivo PICC vem sendo utilizado de maneira ampla em unidades de terapia neonatal para acesso venoso a médio e longo prazo, suplantando cada vez mais o convencional cateter venoso central (CVC), sendo a primeira escolha para acesso central após o cateterismo umbilical. Especula-se que foi originalmente desenvolvido para uso pela população neonatal, mas atualmente é usado em todas as faixas etárias (BAGGIO; BAZZI; BILIBIO, 2010).

Ao indicar o tipo de dispositivo intravenoso, o enfermeiro avaliará as necessidades de cada paciente com base no julgamento clínico. Assim, os fatores que influenciam a indicação do PICC pelos enfermeiros do cenário do estudo são: diagnóstico clínico, plano terapêutico (com destaque para a antibioticoterapia e a concentração das soluções), prematuridade extrema, baixo peso, obesidade e tempo prolongado de internação (DE OLIVEIRA *et al*., 2014).

As veias basais e cefálicas têm sido designadas como as principais opções devido ao seu menor número de válvulas, maior calibre, terem anatomia favorável e facilitarem a realização e troca de curativos (BAGGIO; BAZZI; BILIBIO, 2010).

O êxito na terapia com o PICC está relacionado a múltiplas variáveis, que envolvem a habilidade técnica, a escolha das veias de punção, a técnica de inserção e os métodos de visualização da rede venosa. Estudo apontou que a indicação precoce do PICC, associada à habilidade técnica enfermeiro e à utilização de métodos de visualização da rede venosa aumentam as taxas de sucesso da inserção em crianças e recém-nascidos (DE OLIVEIRA *et al*., 2014).

Para inserção segura do PICC, o calibre deve ser definido pelo profissional, considerando-se o ajuste do diâmetro do lúmen para o peso e a idade do paciente, sendo adequados os que respeitarem o tamanho do vaso. A literatura aponta critérios a serem considerados na escolha do calibre do cateter, os quais sugerem que crianças pesando menos de 2 Kg devem receber cateter 1.9 Fr (24G); crianças com peso entre 2 e 6 Kg, cateter 2.8 Fr (22G); com peso entre 6 e 20 Kg, cateter 3.0 Fr (20G), e crianças com mais de 20 Kg, cateter 4.0 Fr (18G) (BAGGIO; BAZZI; BILIBIO, 2010).

As principais dificuldades e desvantagens do uso do PICC está relacionada à necessidade de uma rede vascular íntegra e calibrosa para o implante; necessidade de treinamento especial para inserção e manutenção do cateter; monitorização

rigorosa do dispositivo; e necessidade de radiografia para localização da ponta do cateter. Há evidências de que o dispositivo não é imune a complicações como trombose venosa profunda (TVP), tromboflebite, bloqueio do cateter, pseudoaneurisma arterial e infecção. Por outro lado, o emprego desse cateter evita a dissecção venosa e apresenta menor exposição do paciente a dor e complicações inerentes ao procedimento (DI SANTO *et al.,* 2017).

As principais indicações para o implante do cateter foram: antibioticoterapia prolongada (52,0%), NPT (19,3%) e acesso venoso difícil (16,0%). Outras indicações em menor escala foram administração de medicações vesicantes/irritantes (8,0%), risco de sangramento (3,3%) e administração de quimioterápicos (1,4%) (DI SANTO *et al*., 2017).

Durante a inserção o profissional deverá utilizar precauções máximas de barreiras: máscara, gorro, avental estéril, luvas e campos estéreis. Para antissepsia cutânea há indicação de clorexidina como antisséptico de primeira escolha. O cuidado ao neonato deve estar respaldado em evidências confiáveis para que este restabeleça seu estado de saúde no menor tempo necessário. As complicações hospitalares tais como as infecções da corrente sanguínea relacionadas a cateter e tantas outras relacionadas aos dispositivos intravenosos devem ser minimizadas de maneira a oferecer ao neonato e sua família uma permanência hospitalar menos traumática, livre de iatrogenias (JOHANN *et al*., 2012).

Dentre os benefícios do PICC, destaca-se a redução do desconforto do paciente, evitar múltiplas punções venosas, obter via segura para administração de antibióticos; nutrição parenteral prolongada (NPT); excelente via para quimioterápicos; maior tempo de permanência e menor risco de contaminação em relação a outros dispositivos; preservação do sistema venoso periférico; indicação de terapia domiciliar (DI SANTO, 2017).

Dentre as complicações cita-se infecção, trauma, integridade da pele alterada, integridade da pele prejudicada, interação de medicamentos adversa, desequilíbrio na temperatura corporal, reação adversa, embolia, trombose, arritmia, hemorragia, obstrução, hematoma, condição neurológica prejudicada, necrose, lesão do nervo, função do sistema respiratório prejudicada (paralisia diafragmática, derrame, hidrotórax), erosão tissular, função do sistema gastrointestinal prejudicada, edema, desconforto, dor aguda, eritema (MONTES, 2011).

3.2. Sistematização da Assistência de Enfermagem (SAE) e o Processo de Enfermagem (PE)

A Sistematização da Assistência de Enfermagem (SAE) trata-se de uma ferramenta de organização do trabalho, quanto ao método, ao pessoal e aos instrumentos, o que torna possível a operacionalização do processo de Enfermagem, o qual orienta a assistência profissional de Enfermagem e a documentação da prática profissional. A resolução nº 358/2009 do Conselho Federal de Enfermagem (COFEN) é enfática ao identificar a importância do Processo de Enfermagem para a atenção à saúde da população e para o aumento da visibilidade e do reconhecimento profissional (COFEN, 2009).

Inicialmente, na década de 60, o Processo de Enfermagem foi descrito com quatro fases: coleta de dados, planejamento, intervenção e avaliação. Nesta época, já se enfatizava as habilidades intelectuais, interpessoais e técnicas que se consideravam primordiais à prática profissional (YURA; WALSH, 1967). Porém, ainda era incipiente as discussões acerca de diagnosticar por parte do enfermeiro.

A identificação e classificação de diagnósticos de enfermagem culminou em uma revolução no pensamento da área e uma consequente mudança, da ênfase anterior com foco no problema para o raciocínio diagnóstico e pensamento crítico. Tal fato modifica o entendimento do PE, de um processo lógico, linear, de solução de problemas, para um processo dinâmico, em espiral ascendente e recorrente (PESUT; HERMAN, 1999).

Atualmente, o PE organiza-se em cinco etapas inter-relacionadas e interdependentes: 1) coleta de dados, que tem por objetivo obter informações acerca da pessoa, família, ou coletividade humana; 2) diagnóstico de enfermagem, que se refere ao agrupamento dos dados coletados e à interpretação, a qual irá promover a tomada de decisão; 3) planejamento de enfermagem, que identifica as ações e os resultados que se espera alcançar; 4)implementação/realização das ações determinadas durante a fase de planejamento; e 5) avaliação de enfermagem - investigação de mudanças nas respostas do indivíduo, para definir se as intervenções de enfermagem alcançaram os resultados esperados e se há necessidade de alterações nas etapas anteriores (COFEN, 2009).

O processo de enfermagem caracteriza-se como uma ferramenta de reaproximação do enfermeiro com seu cliente. O PE é considerado um instrumento

metodológico de trabalho que permite a análise crítica sobre as condições de saúde do paciente e assegura a atuação dos profissionais de Enfermagem (LEADEBAL; FONTES; SILVA, 2010). A literatura aponta que a não aplicabilidade do PE acarreta o domínio de ações fundamentadas no senso comum e não embasadas cientificamente. Destarte, a falta de planejamento das ações gera desarmonia entre ciência e prática, o que inibe o avanço científico da Enfermagem (ALVES; LOPES; JORGE, 2008).

As resoluções emitidas pelos órgãos reguladores da Enfermagem sustentam as ações que atraem o processo de enfermagem para as discussões e justificam os esforços de sua aplicação nos serviços (DE SOUZA; DOS SANTOS; MONTEIRO, 2013). O amparo legal que iniciou a prática da Sistematização da Assistência de Enfermagem (SAE) no Brasil foi a Resolução 272/2002 do Conselho Federal de Enfermagem – COFEN, que tornou obrigatória a implementação da SAE em toda instituição de saúde, pública e privada. Em seguida a Resolução COFEN 311/2007 reformulou o Código de Ética dos Profissionais de Enfermagem e ressaltou as responsabilidades relacionadas aos registros de Enfermagem, cujas determinações só são viáveis com a prática da sistematização. Mais recentemente, é possível citar a Resolução COFEN 358/2009, que revogou a Resolução nº 272/2002, reforçando a necessidade de implementação da SAE nos serviços de saúde (COFEN, 2002; COFEN, 2007; COFEN, 2009).

A efetivação do Processo de Enfermagem demanda habilidades e capacidades cognitivas, psicomotoras e afetivas, que auxiliam na identificação do fenômeno observado e o seu significado; os julgamentos que são produzidos e os critérios para sua elaboração; e as ações prioritárias e alternativas que o fenômeno exige, para que se alcance o resultado. Esses aspectos dizem respeito aos elementos da prática profissional ligados ao PE: o que os agentes da Enfermagem realizam (ações e intervenções), tendo por base o julgamento sobre necessidades humanas (diagnóstico de enfermagem), para alcançar resultados esperados (resultados de enfermagem) (ICN, 2005).

No contexto do cuidar, o enfermeiro é responsável por diagnosticar as necessidades do paciente, e assim desenvolver um plano individualizado e voltado às reais necessidades do cliente. Tal planejamento deve ser direcionado à integração do indivíduo como um todo a fim de atingir o seu mais alto nível de bem-estar.

3.3. Teoria de Betty Neuman

Aplicável globalmente, o Modelo de Sistemas de Neuman, baseado na teoria geral do sistema, é ancorado no relacionamento contínuo do cliente os fatores de estresse ambientais, os quais têm potencial para causar uma reação ou reação sintomática ao estresse, ou podem afetar a reconstituição após o tratamento de uma reação de estresse. O modelo leva em consideração todas as variáveis que afetam a resposta de um cliente aos estressores e explica como a estabilidade do sistema é alcançada (NEUMAN, 1995).

O principal objetivo da enfermagem é facilitar o bem-estar ideal para o cliente por meio da retenção, obtenção ou manutenção da estabilidade do sistema do cliente. O bem-estar ideal representa o maior grau possível de estabilidade do sistema em um determinado momento (NEUMAN, 1995).

O Modelo de Neuman concentra-se em sistemas abertos vivos. A enfermagem é responsável por definir ações apropriadas em situações relacionadas ao estresse; uma vez que as trocas ambientais são recíprocas, o cliente e o meio ambiente podem ser afetados positiva ou negativamente um pelo outro. A saúde se reflete no nível de bem-estar: quando as necessidades do sistema são atendidas, existe um estado de bem-estar ideal. Por outro lado, as necessidades não atendidas reduzem a condição de bem-estar do cliente (NEUMAN, 1995).

O Modelo representa o cliente em uma perspectiva de sistemas abertos de forma holística e multidimensional. Além disso, ilustra a composição de cinco variáveis de interação - fisiológica, psicológica, sociocultural, de desenvolvimento e espiritual - que funcionam harmoniosamente ou são estáveis em relação às influências estressoras ambientais internas e externas sobre o cliente, como um sistema aberto (NEUMAN, 1995).

O Modelo de Sistemas Neuman se estrutura da seguinte maneira:

- Cada indivíduo ou grupo é um sistema aberto e único; cada sistema é composto de fatores comuns conhecidos ou características inatas dentro de uma faixa normal de resposta contida em uma estrutura básica. O cliente está em constante e dinâmica troca de energia com o meio ambiente.

- As inter-relações específicas das variáveis do cliente - fisiológicas, psicológicas, socioculturais, de desenvolvimento e espirituais - podem afetar o grau

em que um cliente é protegido pela linha flexível de defesa contra possível reação a um único estressor ou uma combinação de estressores.

- Cada cliente possui um nível normal de resposta ao ambiente, que é conhecido como linha de defesa normal ou estado de bem-estar/estabilidade normal. Quando a linha de defesa flexível não é mais capaz de proteger o sistema contra um estressor ambiental, o estressor rompe a linha de defesa normal.

- Dentro de cada sistema do cliente estão fatores de resistência internos, conhecidos como linhas de resistência, que funcionam para estabilizar e retornar o cliente ao estado de bem-estar normal (linha de defesa normal) ou possivelmente a um nível mais alto de estabilidade após uma reação estressante ambiental.

Conforme mostra as figuras 1 e 2, o sistema do indivíduo é caracterizado por uma estrutura básica cercada de círculos concêntricos que representam a estrutura básica do sistema. Esse círculo é protegido por contornos nomeados por: linha normal de defesa, linha flexível de defesa e linhas de resistência que são ativadas à medida que ocorre alguma instabilidade do sistema provocada por algum estressor (NEUMAN, 1995).

Figura 1 – Elementos do Modelo de Sistemas de Betty Neuman

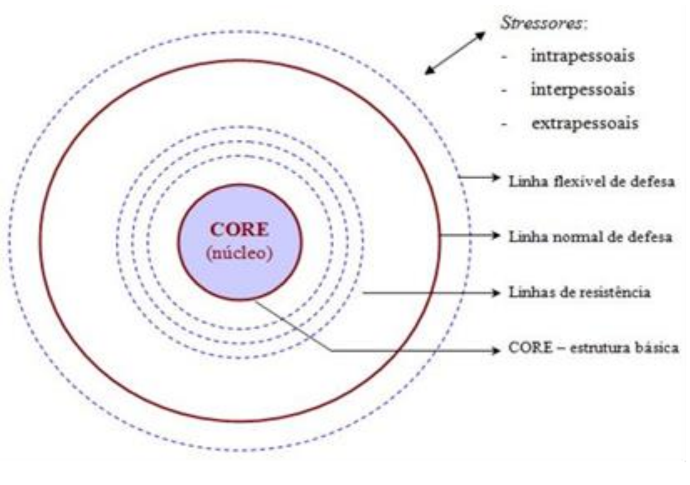

Fonte: Neuman; Fawcet, 2011.

Segundo Neuman, os estressores ambientais são classificados como de natureza intrapessoal, interpessoal e extrapessoal:

• Estressores intrapessoais são forças ambientais internas que ocorrem dentro dos limites do sistema do cliente.

• Estressores interpessoais são forças externas de interação ambiental que ocorrem fora dos limites do sistema do cliente na faixa proximal.

• Estressores extrapessoais são forças externas de interação ambiental que ocorrem fora dos limites do sistema do cliente na faixa distal.

As linhas que compõem o sistema são classificadas da seguinte forma:

As **linhas de resistência** se concentram em torno do anel central e representam recursos que ajudam o cliente a defender-se contra os estressores.

A **linha normal de defesa** é aquilo que a pessoa se torna durante um período da vida – o estado normal de bem-estar ou estado estável – e é composto por fatores fisiológicos, psicológicos, socioculturais, de desenvolvimento e espirituais que são utilizados pelo sistema para lidar com os estressores.

A **linha flexível de defesa** é do tipo "sanfona", e age como um amortecedor para a linha normal de defesa quando o ambiente é ativamente estressante, e como um "filtro, quando o ambiente oferece apoio, e serve como uma força positiva para facilitar o crescimento e o desenvolvimento". Sua eficácia pode ser reduzida por mudanças como perda do sono, má alimentação ou quaisquer alterações nas atividades diárias.

Segundo Neuman (1995), a partir do reconhecimento do estressores, são elencados os níveis de proteção a serem alcançados:

- **Prevenção primária**: está relacionada ao conhecimento geral que é aplicado na avaliação e intervenção do cliente na identificação e redução ou mitigação de fatores de risco possíveis ou reais associados a estressores ambientais para prevenir possíveis reações. Possui como objetivo evitar que a linha normal de defesa seja invadida. As intervenções de enfermagem incluem: a imunização, a educação em saúde, por exemplo.

- **Prevenção secundária**: está relacionada à sintomatologia após uma reação a estressores, classificação apropriada de prioridades de intervenção e tratamento para reduzir seus efeitos nocivos. Concentra-se na linha interna de resistência, com o intuito de proteger a estrutura básica e alcançar a estabilidade do sistema.

- **Prevenção terciária**: está relacionada aos processos de ajuste que ocorrem quando a reconstituição começa e os fatores de manutenção movem o cliente de volta de uma maneira circular em direção à prevenção primária. O objetivo dessa fase é o fortalecimento das linhas de resistência para que o sistema se recupere.

Figura 2 - Modelo de Sistemas de Betty Neuman

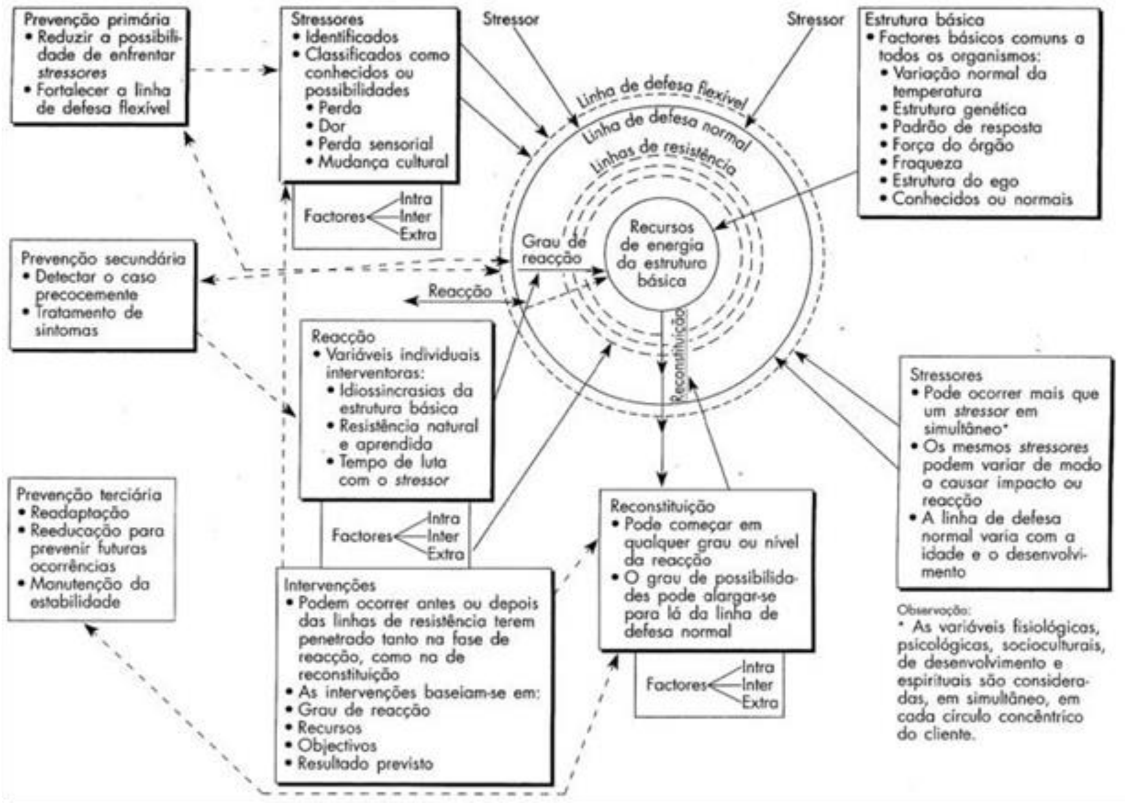

Fonte: TOMEY; ALLIGOOD, 2004

São os seguintes os conceitos principais de teoria de Betty Neuman:

• **Indivíduo** – é um sistema aberto em contato com seu meio e em permanente mudança e movimento, interagindo reciprocamente. É composto de variáveis fisiológicas, psicológicas, socioculturais, de desenvolvimento e espirituais.

• **Ambiente** – conjunto de forças internas e externas que circundam o indivíduo/homem a todo o momento.

• **Saúde/Bem-estar** – é a energia "viva". É um composto dinâmico de equilíbrio físico, psicológico, sociocultural, de desenvolvimento e espiritual.

• **Enfermagem** – uma profissão que se ocupa com todas as variáveis que afetam a reação humana aos estressores, e que devem ser tratadas na sua totalidade. O trabalho da enfermagem visa à manutenção ou recuperação do equilíbrio físico, psicológico, sociocultural, de desenvolvimento e espiritual da "pessoa".

3.4. Classificação Internacional para a Prática de Enfermagem (CIPE®)

A Classificação Internacional para a Prática de Enfermagem - CIPE® é um instrumento de informação para descrever a prática de enfermagem e, consequentemente, prover dados que identifiquem a contribuição da Enfermagem no cuidado da saúde e, ao mesmo tempo, que promovam mudanças na prática de enfermagem através da educação, administração e pesquisa (NÓBREGA; GARCIA, 2005).

Na conferência quadrienal realizada em Seul, Coreia do Sul, em 1989, o Conselho Internacional de Enfermeiras (CIE) propôs a necessidade de desenvolver este sistema de classificação internacional devido à falta de um sistema de classificação da linguagem da profissão, necessário para que a Enfermagem pudesse contar com dados confiáveis na formulação de políticas de saúde, no gerenciamento de custos, na informatização dos serviços de saúde e no controle de seu próprio trabalho (NÓBREGA; GARCIA, 2005).

A CIPE® está sendo desenvolvida como uma estrutura unificadora dos diversos sistemas de classificação em Enfermagem, permitindo a configuração cruzada dos termos das classificações existentes e de outras que forem desenvolvidas. Dessa forma, o CIE reitera que um dos principais critérios da classificação é que ela possa ser ampla o suficiente e sensível à diversidade cultural de modo que sirva para os múltiplos fins e aos propósitos requeridos pelos diferentes países que a utilizam (NÓBREGA; GARCIA, 2005).

As metas iniciais da CIPE® foram revistas durante o planejamento da Versão Beta e da Versão Beta 2. Essas metas, que continuam a direcionar os pressupostos do Programa CIPE®, são: estabelecer uma linguagem padronizada para a prática de enfermagem; representar os conceitos usados na prática; descrever os cuidados de enfermagem prestados às pessoas (indivíduos, famílias e comunidades) no âmbito mundial; possibilitar a comparação de dados de enfermagem entre populações de clientes, em ambientes, áreas geográficas e tempos diversos; estimular a pesquisa por meio da vinculação de dados disponíveis em sistemas de informação de enfermagem e de saúde; propiciar dados sobre a prática, de modo a influenciar a educação em enfermagem e políticas de saúde; projetar tendências sobre as necessidades dos pacientes, a provisão de tratamentos de enfermagem,utilização de recursos e resultados do cuidado de enfermagem (NÓBREGA; GARCIA, 2005).

O foco central da CIPE® é a prática de enfermagem, descrita como um processo dinâmico, sujeito a mudanças, tendo como componentes principais os fenômenos, as ações e os resultados de enfermagem, num enfoque multiaxial. Esse enfoque permite combinações de conceitos dos distintos eixos, proporcionando maior solidez à classificação e diversificando a expressão de seus conceitos (NÓBREGA; GARCIA, 2005).

Em 1991, foi realizado um levantamento na literatura da área e uma pesquisa junto às associações filiadas ao CIE, para identificar, em âmbito internacional, os sistemas de classificação usados na Enfermagem, esses sistemas eram utilizados para descrever os elementos da prática profissional e se valorizava a ideia do desenvolvimento de um sistema de classificação que representasse a Enfermagem mundial (MAZONI *et al*., 2010).

Em 1993, o CIE divulgou o documento Próximo Avanço da Enfermagem: uma Classificação Internacional para a Prática de Enfermagem – Um Documento de Trabalho (*Nursing's Next Advance: An International Classification for Nursing Practice (ICNP) – a Working paper*), com uma listagem de termos, identificados na literatura que eram usados para descrever as intervenções, diagnósticos e resultados de enfermagem (MAZONI *et al.,* 2010).

Na década de 90, o CIE publicou a verão Alfa da CIPE: um marco unificador, contendo a Classificação de Fenômenos de Enfermagem e a Classificação de Intervenções de Enfermagem, com o objetivo de estimular comentários, observações, críticas e recomendações de ajuste e, assim, iniciar um processo de retroalimentação com vistas ao seu aprimoramento. Na evolução, foi divulgada a CIPE Versão Beta, em 1999, durante as comemorações dos 100 anos do CIE; a CIPE Versão Beta 2, em 2001; a CIPE Versão 1.0, em 2005; e a CIPE Versão 1.1 (CIE, 2005).

Em 2000, o CIE estabeleceu o Programa CIPE que tem como uma das metas adaptar o trabalho de desenvolvimento da CIPE às normas internacionais, e de maneira compatível com outras disciplinas da área da saúde. Sendo assim, à CIPE facilita a comunicação das enfermeiras sobre sua prática, seja entre si, com outros profissionais e/ou com formuladores de políticas de saúde, facilita a padronização da documentação do cuidado prestado ao paciente, facilita o intercâmbio de dados entre populações, entre outros aspectos fundamentais para as práticas do enfermeiro (CIE, 2007).

O modelo de sete eixos da CIPE® norteia o enfermeiro na elaboração de um diagnóstico de enfermagem. Para isso, é indicado a inclusão de um termo do eixo foco e outro do eixo julgamento. Caso necessário podem ser incluídos termos adicionais dos eixos citados ou dos demais. Na composição das intervenções de enfermagem, recomenda-se a inclusão de um termo do eixo ação e pelo menos um termo alvo. Considera-se termo alvo qualquer termo de um dos eixos, exceto do eixo julgamento. Nesse caso, termos adicionais para o eixo de ação ou outros eixos também podem ser incluídos (DA MATA *et al*., 2012).

A Versão 1.0 da CIPE® tem inúmeros propósitos: identificar similaridades e diferenças entre diferentes representações, de modo a comparar dados de diferentes fontes; facilitar o desenvolvimento de vocabulários locais; preencher uma necessidade prática de construir sistemas de registro eletrônicos do paciente, com todos os benefícios de fazer parte de um sistema de linguagem unificada (NÓBREGA; GARCIA, 2005).

A CIPE® Versão 1.0 possui sua estrutura de classificação organizada em sete eixos definidos como: foco - área de atenção relevante para a Enfermagem; julgamento - opinião clínica ou determinação relacionada ao foco da prática de enfermagem; meios - maneira ou método de executar uma intervenção; ação - processo intencional aplicado a, ou desempenhado por um cliente; tempo - o ponto, período, momento, intervalo ou duração de uma ocorrência; localização - orientação anatômica ou espacial de um diagnóstico ou intervenção; e cliente - sujeito a quem o diagnóstico se refere e que é o beneficiário de uma intervenção de enfermagem (NÓBREGA; GARCIA, 2005).

O Modelo de Sete Eixos busca facilitar a composição de afirmativas organizadas em grupos significativos, de modo que se tenha acesso rápido a conjuntos de enunciados preestabelecidos de diagnósticos, intervenções e resultados de enfermagem - Catálogos CIPE® (NÓBREGA; GARCIA, 2005).

Figura 3 - Modelo de Sete Eixos da CIPE

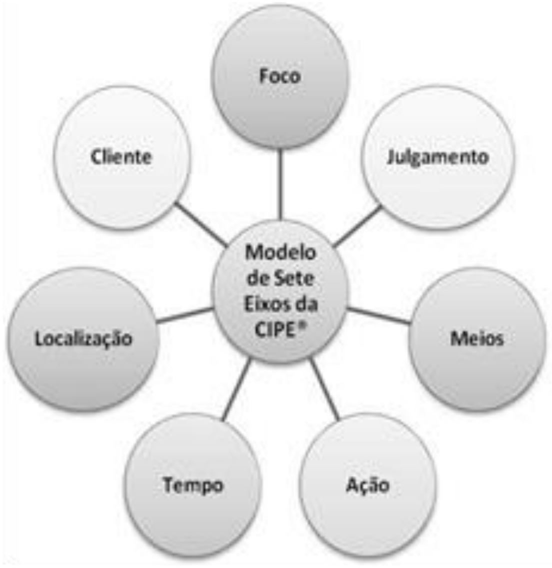

Fonte: ISO 18104:2003, Genebra, Suíça, p.6

Figura 4 – Modelo de referência terminológica para diagnósticos de enfermagem

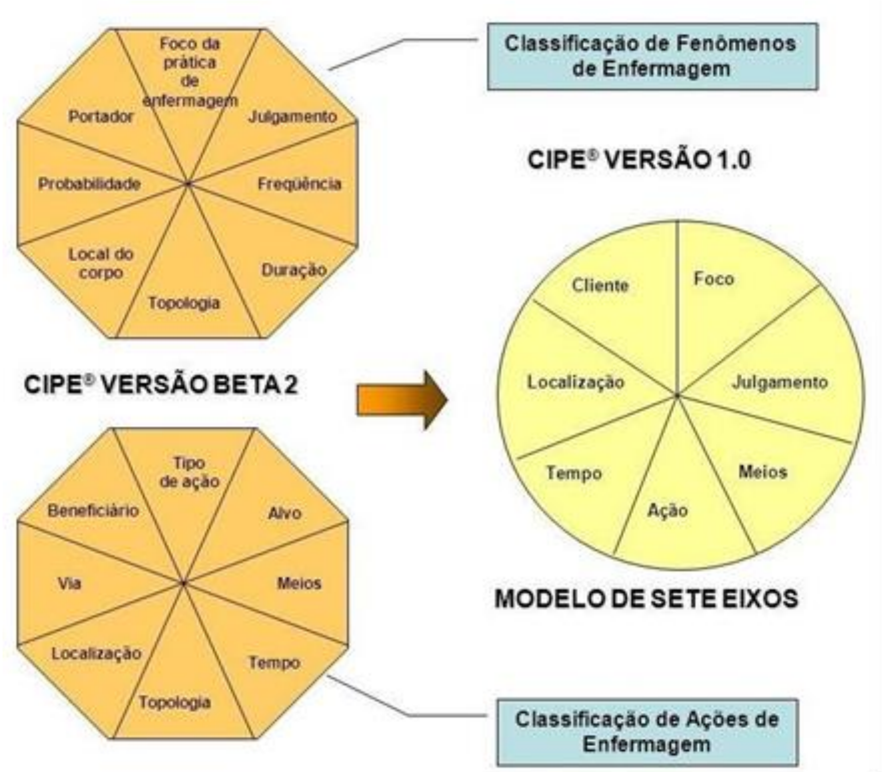

Fonte: ISO 18104:2003, Genebra, Suíça, p.3

4. MÉTODO

4.1. Tipo de estudo

Trata-se de um estudo metodológico para o desenvolvimento e validação de um subconjunto terminológico da CIPE® para recém-nascidos com Cateter Venoso Central de Inserção Periférica (PICC).

Os estudos metodológicos tratam do desenvolvimento, da validação e da avaliação de ferramentas e métodos de pesquisa (POLIT; BECK, 2011).

4.2. Cenário da pesquisa

A presente pesquisa foi desenvolvida na Unidade de Terapia Intensiva Neonatal da Maternidade Escola Januário Cicco, na cidade de Natal/RN, Nordeste, Brasil. A instituição citada é uma das filiais da Rede Hospitalar da Empresa Brasileira de Serviços Hospitalares – Ebserh e é uma instituição de Assistência, Ensino, Pesquisa e Extensão em Saúde (EBSERH, 2019).

A Maternidade Escola Januário Cicco é reconhecida como centro de formação de especialistas e irradiadora da ciência ginecológica, obstétrica e materno infantil, com um importante papel ao prestar um atendimento de alto nível à população do Rio Grande do Norte. A maternidade é um campo de conhecimento teórico-prático para docentes e alunos dos cursos de graduação e pós-graduação dos departamentos de saúde da Universidade Federal do Rio Grande do Norte – UFRN (EBSERH, 2019).

Na UTIN ficam internados os recém-nascidos prematuros ou que nasceram com algum problema e necessitam de cuidados especializados. Possui 25 leitos, sendo divididos em 20 leitos na UTIN e 05 de UCINCO (Unidade de Cuidado Intermediário Convencional) (EBSERH, 2019).

4.3. Sujeitos do estudo

A população foi composta pelos recém-nascidos internados na UTIN em uso do PICC. A população de recém-nascidos internados na UTI Neonatal que utilizaram PICC, conforme dados fornecidos pelo Centro de Controle de Infecção Hospital (CCIH), no ano de 2018 foi de 181 pacientes.

Para estabelecer a amostra do estudo, utilizou-se a fórmula de cálculo de amostras para populações finitas (figura 5), segundo Vieira, a fim de proporcionar de maneira imparcial, segura e científica a eficiência e eficácia desta pesquisa (VIEIRA, 2010).

Figura 5 – Fórmula de cálculo para amostras de populações finitas

$n= \frac{\partial^2 . p . q . N}{e^2 . (N-1) + \partial^2 . p . q}$	LEGENDA:<br>N= universo<br>n=amostra que será calculada<br>∂=nível de confiança<br>e= erro amostral<br>p.q=porcentagem pelo qual o fenômeno ocorre.

O tamanho da amostra foi calculado através do seguinte procedimento. Atribuiu-se um α=5% e margem de erro de 0,05. Como a proporção era desconhecida foi considerado o p=0,5 (variância máxima). A partir da aplicação da fórmula encontrou-se uma amostra de 124 neonatos.

A seleção foi por conveniência de forma consecutiva, adotando-se os seguintes critérios elegíveis de inclusão: recém-nascido (RN) internado na UTI neonatal em uso de PICC e que permaneceram na UTIN até o término do tratamento. Os critérios de exclusão foram: transferência do RN para outra instituição.

Além dos 124 recém-nascidos participantes da pesquisa, outros sujeitos compuseram o estudo que foram os enfermeiros especialistas. Para a busca dos mesmos utilizou-se a plataforma Lattes, do Conselho Nacional de Desenvolvimento Científico e Tecnológico (CNPq) profissionais que pesquisavam nas respetivas Área: enfermagem; Subárea: cuidados intensivos neonatal; sistematização da assistência de enfermagem; Classificação Internacional para a Prática da Enfermagem (CIPE®).

Para o cálculo do número de peritos, adotou-se o referencial metodológico proposto por Lopes, Silva, Araujo (2013) utilizando-se a fórmula: $n= Z\alpha 2 * P^* (1 - P)/e^2$, em que "Zα" se refere ao nível de confiança adotado (95%); "P" representa a proporção de especialistas que indicam a adequação dos itens (85%); e "e" representa a diferença proporcional aceitável em relação ao que pode ser esperado (15%). Portanto, como o coeficiente Zα de acordo com o padrão normal de distribuição assume um valor tabulado de 1.96 para um nível de confiança de 95%, o cálculo do tamanho amostral ao final foi definido por n=1.962*0.85*0.15/0.152 = 22 especialistas.

4.4. Etapas do desenvolvimento da pesquisa

O presente estudo transcorreu com as seguintes etapas:

1ª etapa: identificação dos achados clínicos de neonatos em uso do PICC; 2ª etapa: elaboração dos Diagnósticos, Resultados e Intervenções de Enfermagem; 3ª etapa: Construção dos enunciados diagnósticos, resultados e intervenções; 4ª etapa: Validação de conteúdo dos enunciados de diagnósticos, resultados e intervenções por especialistas; e 5ª etapa: Estruturação do subconjunto terminológico da CIPE para recém-nascido com Cateter Venoso Central de Inserção Periférica.

Figura 6 - Percurso metodológico da pesquisa, Natal, RN, 2020.

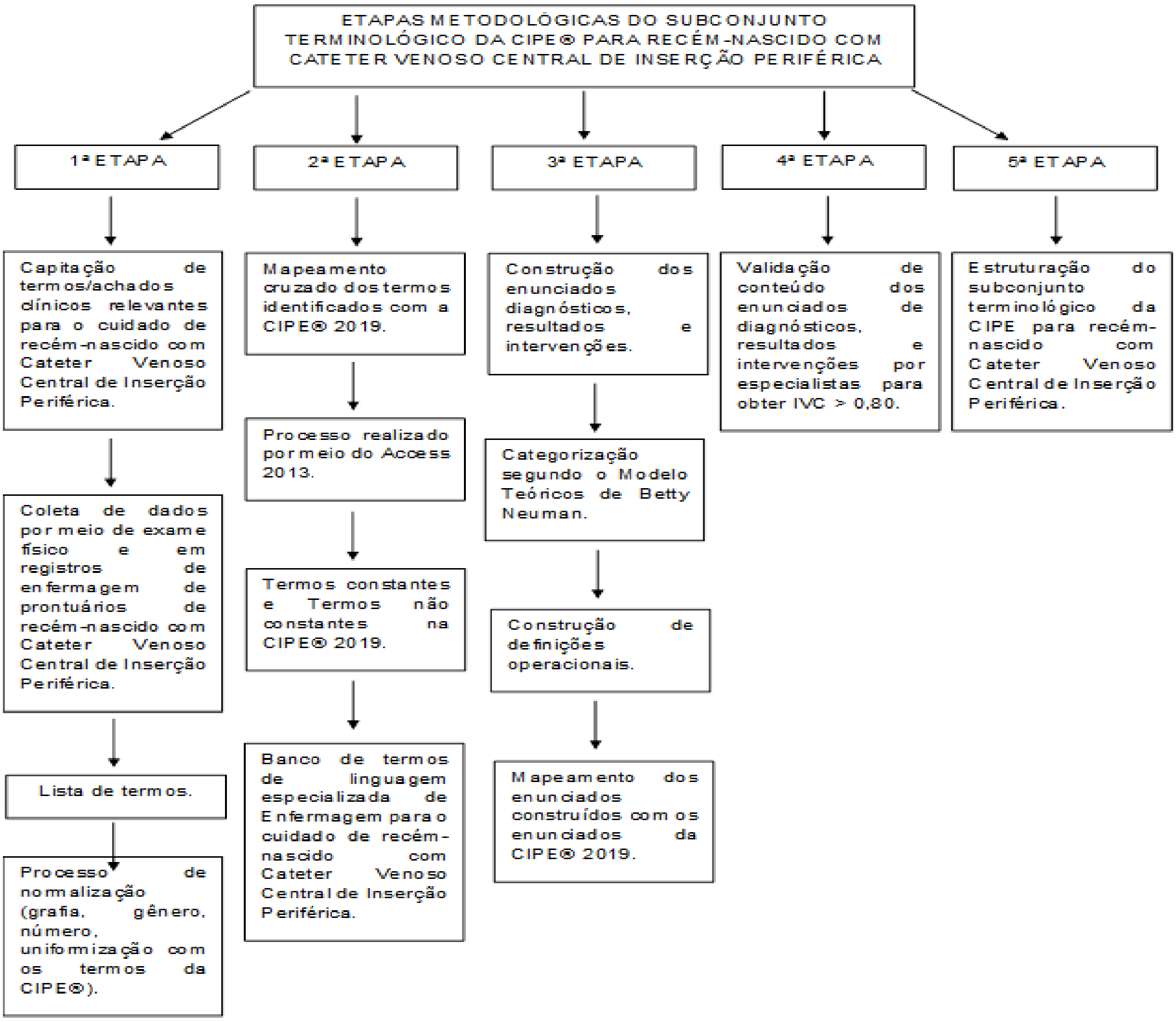

4.4.1. Primeira Etapa: Capitação de termos/achados clínicos relevantes para o cuidado de recém-nascido com Cateter Venoso Central de Inserção Periférica.

Com o objetivo de identificar os termos/achados clínicos relacionados ao uso do Cateter Venoso Central de Inserção Periférica em recém-nascidos, a pesquisa foi direcionada pela seguinte questão norteadora: Quais os termos/achados clínicos são relevantes para o cuidado de recém-nascido com Cateter Venoso Central de Inserção Periférica?

Nesta etapa, os dados foram coletados por meio de exame físico e em registros de enfermagem de prontuários de recém-nascido com Cateter Venoso Central de Inserção Periférica. Assim, foram examinados 124 recém-nascidos que foram submetidos à inserção do PICC no período de janeiro a dezembro de 2019. Da mesma forma também foram coletados dados em seus respectivos prontuários.

A coleta foi realizada de forma manual por uma única pesquisadora. Tais registros foram transcritos pelo próprio pesquisador para um computador na ferramenta Word® em texto único que foi convertido, em seguida, para um documento em formato PDF, o qual constituiu nesse instante o corpus do estudo denominado “PDF-P”.

A extração dos termos contidos nos registros dos prontuários clínicos ocorreu de maneira automatizada por meio de uma ferramenta denominada PorOnto (ZAHRA; CARVALHO; MALUCELLI, 2013). Esse processo de extração iniciou-se a partir do envio do arquivo “PDF-L” à ferramenta PorOnto que, automaticamente, o processou e logo depois gerou uma planilha em Excel® contendo tanto conceitos simples (compostos por apenas um termo, como um substantivo, verbo ou mesmo acrônimos) quanto conceitos complexos (compostos por mais de um termo, como locuções verbais, locuções adverbiais, substantivos compostos) provenientes da literatura. Em seguida, novo processo de extração foi aplicado ao arquivo “PDF-P”, que resultou em uma outra planilha em Excel® contendo também conceitos simples e conceitos complexos provenientes dos prontuários clínicos.

Dentre os termos extraídos, foram selecionados substantivos, adjetivos, verbos e advérbios com base na frequência de aparição e pertinência com a temática da pesquisa, os quais compuseram duas listas de termos identificados como relevantes para o cuidado desses pacientes: uma lista com 64 achados

clínicos provenientes dos exames físicos e outra lista contendo 3.918 termos oriundos dos registros de enfermagem nos prontuários clínicos.

Estas listas foram submetidas a um processo de normalização em separado, que consistiu na padronização das flexões de gênero, número e grau dos substantivos e adjetivos, bem como das flexões verbais, com a finalidade de identificar e remover repetições de termos para posteriormente, serem analisadas e tais termos serem julgados como relevantes ou não relevantes pelos enfermeiros, especialistas. Desta maneira, esta etapa resultou ao final em duas listas de termos relevantes para o cuidado de recém-nascido com Cateter Venoso Central de Inserção Periférica, sendo uma proveniente dos exames físicos com 48 termos e outra dos prontuários clínicos ora pesquisados com 794 termos.

4.4.2. Segunda Etapa: Mapeamento dos Termos identificados com a CIPE® versão 2019

Nessa etapa, os termos identificados nos prontuários e os achados clínicos capitados por meio de exame físicos passaram por um processo de mapeamento cruzado, ou seja, foram analisados e comparados manualmente com os termos da CIPE® 2019.

Segundo Lucena; Barros (2005), o mapeamento cruzado ou cross-mapping consiste em um procedimento metodológico que possibilita mapear e comparar registros de enfermagem realizados com terminologias não uniformizadas em classificações de referência com linguagem padronizada, fornecendo uma explicação acerca de algo que ainda não se dispõe e que pode ser obtida a partir da determinação de semelhanças e diferenças entre termos, além da análise de dados em diferentes níveis de abstração.

Nesse mapeamento cruzado, cada lista de termos relevantes foi submetida, em separado, a um processo de cruzamento com os termos contidos na CIPE® versão 2019, por meio da ferramenta Access for Windows® Versão 2013, resultando em termos constantes e não constantes no modelo de 7-Eixos dessa terminologia.

Por fim, a lista de termos relevantes para o cuidado de recém-nascido com Cateter Venoso Central de Inserção Periférica apresentou, nesse primeiro momento, 28 termos constantes e 20 termos não constantes oriundos dos achados clínicos, enquanto a lista advinda dos registros da equipe de enfermagem em prontuários

clínicos evidenciou 284 termos constantes e 610 termos não constantes nessa classificação.

Os termos não constantes na CIPE® versão 2019 provenientes dos prontuários e os achados clínicos foram analisados em separado pelo pesquisador quanto à similaridade e abrangência em relação aos termos do sistema de classificação, segundo os critérios propostos por Ehnfors, Florin e Ehrenberg (2003):

a) O termo é considerado similar quando não existe concordância na grafia, mas o seu significado é idêntico ao significado do termo existente na CIPE®;

b) O termo é considerado mais abrangente quando apresenta um significado mais amplo em relação ao significado do termo existente na CIPE®;

c) O termo é considerado mais restrito quando apresenta um significado mais específico em relação ao do termo existente na CIPE®;

d) O termo não apresenta concordância quando ele é totalmente diferente daqueles termos existentes na CIPE® em termos de grafia e significado, caracterizando um termo novo.

Por fim, os termos resultantes desse processo de análise de similaridade e abrangência classificados como similares foram considerados como termos constantes na CIPE® versão 2019, sendo adotadas as próprias denominações e definições já existentes nessa terminologia, enquanto os termos analisados e classificados como mais abrangentes, mais restritos e sem concordância foram considerados como termos não constantes no sistema de classificação.

As listas resultantes de termos foram oportunamente integradas e submetidas a um processo de remoção de repetições, revisão e seleção de termos para compor uma listagem única de termos constantes e não constantes na CIPE®, destinada à construção de enunciados de diagnósticos, resultados e intervenções de enfermagem.

Finalmente, os termos constantes e não constantes na CIPE® Versão 2019, enquanto produto do processo de mapeamento, foram classificados por eixo da CIPE® e compuseram a Terminologia Especializada de Enfermagem, o qual potencializou a construção de enunciados de diagnósticos, resultados e intervenções de enfermagem.

4.4.3. Terceira Etapa: Elaboração dos enunciados de Diagnósticos/Resultados e Intervenções de Enfermagem

Esta etapa corresponde a construção dos enunciados de diagnósticos/resultados (DE/RE) e intervenções (IE) de enfermagem que, de acordo com as orientações advindas do CIE, se baseiam no Modelo de terminologia de referência da norma ISO 18104:2014; no Modelo de 7-Eixos da CIPE® Versão 2019 e na Terminologia Especializada de Enfermagem voltado para o cuidado de recém-nascidos em uso de PICC. Além disso, utilizou-se a teoria de Neuman.

A construção dos enunciados de DE/RE ocorreu a partir da combinação entre termos do Banco de termos da linguagem especializada de enfermagem voltado para o cuidado de recém-nascidos em uso de PICC, obedecendo à norma ISO 18104:2014, tida como um modelo integrador de terminologias que estabelece que, para a construção de um diagnóstico de enfermagem, é imperioso o emprego de um termo relacionado ao Foco e de um termo relacionado ao Julgamento; de um descritor único que equivalha como Foco e Julgamento; ou de apenas um Achado Clínico que possa representar estado alterado, função alterada ou mesmo modificação no comportamento, tal como a definição declarada de achados clínicos presente na norma ISO/TS 22789:2010 (INTERNATIONAL ORGANIZATION FOR STANDARDIZATION, 2010, 2014).

Após a construção dos enunciados de DE/RE, foi executada a técnica de mapeamento cruzado com os enunciados de conceitos pré-coordenados de DE/RE da CIPE® Versão 2019, o que resultou em enunciados constantes e não constantes na CIPE® 2019.

Para os enunciados de DE/RE não constantes, foi realizado um processo de análise quanto à similaridade e abrangência em relação aos enunciados de DE/RE constantes na CIPE® Versão 2019, utilizando-se os critérios propostos por Ehnfors, Florin e Ehrenberg (2003):

a) O enunciado é considerado similar quando não existe concordância na grafia, mas o seu significado é idêntico ao do enunciado existente na CIPE®;

b) O enunciado é considerado mais abrangente quando apresenta um significado mais amplo em relação ao significado do enunciado existente na CIPE®;

c) O enunciado é considerado mais restrito quando apresenta um significado mais específico em relação ao significado do enunciado existente na CIPE®;

d) O enunciado não apresenta concordância quando este é totalmente diferente de qualquer enunciado existente na CIPE®, constituindo, assim, um enunciado de DE/RE novo.

Os enunciados de DE/RE resultantes do processo de análise de similaridade e abrangência classificados como similares foram considerados como enunciados constantes na CIPE®, sendo, porém, adotados os enunciados tais como se apresentam na CIPE® Versão 2019. Enunciados de DE/RE considerados mais abrangentes, mais restritos ou que não apresentam concordância foram considerados como não constantes na CIPE®, sendo todos eles (DE/RE constantes e não constantes) posteriormente classificados de acordo com a Teoria de Neuman.

Tão logo os enunciados de DE/RE construídos foram classificados segundo Neuman, elaboraram-se definições operacionais para aqueles enunciados constantes na CIPE® que não possuíam definições, bem como para os enunciados de DE/RE não constantes na perspectiva de representar o conceito na literatura e no cotidiano da prática profissional na UTI Neonatal. Para tanto, a pesquisadora utilizou dicionários técnicos e da língua portuguesa, literatura das áreas da saúde e da Enfermagem, somado aos artigos científicos sobre a temática em questão, disponível em bases de dados eletrônicas por meio de revisão da literatura.

Ressalta-se que foram utilizados os passos preconizados por Waltz, Strickland e Lenz (2017) que enfatizam o desenvolvimento de uma definição preliminar; revisão da literatura; desenvolvimento ou identificação de características específicas; mapeamento do significado do conceito; e afirmação da definição operacional.

Destaca-se que para os enunciados de DE/RE propostos que já pertenciam à Classificação CIPE® versão 2019, para todos eles automaticamente já foram construídos enunciados de intervenções de enfermagem (IE), tendo como bases empíricas: a Terminologia Especializada e os enunciados de DE/RE voltados para o cuidado de RN em uso de PICC; a norma ISO 18104:2014; o Modelo de 7-Eixos e a lista de IE da CIPE® Versão 2019; a Teoria de Neuman.

A construção dos enunciados de IE ocorreu a partir da combinação entre termos da Terminologia Especializada de enfermagem, obedecendo à norma ISO 18104:2014, que vislumbra que em toda intervenção de enfermagem deve haver um descritor para a ação que vai ser realizada (via de regra na CIPE®, um termo do eixo Ação do Modelo de 7-Eixos) e, pelo menos, um descritor relacionado ao alvo

dessa ação, que pode ser um termo de qualquer outro eixo, exceto do eixo Julgamento (INTERNATIONAL ORGANIZATION FOR STANDARDIZATION, 2014).

As ações de enfermagem podem, ainda, ser qualificadas pelos meios, vias e período, além do local que deve ser usado para especificar a posição do alvo. Nesse sentido, o alvo é aquele ou aquilo que é afetado pela ação ou que fornece o conteúdo para a ação, levando-se em consideração que, nas ocasiões em que o próprio paciente é quem sofre a ação, o alvo pode ser suprimido da expressão diagnóstica. Os meios são os instrumentos e técnicas usados pelos enfermeiros para realizar o cuidado, e as vias são os percursos selecionados como oral, subcutâneo, intradérmico, intravenoso, dentre outros (MARIN; PERES; DAL SASSO, 2013; INTERNATIONAL ORGANIZATION FOR STANDARDIZATION, 2014).

Assim, foi construída uma lista com 42 diagnósticos/resultados e 158 intervenções de enfermagem.

4.4.4. Quarta Etapa: Validação de conteúdo dos enunciados de Diagnósticos/Resultados e Intervenções de Enfermagem

Após a elaboração dos enunciados diagnósticos/resultados e Intervenções de Enfermagem, estes foram enviados para peritos a fim de julgar a sua adequabilidade. Os 45 especialistas selecionados receberam a carta convite por correio eletrônico, o termo DE Consentimento Livre e Esclarecidos (TCLE) descrevendo os objetivos, sigilo e participação voluntaria, respeitando-se o tempo de resposta que foi similar a 30 dias. Na primeira rodada delpih 42 especialistas responderam o questionário, e na segunda rodada 26.

Por fim, foram enviada uma planilha no Microsoft Excel (Office 2009) de diagnósticos/resultados e Intervenções de Enfermagem, que continha espaços para assinalar frente a concordância, discordância e sugestões para que os enfermeiros especialistas sugerissem mudanças, e que após a análise do pesquisador principal e orientador desta pesquisa, eram devolvidos para uma nova avaliação.

Além disso, para cada diagnósticos/resultados e Intervenções de Enfermagem apresentado no formulário, inseriu-se a escala Likert contendo cinco pontos: 1=totalmente inadequado; 2=moderadamente inadequado; 3=nível de

adequação que se assemelhasse ao de inadequação; 4=moderadamente adequado; e 5=totalmente adequado.

Os dados foram enviados em forma de planilha utilizando o Microsoft Excel aos enfermeiros especialistas. Analisou-se a concordância dos enfermeiros especialistas, por meio do Índice de Validade de Conteúdo (IVC), sendo considerando validados os que obtiveram (IVC) > 0,80.

Calculou-se o Índice de Validade de Conteúdo (IVC), considerando o número de especialistas que participaram do estudo e o valor aceitável do IVC para cada item, o qual variou entre 0,80 e 1,00. Destaca-se que o escore mínimo aceitável para a validade de conteúdo foi de 0,80 (MCMILLAN; KING; TULLY, 2016).

Nesta pesquisa, dos 42 diagnósticos/resultados e das 48 intervenções de enfermagem submetidos ao processo de validação, 31 DE/RE e 154 IN alcançaram IVC > 0,80, sendo considerados validados.

4.4.5. Quinta Etapa: Organização e Estruturação do Subconjunto Terminológico CIPE® para recém-nascidos em uso do Cateter Venoso Central de Inserção Periférica, segundo o Modelo Teórico de Betty Neuman.

Concluída a etapa anterior, a estruturação do Subconjunto terminológico CIPE® seguiu as orientações do CIE, a saber:

a) mensagem aos leitores;

b) importância do subconjunto para a Enfermagem;

c) modelo teórico da estruturação do subconjunto;

d) relação dos enunciados de diagnósticos/resultados e intervenções de enfermagem, dispostos segundo o modelo teórico adotado no estudo;

e) referências ou bibliografia recomendada.

Ressalta-se que utilizou como modelo teórico a Teoria dos Sistemas, proposta por Betty Neuman organizando os diagnósticos/resultados de enfermagem de acordo com os fatores estressores e as intervenções em estratégias de prevenção (primária, secundária e Terciária).

4.5. ASPECTOS ÉTICOS

O estudo obedeceu aos princípios éticos da Resolução nº 466, de 12 de dezembro de 2012, do Conselho Nacional de Saúde (BRASIL, 2012). O projeto de pesquisa foi aprovado pelo Comitê de Ética e Pesquisa do Hospital Universitário Onofre Lopes (CEP-HUOL), com Certificado de Apresentação de Apreciação Ética (CAAE) nº 48990515.0.0000.5292 (Anexo A). O instrumento de coleta de dados dos 108 RN encontra-se no Apêndice A e B.

5. RESULTADOS

A amostra do estudo foi composta por 124 recém-nascidos em uso do Cateter Venoso Central de Inserção Periférica, com predominância do sexo masculino (62,96%), nascidos de parto cesárea (70,37%), baixo peso ao nascer (29,62%) e com média de APGAR de 6,46 e 8,52 no primeiro e quinto minuto de vida, respectivamente. O tempo médio de permanência do PICC foi de 14,8 dias, variando de um a 96 dias.

As principais indicações para a inserção do cateter foram antibioticoterapia (51,85%) e nutrição parenteral (29,62%). Quanto ao diagnóstico clínico, a prematuridade (93,51%) e as afecções respiratórias (82,40%) foram os mais prevalentes.

O tipo de cateter utilizado foi o de único lúmen e de silicone. No tocante aos sítios de inserção, os membros superiores foram os mais prevalentes (62,96%). Todos os PICC foram inseridos por enfermeiros. Em 56% dos registros no prontuário foi relatado o uso de sucção não nutritiva com glicose a 20%, contenção facilitada e, em casos específicos, a sedação, para o manejo da dor durante o procedimento.

Em relação à localização da ponta do cateter após a radiografia, 23,14% ficaram localizados no terço distal da veia cava superior ou da veia cava inferior. Salienta-se que 57,40% dos PICC ficaram localizados a nível intracardíaco, sendo necessário tracionar o cateter a fim de evitar complicações cardíacas.

Em relação aos juízes que validaram o subconjunto, a maioria era do sexo feminino (92,30%), com idade entre 30 e 40 anos (73,07%), residentes na Região Sudeste do Brasil (38,46%), trabalhavam em instituições de ensino (84,61%) e possuíam doutorado (57,69%).

O quadro 1 apresenta os Termos da linguagem especializada de enfermagem proveniente dos prontuários e achados clínicos constantes na CIPE® Versão 2019, segundo o Modelo dos Sete Eixos.

Quadro 1 – Termos da linguagem especializada de enfermagem proveniente dos prontuários e achados clínicos constantes na CIPE® Versão 2019, relevantes para a prática clínica direcionada ao RN em uso de PICC não constantes na CIPE® Versão 2019. Natal, RN, 2020.

**EIXO AÇÃO**<br>ação; acompanhar; administrar; agendar; ajustar; aliviar; alterar; analisar; aplicar; arranjar (organizar); atender; atender por meio de intervenção; atenuar; aumentar; auscultar; auxiliar; avaliar; calcular; categorizar; cobrir; colaborar; coletar; colocar ou pôr; completar; comprimir; conferir; confirmar (ou comprovar); contar; contatar; conter (ou limitar); controlar; coordenar; dar; demonstrar; descartar; descrever; desenvolver; desinfetar; determinar; determinar a intervenção; diminuir; distribuir; documentar;; encaminhar; estabelecer; estabilizar; estadiar; estar presente; esterilizar; estimular; evitar; examinar; executar; executar vigilância; facilitar; fazer rastreamento (*screening*); fazer triagem; friccionar; garantir (ou assegurar); gerenciar; guiar; higienizar (ou cuidar da higiene); identificar; imobilizar; implementar; induzir; iniciar; injetar; inserir; inspecionar; instalar; instilar; interpretar; interromper; isolar; lavar; lidar; liderar; limpar; manipular; manter; massagear; mediar; medir (ou verificar); minimizar; mobilizar; monitorar; notificar; observar; obter; obter dados; ordenar; ordenhar; organizar; orientar; otimizar; paliar; palpar; participar; permitir; planejar; posicionar; preparar; prescrever; prevenir; prevenir contaminação; priorizar; priorizar regime terapêutico; promover; proteger; prover (proporcionar, fornecer); puncionar; reforçar; registrar; regular; relatar; remover; requisitar (ou requerer); responder; restaurar; restringir (ou fazer contenção); sugar (ou aspirar); supervisionar; suprimir; suspender uso; testar; tocar; tornar (algo) maior ou melhor; traçar (ou rastrear); tranquilizar; transferir; transportar; tratar; treinar; trocar; vigiar (ou investigar).
**EIXO CLIENTE**<br>recém-nascido; criança; grupo; indivíduo; membro da família; paciente.
**EIXO FOCO**<br>acesso; acesso intravenoso (ou endovenoso); acidose metabólica; acidose respiratória; adaptação; agitação; alcalose metabólica; alcalose respiratória; alergia; alergia a medicação; amostra (ou espécimen); amostra (ou espécimen) de sangue arterial; amostra (ou espécimen) de sangue venoso [periférico, central, misto]; apneia; arritmia; atenção; atitude; atitude em relação ao cuidado; atividade psicomotora; atividade psicomotora, prejudicada; bradicardia; choque; choque cardiogênico; choque hipovolêmico; choque séptico; choque vasogênico; choro; clima; complicação; complicação associada à atenção à saúde; complicação, ausente; comportamento; comprimento; comunicação; condição; condição cardíaca; condição cardiovascular; condição de perfusão; hipoglicemia; hipomagnesemia; hiponatremia; hipopotassemia; hipotensão; hipotermia; hipóxia; infecção; infecção, ausente; inflamação; integridade tissular; condição endócrina; condição fisiológica; condição neurológica, prejudicada; condição nutricional; condição nutricional, positiva; condição nutricional, prejudicada; condição respiratória; condição social; condição, positiva; condição, prejudicada; confiança; confortável; conforto; confusão; confusão, ausente;

conjunto de processos corporais; consciência; consciência (ou cognição); contaminação; continuidade; continuidade do cuidado; controle; controle da dor; cuidar (ou tomar conta); desconforto; desequilíbrio ácido-base; desequilíbrio de eletrólitos; desequilíbrio de líquidos; desidratação; desidratação hipertônica; desidratação hipotônica; desidratação isotônica; dispneia; dor; dor abdominal; dor, ausente; edema; edema periférico; edema transudativo; efeito anestésico; efeito antibiótico; efeito colateral; efeito colateral da medicação; efeito de medicação; eliminação; energia; equilíbrio de líquidos (ou balanço hídrico); eritema; estresse; exame físico; fenômeno; fenômeno relativo a diagnóstico e resultado; frequência cardíaca; frequência de pulso; frequência de pulso radial; frequência respiratória; glicose sanguínea; graus de temperatura corporal; hematoma; hemorragia; hidratação, adequada; higiene das mãos; hiperbilirrubinemia; hipercalcemia; integridade da pele; integridade da pele, prejudicada; integridade tissular; integridade tissular, positiva; integridade tissular, prejudicada; interação medicamentosa, adversa;papel de enfermeira(o); pele, prejudicada; pele, úmida; perfusão tissular; perfusão tissular, periférica; personalidade; peso; política; política de saúde; pressão; pressão arterial [invasiva, não invasiva]; procedimento; processamento de informação; processo; processo cardíaco; processo cardíaco, eficaz; processo cardíaco, prejudicado; processo corporal prejudicado; processo do sistema circulatório; processo do sistema circulatório, positivo; processo do sistema circulatório, processo do sistema gastrointestinal; processo do sistema gastrointestinal, prejudicado; processo do sistema imune, eficaz; processo do sistema imunológico; processo do sistema imunológico, prejudicado; processo do sistema nervoso; processo do sistema regulatório, prejudicado; processo do sistema respiratório; processo do sistema respiratório, prejudicado; processo do sistema urinário; processo do sistema urinário, eficaz; processo do sistema urinário, prejudicado; processo intencional; processo neurovascular, prejudicado; processo patológico [sepse, insuficiência respiratória aguda, pneumonia, regime de nutrição enteral; regime de nutrição parenteral; regime medicamentoso; regime medicamentoso, complexo; regurgitação; resistência; respiração ofegante; responsividade; resposta à medicação; resposta à nutrição enteral; resposta à terapia; resposta à terapia com líquidos (ou hidratação); resposta à termorregulação; resposta ao tratamento; resposta ao tratamento, negativa; resultado de gasometria [arterial, venosa, venosa central, venosa mista]; resultado de teste; resultado laboratorial [coagulograma, contagem de bastões, contagem de leucócitos, creatinina, dosagem de eletrólitos, hemograma, níveis de plaquetas, ureia]; retenção de líquidos; retenção urinária; ritmo; ritmo cardíaco; ritmo respiratório; rotina; rotina de hora para dormir; sangramento; sangramento, ausente; sangue; saturação de oxigênio no sangue [arterial, venoso central, venoso misto]; saúde; secreção [espessa, mucopurulenta, purulenta, sanguinolenta, serosa]; sedação; sedação, eficaz; sinal; sinal de desconforto; sinal de infecção; sinal de pressão arterial; sinal vital; sinal vital de frequência cardíaca; sintoma; sintoma de infecção; sistema cardiovascular; sistema corporal; sistema endócrino; sistema gastrointestinal; sistema imunológico; sistema nervoso; sistema reprodutivo; sistema respiratório; sono; sono, adequado; sono, prejudicado; super-hidratação isotônica; super-hidratação isotônica; suprimento; suscetibilidade; suscetibilidade a infecção; suspeita; taquicardia; temperatura; temperatura corporal; tendência; termorregulação; teste diagnóstico; volume de líquidos, eficaz; volume de

líquidos, prejudicado; embolia; necrose.
**EIXO JULGAMENTO**<br>alto; anormal; atrasado (ou lento); baixo; completo; complexidade; complexo; dependência; deteriorado; eficaz; estado; estado de normalidade; estado de prescrição; estado julgado como relativo; extensão; grande; grau; gravidade; independência; iniciado; interrompido; julgamento, positivo ou negativo; leve; melhorado; moderado; nenhum; nível esperado; nível, absoluto; normal; parcial; pequeno; potencial para risco; potencialidade; prejudicado; prescrito; presença; progresso; real; risco; severo; simples; tamanho; tamanho médio.
**EIXO LOCALIZAÇÃO**<br>abdome [distendido, doloroso, flácido, globoso, tenso, timpânico]; antebraço; anterior; ânus; artéria [radial, braquial, femoral]; componente do sistema cardiovascular; componente do sistema corporal; componente do sistema imunológico; componente do sistema musculoesquelético; componente do sistema respiratório; conjuntiva; construção; coração; corpo; direita; distal; enfermaria; esquerda; estômago; membrana mucosa; membrana mucosa oral (ou bucal); músculo; pele; periférico; períneo; posição; posição corporal; posição prona (ou decúbito ventral); posição supina (ou decúbito dorsal); posição, anterior ou posterior; posição, central ou periférica; posição, esquerda ou direita; posição, inferior ou superior; posição, proximal ou distal; posição, unilateral ou bilateral; posterior; prepúcio; proximal; pulmão; região axilar; região corporal; região inferior do corpo; região umbilical; unidade de terapia intensiva; unidade diagnóstica; unilateral; universidade; vaso sanguíneo; veia; veia sistêmica; via intravenosa (ou endovenosa); via oral; via parenteral.
**EIXO MEIOS**<br>acesso venoso central (linha central); agulha; alarme; alarme de saída; alimento; ativa; analgésico; antibiótico; cânula venosa; cateter; compressas/gazes; dispositivo de segurança; dispositivo invasivo; dispositivo para acesso vascular;; dispositivo para cobertura [filme transparente]; dispositivo para infusão; dispositivo para monitoração; enfermeira(o); equipe interprofissional; equipo para soro; incubadora; instrumento de avaliação; material; medicação; medicamento comercial (de marca); monitor cardíaco; plano; plano de cuidado; prestador (ou provedor) de cuidados de saúde; prontuário do paciente; protocolo; serviço de enfermagem; serviço de saúde; solução [soro fisiológico, soro glicosado, soro ringertécnica; técnica asséptica; técnica de inserção invasiva; terapia com líquidos (ou hidratação); terapia de infusão; terapia intravenosa (ou endovenosa); ventilador; ventilador mecânico; técnica de posicionamento.
**EIXO TEMPO**<br>admissão; agudo; alta; amanhã; ano; contínuo; crônico; dia; duração; encontro; encontro (ou consulta); marcado; evento ou episódio; exame; frequência; hoje; hospitalização; idade gestacional; início; intermitente; manhã; noite; ontem; operação; passado; ponto no tempo ou intervalo de tempo; presente; semana; sempre; sequência no tempo; situação; tarde; parto (ou nascimento).

O quadro 2 apresenta os Termos da linguagem especializada de enfermagem provenientes dos prontuários clínicos e achados clínicos, relevantes para a prática clínica direcionada ao RN em uso de PICC não constantes na CIPE® Versão 2019.

Quadro 2 – Termos da linguagem especializada de enfermagem provenientes dos prontuários clínicos e achados clínicos, relevantes para a prática clínica direcionada ao RN em uso de PICC não constantes na CIPE® Versão 2019. Natal, RN, 2020.

**EIXO AÇÃO**<br>abordar; adotar; abranger; absorver; adequar; adquirir; ajudar; ambientar; aparecer; administrar Cateter Venoso Central de Inserção Periférica (CCIP); compreender; comprometer; estruturar; focar; imunizar; limpar; prestar; realizar; referenciar; requerer; verificar;
**EIXO CLIENTE**<br>cliente; paciente grave; paciente neonato; neonato.
**EIXO FOCO**<br>agente infeccioso; anemia; bradipneia; cabeceira; comorbidade; contagem de bastões; contagem de leucócitos; contagem de plaquetas; cuidado; disfunção orgânica; distúrbio de coagulação; diurese [ausente, espontânea, presente, reduzida]; dosagem de eletrólitos; equimose; estado geral [bom, comprometido, grave, regular]; estágio da sepse; evolução clínica; fator de risco; flebite; foco infeccioso; glicemia; hiperemia; parâmetros do ventilador mecânico; petéquias; prevenção; pressão arterial média; pulso [cheio, filiforme, fino, impalpável]; reposição; ruídos adventícios [crepto, estertor, ronco, sibilo]; ruídos hidroaéreos; saturação periférica de oxigênio; sepse; sinais de disfunção orgânica; sinais de hipoperfusão tecidual; taquipneia; variação da pressão de pulso; vazão de infusão.
**EIXO JULGAMENTO**<br>abundante; ausente; inaudível.
**EIXO LOCALIZAÇÃO**<br>laboratório; região abdominal; região cefálica; região torácica.
**EIXO MEIOS**<br>acesso arterial; antimicrobiano; antisséptico; bioquímica; bomba de infusão; Cateter Venoso Central de Inserção Periférica (CCIP); dieta; dissecção venosa; droga vasoativa; eletrocardiograma; escala de dor; escala de agitação; radiografia, ressonância nuclear magnética, ultrassom]; exame laboratorial; exame microbiológico de cultura [líquor, ponta de cateter, sangue]; gasometria arterial; gasometria venosa mista; guideline; isolamento de contato; imunização; remédio; resultado de culturas; vacinação; venóclise; ventilação mecânica [invasiva, não invasiva].
**EIXO TEMPO**<br>detecção precoce; extubação; horário.

O quadro 3 apresenta os estressores, os níveis dos estressores, diagnósticos de enfermagem da CIPE 2019 e suas definições operacionais em recém-nascidos com Cateter Venoso Central de Inserção Periférica.

Quadro 3. Estressores, níveis dos estressores, Diagnósticos de Enfermagem da CIPE 2019 e suas definições operacionais em recém-nascidos com Cateter Venoso Central de Inserção Periférica. Natal/RN, 2020.

**Estressores**	**Nível do estressor**	**Diagnóstico de Enfermagem**	**Definição do Diagnóstico**
Distúrbio de coagulação	Intrapessoal	Risco de Hemorragia	**Risco:**<br>Potencialidade: Que existe em possibilidade, risco.<br>**Hemorragia**:<br>Sangramento: Perda de grande quantidade de sangue, em um curto período de tempo, externa ou internamente, associada a sangramento arterial, venoso ou capilar.
Manuseio do paciente	Interpessoal	Agitação	**Agitação**:<br>Hiperatividade:<br>Circunstância de excitação psicomotora despropositada, atividade incansável, andar ritmado, liberação da tensão nervosa associada a ansiedade, medo ou estresse mental.
Punção endovenosa;<br>Troca do curativo.	Extrapessoal	Dor	**Dor:**<br>Percepção, Prejudicada: Aumento de sensação desagradável no corpo; relato subjetivo de sofrimento, expressão facial de dor, alteração no tônus muscular, comportamento autoprotetor, foco de atenção reduzido, alteração do tempo de percepção, afastamento de contato social, processo de pensamento prejudicado, comportamento distraído, inquietação e perda do

			apetite.
Exposição à microorganismos hospitalares	Extrapessoal	Risco de Infecção	**Risco:** Potencialidade: Que existe em possibilidade, risco. **Infecção**: Processo Patológico: Invasão do corpo por microrganismos patogênicos que se reproduzem e se multiplicam, originando doenças por lesão celular local, secreção de toxina ou reação antígeno-anticorpo.
		Infecção	**Infecção:** Processo Patológico: Invasão do corpo por microrganismos patogênicos que se reproduzem e se multiplicam, originando doenças por lesão celular local, secreção de toxina ou reação antígeno-anticorpo.
Exposição à baixa temperatura	Extrapessoal	Hipotermia	**Hipotermia**: Termorregulação, Prejudicada: Diminuição da capacidade para alterar o termostato interno, redução da temperatura corporal, frio, pele seca e pálida, tremores, preenchimento capilar lento, taquicardia, leito ungueal cianótico, hipertensão, piloereção associada a exposição prolongada ao frio, disfunção do sistema nervoso central ou do sistema endócrino sob condições de frio ou de indução artificial de uma temperatura corporal abaixo

			do normal por razões terapêuticas.
		Termorregulação, Prejudicada	**Termorregulação:**<br>Processo do Sistema Regulatório: Controle da produção e perda de calor por meio de mecanismos fisiológicos ativados pelo hipotálamo, pele e temperatura corporal.<br>**Termorregulação ineficaz**:<br>Oscilação da temperatura entre hipotermia e hipertermia.
		Bradicardia	**Bradicardia**:<br>Arritmia: Batimentos cardíacos lentos; frequência de pulso inferior a 60 batimentos por minuto em adultos.
Presença do dispositivo; Cateter posicionado em localização periférica; Quantidade elevada de soluções infundidas pelo cateter.	Extrapessoal	Edema	**Edema:**<br>Retenção de Líquidos.
		Hematoma	**Hematoma**:<br>Sangramento: Coleção e acúmulo de sangue retido nos tecidos, associados a trauma ou hemostasia incompleta após cirurgia; massa palpável, sensível ao toque; pele dolorida, com aspecto azul-esverdeado, desbotado ou amarelado.
		Integridade da Pele, Prejudicada	**Integridade:**<br>Condição: Ser íntegro, inteiro, não diminuído ou intacto.<br>**Pele**:<br>Componente do Sistema Tegumentar: Superfície natural, robusta e flexível, mais externa do corpo, com funções relacionadas com a elasticidade, a textura e a

			espessura, de modo a manter a queratinização da camada de revestimento intacta, hidratada, macia, nem extremamente fria, nem quente.<br>**Integridade da pele prejudicada**:<br>Epiderme e/ou derme alterada
		Trauma Vascular	**Trauma Vascular**:<br>Dano em veia e tecidos ao redor relacionado à presença de cateter e/ou soluções infundidas que pode comprometer a saúde.
		Integridade Tissular, Prejudicada	Dano em membrana mucosa, córnea, sistema tegumentar, fáscia muscular, músculo, tendão, osso, cartilagem, cápsula articular e/ou ligamento.
		Perfusão Tissular Periférica, Ineficaz	Redução da circulação sanguínea para a periferia que pode comprometer a saúde.
		Risco de Embolia	**Risco:**<br>Potencialidade: Que existe em possibilidade, risco.<br>**Embolia**:<br>Processo Vascular, Negativo.
		Risco de Necrose	**Risco:**<br>Potencialidade: Que existe em possibilidade, risco.<br>**Necrose:**<br>Ferida Traumática: Morte do tecido associada a inflamação local, processo infeccioso ou maligno ou lesão mecânica do tecido. Os níveis estão graduados, de acordo com a

			severidade, associada à duração da falta de oxigenação do tecido, desde pele esbranquiçada e pálida, acompanhada de dor intensa devido a afecção dos nervos superficiais, até necrose azul e preta da pele, perda de sensibilidade e dor, devido a lesões nervosas, com alto risco para infecções da ferida, perda de tecido danificado e partes do corpo.
		Função do Sistema Regulatório, Prejudicada	Sistema de regulação, controle.
Inserção do cateter	Extrapessoal	Sinal Vital (Sinais Vitais), Alterado	Sinais Vitais:<br>medidas corporais básicas de um ser humano.
		Risco de Função do Sistema Nervoso, Prejudicada	Sistema Nervoso:<br>conjunto formado por ligações de nervos e órgãos do corpo
		Risco de Função do Sistema Respiratório, Prejudicada	**Risco:**<br>Potencialidade: Que existe em possibilidade, risco.<br>Processo do Sistema Respiratório, Prejudicado.
		Risco de Perfusão Tissular, Ineficaz	**Risco:**<br>Potencialidade: Que existe em possibilidade, risco.<br>Perfusão Tissular, Ineficaz:<br>Redução da circulação sanguínea para a periferia que pode comprometer a saúde.
		Risco de Condição Neurológica, Prejudicada	**Risco:**<br>Potencialidade: Que existe em possibilidade, risco.<br>Condição Neurológica:

			Condições do sistema nervoso.
Localização do dispositivo	Extrapessoal	Risco de Função Cardíaca, Prejudicada	Processo Cardíaco, Prejudicado.
		Ritmo cardíaco prejudicado	Frequência/ritmo cardíaco alterado
Uso de luvas estéreis com Látex	Extrapessoal	Alergia a Látex	**Risco:**<br>Potencialidade: Que existe em possibilidade, risco.<br>Alergia: Resposta imunológica resultante do contato com produtos derivados de borracha.
Uso de antisséptico s	Extrapessoal	Alergia	Processo do Sistema Imune, Prejudicado: Resposta imunológica a um antígeno estranho.
Administraç ão de medicação	Extrapessoal	Risco de alergia a Medicação	**Risco:**<br>Potencialidade: Que existe em possibilidade, risco.
Presença do dispositivo; Eventos adversos relacionado s ao PICC.	Extrapessoal	Complicação Associada à Atenção à Saúde	Complicação Associada à Atenção à Saúde.
Presença de solução hiperosmola r;<br>Infusão de diferentes tipos de soluções.	Extrapessoal	Obstrução do Cateter	Obstrução: Processo.
		Medicação Adversa	Interação Medicamentosa, Adversa.
		Interação Medicamentosa, Adversa	Resposta à Medicação.

O quadro 4 apresenta os IVC dos Diagnósticos, Resultados e Intervenções de Enfermagem da CIPE 2019 em recém-nascidos com Cateter Venoso Central de Inserção Periférica.

Quadro 4 – IVC dos Diagnósticos, Resultados e Intervenções de Enfermagem da CIPE 2019 mais frequentes em recém-nascidos com Cateter Venoso Central de Inserção Periférica. Natal/RN, 2020.

**Diagnóstico de Enfermagem**	**Delph 1 (IVC)**	**Delph 2 (IVC)**	**Resultados de Enfermagem**	**Delph 1 (IVC)**	**Delph 2 (IVC)**	**Intervenções de Enfermagem**	**Delph 1 (IVC)**	**Delph 2 (IVC)**
Risco de Hemorragia	0,814	1,00	Controle de Risco de Hemorragia	0,808	1,00	Identificar Risco de Hemorragia<br>Monitorar Débito de Líquidos<br>Monitorar Equilíbrio de Líquidos (ou Balanço Hídrico)<br>Gerenciar Sangramento<br>Medir (ou verificar) Débito de Líquidos	0,895	1,00
Agitação	0,802	1,00	Agitação, Reduzida	0,802	1,00	Implementar Guia de Conduta para Dor<br>Monitorar Dor<br>Posicionar Paciente<br>Administrar Medicação para Dor<br>Analgesia Controlada por Enfermeira(o)<br>Avaliar Resposta à Medicação<br>Avaliar Resposta ao Manejo (Controle) da Dor	0,837	1,00
Dor	0,895	1,00	Controle da Dor	0,869	1,00	Implementar Guia de Conduta para Dor<br>Monitorar Dor<br>Posicionar Paciente<br>Administrar Medicação para	0,856	1,00

						Dor Analgesia Controlada por Enfermeira(o)<br>Avaliar Resposta à Medicação<br>Avaliar Resposta ao Manejo (Controle) da Dor		
Risco de Infecção	0,831	1,00	Controle de Risco de Infecção	0,802	1,00	Cuidados com Local de Dispositivo Invasivo<br>Monitorar Sinais e Sintomas de Infecção<br>Utilizar Técnica Asséptica<br>Prevenir Infecção<br>Obter Dados sobre Sinais e Sintomas de Infecção	0,843	1,00
Hipotermia	0,824	1,00	Controle da Hipotermia	0,844	1,00	Promover Termorregulação, Positiva<br>Obter Dados sobre Risco de Termorregulação, Negativa<br>Monitorar Temp. Corporal<br>Avaliar Resposta à Termorregulação	0,893	1,00
Termorregulação, Prejudicada	0,817	1,00	Termorregulação, Eficaz	0,843	1,00	Promover Termorregulação, Positiva<br>Obter Dados sobre Risco de Termorregulação, Negativa<br>Monitorar Temp. Corporal Avaliar Resposta à Termorregulação	0,826	1,00

Edema	0,806	1,00	Edema, Ausente	0,896	1,00	Cuidados com Local de Dispositivo Invasivo<br>Implementar Regime de Cuidados com a Pele<br>Manter Integridade da Pele<br>Monitorar Equilíbrio de Líquidos (ou Balanço Hídrico)<br>Monitorar Perfusão Tissular<br>Medir (ou Verificar) Débito de Líquidos	0,863	1,00
Hematoma	0,804	1,00	Hematoma, Ausente	0,885	1,00	Cuidados com Local de Dispositivo Invasivo<br>Implementar Regime de Cuidados com a Pele<br>Manter Integridade da Pele<br>Aplicar Compressa Fria	0,896	1,00
Integridade da Pele, Prejudicada	0,806	1,00	Integridade da Pele, Eficaz	0,821	1,00	Cuidados com Local de Dispositivo Invasivo<br>Implementar Regime de Cuidados com a Pele<br>Manter Integridade da Pele	0,803	1,00
Sinal Vital (Sinais Vitais), Alterado	0,817	1,00	Sinal Vital, no limite normal	0,832	1,00	Identificar condição fisiológica<br>Monitorar condição fisiológica<br>Monitorar Sinais Vitais	0,844	1,00

O quadro 5 apresenta a categorização das intervenções de Enfermagem da CIPE segundo as Intervenções de prevenção.

Quadro 5 – Intervenções de Enfermagem da CIPE 2019 em recém-nascidos com Cateter Venoso Central de Inserção Periférica segundo Intervenções de prevenção. Natal/RN, 2020.

**Intervenções da CIPE**	**Intervenções de Prevenção**
Educação em saúde<br>Cuidados com Local de Dispositivo Invasivo<br>Monitorar Pressão Arterial<br>Identificar Condição Fisiológica<br>Identificar Risco de Hemorragia<br>Implementar Guia de Conduta para Dor<br>Implementar Regime de Cuidados com a Pele<br>Manter Integridade da Pele<br>Medir (ou Verificar) Temperatura Corporal<br>Monitorar a Integridade da Pele<br>Monitorar Condição Fisiológica<br>Monitorar Débito de Líquidos<br>Monitorar Dor<br>Monitorar Equilíbrio de Líquidos (ou Balanço Hídrico)<br>Monitorar Sinais e Sintomas de Infecção<br>Monitorar Sinais Vitais<br>Utilizar Técnica Asséptica<br>Promover Termorregulação, Positiva<br>Obter Dados sobre Risco de Termorregulação, Negativa Monitorar Temperatura Corporal<br>Posicionar Paciente<br>Prevenir Infecção	Primária
Administrar Medicação para Dor<br>Analgesia Controlada por Enfermeira(o)<br>Aplicar Compressa Fria<br>Avaliar Resposta à Medicação<br>Avaliar Resposta à Termorregulação<br>Avaliar Resposta ao Manejo (Controle) da Dor<br>Avaliar Resposta ao Tratamento<br>Gerenciar Nutrição Parenteral<br>Gerenciar Sangramento<br>Monitorar Resposta ao Tratamento	Secundária
Avaliar Plano de Cuidados<br>Avaliar Regime Terapêutico	Terciária

Abaixo apresenta-se um diagrama dos estressores e estratégias de prevenção primária, secundária e terciária em recém-nascidos com Cateter Venoso Central de Inserção Periférica.

Figura 7 - Diagrama dos estressores e estratégias de prevenção primária, secundária e terciária em recém-nascidos com Cateter Venoso Central de Inserção Periférica. Natal, RN, Brasil, 2020.

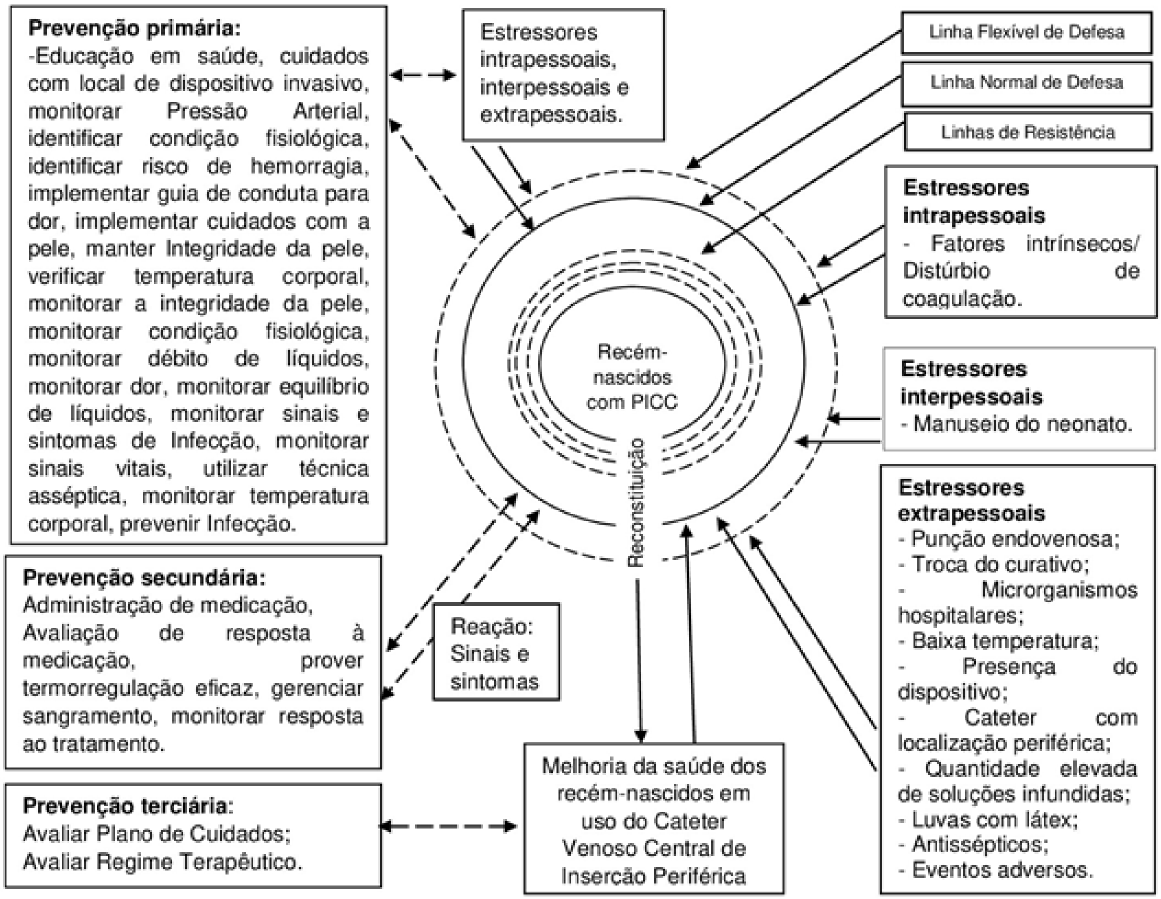

6. DISCUSSÃO

O advento das inovações tecnológicas na assistência neonatal intensiva interferiu profundamente na sobrevivência dos neonatos (ROSA; GAÍVA, 2009). Como dispositivo inovador na terapia endovenosa, o PICC é considerado uma opção extremamente benéfica aos recém-nascidos hospitalizados. Em estudo que analisou a influência das intervenções clínicas realizadas em UTI neonatal, verificou-se que o cateter epicutâneo reduz as chances de óbito neonatal na primeira semana de vida (BITTENCOURT; GAIVA, 2014).

Embora o uso de dispositivos venosos seja essencial no cuidado neonatal, o paciente em uso de cateter vascular está sujeito a stressores capazes de influenciar negativamente o seu bem-estar (BRAGA *et al*., 2018).

E nesse contexto, ao se refletir no cateter epicutâneo como dispositivo capaz de causar impactos negativos em nível fisiológico, psicológico e desenvolvimental, a aproximação da teoria de Betty Neuman para a abordagem do neonato com o PICC é válida e relevante.

Ao considerar o neonato como um ser composto por linhas de preservação que o protegem de estressores, o enfermeiro possui o papel fundamental de fortalecer as linhas de proteção do neonato, a fim de manter o equilíbrio e bem-estar do recém-nascido (NEUMAN; FAWCETT, 2011). Assim, a identificação dos estressores presentes nos neonatos em uso do PICC permite a elaboração de diagnósticos, resultados e intervenções de enfermagem voltados para essa população.

No presente estudo foram elencados e validados pelos peritos 32 DE, sendo discutidos os de maior observação na prática clínica. Entre os diagnósticos de enfermagem validados, especificamente considerando os estressores intrapessoais, tem-se o Risco de hemorragia. Como estressor de base para esse DE, tem-se os fatores intrínsecos ao paciente, como o distúrbio de coagulação.

Entre os distúrbios de coagulação, a trombocitopenia neonatal é uma anormalidade hematológica comum que ocorre em 20–35% de todos os recém-nascidos nas unidades de terapia intensiva neonatal (KASAP *et al*., 2020). Muitas são condições clínicas que cursam com trombocitopenia no período neonatal, dentre elas, destacam-se: prematuridade, restrição de crescimento intrauterino (RCIU), sepse, enterocolite, asfixia perinatal (AMUTHA; RAMESH; VINOTH, 2016). Em

recém-nascidos prematuros, a trombocitopenia ocorre através de três mecanismos fundamentais: os megacariócitos são menores e têm menos genomas, razão pela qual produzem menos plaquetas e precursores megacariocíticos, bem como produção inadequada de trombopoietina (CREMER *et al.,* 2016). Desse modo, a inserção do PICC pode ser dificultada em razão de distúrbios de coagulação, devido ao sangramento excessivo.

O sangramento durante a inserção do PICC esteve entre as principais intercorrências em estudo que analisou o cateter desde a sua indicação até a sua remoção (BAGGIO *et al.,* 2019). A hemorragia pode gerar eventos adversos em distintos níveis de gravidade. Esta situação clínica é comumente encontrada em pacientes hospitalizados e pode ser monitorada, na maioria dos casos, através de análises laboratoriais. Em estudo que descreveu a implantação de um indicador de qualidade assistencial relacionado ao diagnóstico de enfermagem de pacientes com alto risco de sangramento, com base nos resultados de exames laboratoriais, evidenciou que a implantação do indicador de qualidade assistencial aprimorou o processo de comunicação, monitoramento e cuidado de enfermagem a pacientes com risco de sangramento (LUCENA *et al*., 2019).

Como intervenções elencadas pela CIPE para o DE em tela, citam-se: identificar o risco de hemorragia, monitorar débito de líquidos; monitorar equilíbrio de líquidos (ou balanço hídrico), gerenciar o sangramento; medir (ou verificar) débito de líquidos (CIPE, 2019). Além disso, é necessário o enfermeiro ter conhecimento acerca das particularidades inerentes a anatomia, fisiologia da pele e do sistema venoso do recém-nascido (BAGGIO *et al*., 2019).

O manuseio do neonato prematuro por parte do profissional foi considerado o estressor interpessoal de base para a identificação do DE Agitação.

Diversas condições clínicas podem culminar com a agitação do paciente. No entanto, quando se refere a agitação em neonatos, a literatura aponta a hipoglicemia como principal causa (HALABY; STEINKRAUSS, 2012; HOUIN; ROZANCE, 2014).

Pouco se tem relatos de agitação em neonatos submetidos a procedimentos invasivos, como é o caso do PICC. Em estudo que elaborou enunciados de diagnósticos de enfermagem a partir da CIPE® para as etapas do crescimento e desenvolvimento de crianças, foi identificado o DE Agitação apenas para crianças na primeira infância (29 dias a dois anos) (DANTAS; GOMES; SILVA; NÓBREGA, 2016).

Nesse ínterim, destaca-se que o comportamento agitado não é uma particularidade de nenhuma condição patológica específica. Portanto, um raciocínio clínico com foco na identificação da causa de base é primordial para guiar de maneira apropriada a investigação diagnóstica e as possíveis intervenções terapêuticas (DEL-BEN *et al*., 2017).

O manuseio do recém-nascido prematuro hospitalizado pode interferir no equilíbrio do bem-estar do paciente e no surgimento de consequências no desenvolvimento neurocomportamental, como manifestações de estresse, dor, alterações fisiológicas e comportamentais, o que pode culminar com a agitação do RN e consequente dificuldade em sua adaptação ao meio exterior (MARTINS *et al*., 2015).

Bebês nascidos prematuramente aparentaram ser menos organizados que os bebês a termo. Nesse sentido, com o objetivo de um acolhimento humanizado ao RN, a Teoria do Desenvolvimento Síncrono-ativo proposto por Heidelise Als delineia caminhos para a observação do funcionamento cerebral do bebê, através da observação de seu comportamento. Esta teoria determina o limiar do bebê em relação ao estresse, sua capacidade de autorregulação e sua “real” disponibilidade de energia para procedimentos clínicos (ALS, 1982; MARTINS *et al*., 2015).

O manuseio do recém-nascido, quando não realizado de modo gentil, pode ser interpretado pelo córtex prematuro como estimulação álgica, e poderá interferir de forma negativa na organização neuropsicomotora do paciente (DE SOUSA *et al*., 2017). Assim, a realização de procedimentos invasivos em unidade neonatal, como é o caso do PICC, deve ser uma decisão individualizada e deve colaborar com o plano terapêutico do recém-nascido, de modo que os benefícios superem os riscos (FITZGERALD, 2015).

Em relação aos estressores extrapessoais, elencou-se a punção endovenosa, a troca de curativos, a exposição à microrganismos hospitalares, a exposição do RN à baixa temperatura, a presença do dispositivo no interior do vaso, o cateter mal posicionado e a quantidade elevada de soluções infundidas pelo cateter. Dessa forma, foram identificados os DE: Dor, Risco de Infecção, Hipotermia, Termorregulação Prejudicada, Edema, Hematoma, Integridade da Pele Prejudicada e Sinal Vital Alterado.

O evento doloroso é frequente em UTI Neonatal, sendo a dor um tema amplamente estudado (AYMAR et al., 2014; BRITTO et al., 2014; MOTTA; CUNHA,

2015; PRESTES et al., 2016; SPOSITO et al., 2017; CAMPOS, 2018; OLIVEIRA et al., 2019). Em estudo internacional com 101 recém-nascidos com idade gestacional entre 24 e 40 semanas, identificou a ocorrência de aproximadamente 8 procedimentos dolorosos ao dia (BRITTO et al., 2014). Nacionalmente, estudo com 150 neonatos apontou 6,6 procedimentos invasivos por dia (SPOSITO *et al.,* 2017). Entre os procedimentos considerados dolorosos e estressantes pelos profissionais de saúde de uma unidade de terapia intensiva neonatal, o PICC foi apontado por unanimidade entre os enfermeiros (MORAES; FREIRE, 2019).

Tendo em vista a incapacidade de expressar a dor, os neonatos podem apresentar alterações comportamentais e expressões faciais, como: choro, fronte saliente, olhos espremidos, movimentação excessiva de membros e rigidez torácica, bem como alterações fisiológicas como aumento da frequência cardíaca e do cortisol e diminuição da saturação de oxigênio (AYMAR *et al.,* 2014; MOTTA; CUNHA, 2015). Como consequências a longo prazo tem-se o atraso no crescimento e desenvolvimento neurológico, a alta ativação cortical e alterações no desenvolvimento cerebral, o temperamento da afetividade negativa, o déficit cognitivo e motor (OLIVEIRA et al., 2019).

Bebês prematuros são submetidos a procedimentos dolorosos durante um período de rápido desenvolvimento cerebral e programação de sistemas de estresse. Os sistemas sensoriais do RN prematuro são funcionalmente imaturos e as células específicas no sistema nervoso central de neonatos prematuros são particularmente vulneráveis à excitoxicidade, estresse oxidativo e inflamação (PEREIRA et al., 2013).

Em estudo realizado com crianças de 0 a 5 anos, o DE Dor também esteve presente (DANTAS; SILVA; NÓBREGA, 2018). Tendo em vista que o processo de agitação do RN em uso de PICC é decorrente do manuseio durante a inserção do cateter e a dor é provocada pela punção venosa, as estratégias traçadas para os DE Agitação e Dor foram: implementar guia de conduta para dor, monitorar dor, posicionar paciente, administrar medicação para dor, analgesia controlada por enfermeira(o), avaliar resposta à medicação, avaliar resposta ao manejo (controle) da dor (CIPE, 2019).

Nesse sentido, ressalta-se a terapêutica de manuseio mínimo, a qual enfatiza o agrupamento de procedimentos a serem realizados em um mesmo horário, a fim de evitar a manipulação excessiva do prematuro, proporcionando o seu sono e

repouso, além de reduzir o estresse (PEREIRA *et al.,* 2013). Além disso, faz-se necessária a adoção de boas práticas através de capacitações da equipe e a utilização de protocolos baseados em evidências científicas com vistas a prevenir a dor (COSTA e*t al*., 2019).

No que concerne ao Risco de Infecção do RN em uso de PICC, sabe-se que o cateter em estudo, apesar dos seus benefícios, representa portais potenciais para infecções localizadas e sistêmicas da corrente sanguínea (KHANNA et al., 2013).

Quando hospitalizada, a criança se torna mais vulnerável aos riscos de infecções, devido a procedimentos invasivos para tratamento terapêutico, como por exemplo as punções venosas (SILVA *et al*., 2014). Na população de recém-nascidos, o grau do risco aumenta em decorrência da fragilidade nas barreiras de proteção, como a pele e a mucosa, bem como do sistema imunológico (DINIZ; FIGUEIREDO, 2014).

O DE Risco de Infecção foi um dos diagnósticos mais frequentes em estudo nacional que validou diagnósticos, resultados e intervenções de enfermagem em crianças de 0 a 5 anos utilizando a CIPE (DANTAS; SILVA; NÓBREGA, 2017). O risco de infecção relacionada ao dispositivo venoso está associado, principalmente, à técnica de inserção, ao tipo de solução infundida, aos métodos de barreira de precaução para inserção e manipulação do cateter, ao tempo de permanência e ao tipo de cateter utilizado (KHANNA *et al.,* 2013).

Prevenir a ocorrência de infecções associadas ao uso de cateteres centrais, como o PICC, requer várias estratégias, desde o momento da inserção, incluindo educação e treinamento daqueles que inserem o cateter, como também o uso de precauções máximas de barreira estéril, o controle do volume infundido e a monitorização dos sinais vitais (BELL; O’GRADY, 2017; DANTAS; SILVA; NÓBREGA, 2017). Além dos citados, o cuidado com o local do dispositivo invasivo, a monitorização de sinais e sintomas de infecção foram intervenções da CIPE elencadas para o DE Risco de Infecção em RN com cateter epicutâneo.

Concernente aos DE Hipotermia e Termorregulação Prejudicada encontrados na presente pesquisa, estes estiveram como stressor à exposição à baixa temperatura.

A normotermia é definida como a temperatura entre 36,5 ºC e 37,5 ºC. É considerada hipotermia leve quando a temperatura se encontra entre 36,4ºC e 36 ºC, hipotermia moderada entre 35,9º C e 32ºC e hipotermia severa quando a

temperatura fica abaixo de 32ºC. A hipotermia é reconhecida como fator de risco independente para o mau prognóstico em RN (BRASIL, 2014).

As evidências atuais comprovam que existe associação entre hipotermia e aumento da mortalidade de recém-nascidos prematuros (SOARES *et al*., 2020). Quando a temperatura corporal do neonato está baixa favorece mudanças fisiológicas relacionadas ao aumento da necessidade de oxigênio e da resistência vascular periférica, ocasionando diminuição do débito cardíaco (WHO; 1997).

Quando se trata de prematuros, existe o fato de eles apresentarem baixa quantidade de tecido adiposo marrom, que é a substância responsável pela termogênese, e apresentarem atividade metabólica muito reduzida antes das 32 semanas de idade gestacional, dificultando a manutenção da temperatura do organismo (KNOBEL; HOLDITCH-DAVIS, 2010).

O Diagnóstico de Enfermagem Hipotermia esteve presente em 54,3% da amostra de estudo que determinou a acurácia dos indicadores clínicos dos diagnósticos de Enfermagem hipertermia e hipotermia em recém-nascidos. Os indicadores clínicos mais prevalentes foram: distresse respiratório, hipóxia, cianose nos leitos ungueais, temperatura abaixo dos parâmetros normais e letargia (BRAGA *et al*., 2014).

Foi demonstrado que em RN, principalmente nos prematuros, o procedimento de inserção do PICC pode levar a diminuição da temperatura corporal (GHYSELEN *et al*., 2014; ÇAGLAR; GÖZEN; İNCE, 2014).

Autores apontaram que um número considerável de crianças (75%) apresentou hipotermia após o procedimento de inserção do Cateter Venoso Central de Inserção Periférica em um estudo que avaliou 24 inserções do cateter (VALADÃO *et al*., 2019).

Faz-se necessária a presença de enfermagem especializada, voltada à prevenção da hipotermia (SOARES *et al.,* 2020). Para isso, pode ser utilizado tocas, colchões aquecidos, incubadora e umidificação (CDC, 2015).

Estudo recente quase-experimental com dois grupos demonstrou ainda que o uso da manta térmica favorece a manutenção da temperatura corporal do RN durante a instalação do PICC (VALADÃO *et al*., 2019). Estudo similar utilizou saco de poliuretano na prevenção de queda da temperatura corporal durante procedimentos de longa duração (GHYSELEN et al., 2014). Aliado a essas medidas, o presente estudo elencou a obtenção de dados sobre risco de termorregulação

negativa, a monitorização da temperatura corporal e a avaliação da resposta à termorregulação.

A Presença do dispositivo do cateter no leito venoso, o cateter posicionado em localização periférica e a quantidade elevada de soluções infundidas pelo dispositivo foram considerados estressores para o DE Edema, Hematoma e Integridade da Pele Prejudicada.

O edema constitui-se no sinal clínico mais frequente para identificação da infiltração e reporta-se a um tipo de trauma vascular proveniente de uma lesão nas camadas da veia e subsequente perfuração, resultando na infiltração de soluções ou medicamentos não vesicantes nos tecidos próximos à inserção do cateter venoso (BRAGA *et al.,* 2016).

Em estudo onde foram avaliados 259 cateteres, dos quais 86% dos recém-nascidos eram prematuros, foi identificado o edema de membro como complicações associadas ao uso do cateter (SWERTS *et al.,* 2019). Um coorte prospectivo conduzido em UTIN com neonatos submetidos à instalação de 524 cateteres centrais de inserção periférica cujo objetivo foi elaborar um escore de risco para remoção não eletiva do cateter central de inserção periférica em neonatos apontou o edema como uma das causas de remoção não eletiva do dispositivo (COSTA *et al*., 2015).

Uma revisão retrospectiva internacional que realizou análises de regressão logística de efeitos mistos univariada e multivariada para avaliar as associações entre variáveis preditoras potenciais para a remoção não seletiva do PICC com uma amostra de 918 crianças com idade gestacional menor que 45 semanas apontou o edema como um dos fatores presentes na retirada não programada do cateter. Contudo, a inserção do PICC nas extremidades superiores foi associada a um risco reduzido de desenvolver edema (OR 0,54, p = 0,03) (PET *et al*., 2020).

Neonatos com diagnóstico de prematuridade (idade gestacional no nascimento abaixo de 37 semanas) parecem possuir risco aumentado de desenvolvimento de edema como complicação relacionadas ao Cateter Venoso Central de Inserção Periférica (OR 2,15, p = 0,014). Além disso, devido o tamanho reduzido dos vasos, recém-nascidos prematuros podem ter aumento do risco de edema com o uso do PICC (PET *et al.,* 2020).

A CIPE elenca importantes intervenções para prevenção e tratamento do edema, tais como: implementar cuidados com local de dispositivo invasivo,

implementar regime de cuidados com a pele, manter a integridade da pele e monitorar a infusão de líquidos.

Outro DE identificado foi o Hematoma, o qual é caracterizado pelo acúmulo de sangue retido nos tecidos e está associado ao processo vascular prejudicado, ou seja, associado a lesão de vasos sanguíneos pela punção venosa onde ocorre a transfixação do vaso com extravasamento de sangue (CIPE, 2019).

O hematoma é uma das principais complicações locais associadas aos acessos venosos, e sua ocorrência pode ocasionar consequências para o paciente e para o sistema de saúde, como, elevar o custo do tratamento, prolongar o tempo de hospitalização, aumentar a morbidade e, em casos mais graves, levar a um desfecho letal (DANSKI et al., 2015; GORSKI *et al*., 2016).

Apesar de não ter sido associado à remoção do PICC no presente estudo, o hematoma foi um evento com importante prevalência. Em estudo que objetivou identificar as práticas e intervenções de enfermagem relacionadas à terapia intravenosa em recém-nascidos e crianças, apontou o hematoma como complicação associada à remoção de cateteres venosos (NAKANDAKARI et al., 2018).

Nesta ótica, medidas que reduzam o risco deste DE devem ser implementadas, principalmente em populações vulneráveis como crianças e RN gravemente enfermo.

No presente estudo também foi identificado o diagnóstico Integridade da pele prejudicada, que ocorre quando a epiderme e/ou a derme sofre dano em decorrência da perfuração ou fricção da pele, umidade, alterações no turgor e/ou na pigmentação da pele, circulação prejudicada, eritema e lesões na pele (DANTAS; SILVA; NÓBREGA, 2017).

A pele do neonato é reconhecida como sensível, tênue e frágil. Ao associar estas propriedades com a imaturidade dos sistemas e a utilização de dispositivos hospitalares, necessários à sobrevivência desta população, ocorre um aumento considerável do risco de lesão da pele. Somado a isso, a utilização de películas, diretamente na pele, para fixação de dispositivos médicos, causa prejuízo à integridade da pele (MARTINS; CURADO, 2017).

Uma pesquisa qualitativa desenvolvida em Unidade de Terapia Intensiva Neonatal verificou que o DE Integridade da Pele Prejudicada foi um dos principais DE citados por enfermeiros que atuam na assistência ao RN de alto risco (BATISTA et al., 2019).

Nessa perspectiva, a CIPE apresenta importantes estratégias para o RN com o DE Integridade da pele prejudicada: manter os cuidados com local de dispositivo invasivo, implementar regime de cuidados com a pele e a busca constante da manutenção da integridade da pele.

Outro DE identificado na presente pesquisa foi Sinal Vital Alterado e a inserção do PICC foi o estressor de base.

Fatores estressantes como a realização de procedimentos invasivos, individualmente ou associados à sensação de dor, podem causar a desorganização de diferentes sistemas, contribuindo, portanto, para alterações nos parâmetros fisiológicos de neonatos hospitalizados (CORREIA; DE MENDONÇA; DE SOUZA, 2014; ORSI *et al*., 2015).

Estudos recentes apontam que punção venosa é possivelmente o procedimento que mais causa estresse nos neonatos (BARROS; LUIZ; MATHIAS, 2019; REBELATO; STUMM, 2019).

Durante a situação de estresse, o neonato é submetido a alterações na pressão arterial, frequência cardíaca (FC), frequência respiratória (FR) e saturação periférica de oxigênio (SpO2). Além disso, ocorre a liberação de indicadores hormonais de estresse, como cortisol, adrenalina e noradrenalina (CORDEIRO; COSTA, 2014; DIAS; MARBA, 2014).

A utilização de referenciais teóricos que visem a multidimensionalidade do indivíduo e que o veja como um sistema aberto que interage com seu meio, é capaz de auxiliar na relação com os cuidados de saúde, com ênfase na promoção do bem estar do indivíduo e na prevenção de comorbidades (NEUMAN; FAWCETT, 2011). Essa prevenção deve ser enfatizada na área da enfermagem, visto que estes profissionais estão em constante contato no cuidado e manutenção destes dispositivos invasivos, seja na sua inserção, vigia e observações quanto aos sinais que possam interferir na sua eficácia (SOUZA et al., 2020; GOMES; MENDES; PEDRO, 2020).

Portanto, se faz necessário, basear a prática dos cuidados de enfermagem, nos três níveis de prevenção, previstos na utilização da teoria dos sistemas: prevenção primária, prevenção secundária, e prevenção terciária. As intervenções nessas dimensões, visam auxiliar o enfermo a preservar, atingir e realizar a manutenção da homeostase (NEUMAN; FAWCETT, 2011).

As intervenções a nível primário são baseadas na prevenção do estresse, são realizadas antes do sistema reagir ao estressor. Essas ações podem ser iniciadas quando há um estressor conhecido, mas que ainda não alterou o bem estar do cliente. A primeira dimensão dessa prevenção é baseada na retenção do bem estar, fortalecendo a linha flexível de defesa natural do indivíduo, ou atuando na diminuição dos fatores de risco. O enfermeiro vai agir na redução da possibilidade do encontro com esse estressor, ou fortalecendo a linha de defesa do cliente, em conjunto da criação de estratégias de promoção à saúde. Em resumo, o objetivo das intervenções da prevenção primária, é promover o bem estar pela prevenção do estresse (BRAGA et al., 2018; NEUMAN; FAWCETT, 2011).

Nesse nível de intervenção, os diagnósticos de enfermagem de risco são predominantes, pois existe uma possibilidade de contato com um estressor.

Consoante a isso, é fundamental o conhecimento dos fatores de risco pelos profissionais que atuam nesse cuidado para atuação nessa prevenção. Alguns fatores de risco presentes na utilização do PICC são: as alterações na integridade da pele que podem ocorrer no momento de inserção do cateter até o seu destino final, além do comprometimento da barreira de defesa do paciente, também pode-se citar como fator de risco, alterações já presentes no indivíduo, como baixa maturidade imunitária, no casos dos RN, como também o conhecimento ineficiente, o manuseio inadequado ou a má higienização da mãos, por parte dos profissionais (BRAGA *et al*., 2018).

Para o nível de prevenção primária tem-se a educação continuada dos profissionais, os cuidados com o local do dispositivo invasivo, a identificação de riscos inerentes às condições fisiológicas do paciente, a implementação de guia para condutas frente ao manejo da dor, a observação contínua da pele do neonato e a manutenção de sua integridade, a promoção da termorregulação eficaz do recém-nascido prematuro, a utilização de técnica asséptica desde a inserção do dispositivo até o momento de sua remoção (CIPE; 2019).

É importante, também, que os profissionais tenham conhecimento sobre as práticas adequadas no manejo do PICC, possibilitando sua atuação na retenção do bem estar. Por exemplo, a monitorização frequente, realizada diariamente em conjunto com a investigação de sinais inflamatórios que possam estar presentes no óstio de inserção. Para auxiliar nesse processo, o uso de coberturas transparentes facilita a monitorização desse curativo. Estudos demonstram que para a melhoria da

adesão de boas práticas no âmbito hospitalar, há uma necessidade de criar *guidelines*, além da realização de trabalhos expositivos e de conscientização permanente sobre o assunto (RUSSO et al.,2020; GOMES; MENDES; PEDRO, 2020).

Quando as intervenções de nível primário não são realizadas ou são falhas, e consequentemente ocorre uma reação entre o estressor e o sistema, as intervenções de prevenção secundárias são realizadas. Nessa dimensão, os cuidados são voltados para a resolução ou diminuição das complicações. Portanto, o nível secundário de intervenção é realizado em resposta a sintomatologia. Esse nível de prevenção requer uma adequada priorização de intervenções. O objetivo do cuidado de enfermagem é reduzir os efeitos nocivos que serão gerados pelo estressor, além de fornecer tratamento com intuito de restabelecer o estado ideal do sistema, reabilitar o bem estar do cliente e fortalecer as linhas internas de resistência desse indivíduo (BRAGA et al., 2020; NEUMANN; FAWCETT, 2011).

Nesse nível, os cuidados de enfermagem visam a redução dos efeitos deletérios diante da reação ao stressor (NEUMAN; FAWCETT, 2011). Isso acontece, por exemplo, quando o enfermeiro administra medicamentos para o alívio da dor, realiza o gerenciamento das soluções infundidas, obtém dados sobre sinais e sintomas de infecção e monitora a resposta ao tratamento.

A remoção do cateter de forma não eletiva, se dá em resposta a possíveis problemas encontrados durante a avaliação do estado do cateter, como por exemplo a obstrução do sistema, ruptura do cateter, extravasamento do conteúdo infundido, flebite mecânica, exteriorização do cateter, presença de sinais de infeção e manuseio inadequado do curativo (BRAGA et al., 2018; PERES et al., 2019). A remoção do cateter em resposta a esses sintomas é uma forma de intervenção de nível secundário, e essa mediação tem o intuito de não permitir a evolução dos sintomas e consequentemente atingir as linhas de resistência e desenvolver uma maior gravidade no indivíduo (GOMES, MENDES; PEDRO; 2020; NEUMANN; FAWCETT, 2011).

A intervenção de nível terciário de prevenção se dá após o tratamento do sistema na prevenção secundária. Essa etapa é referente aos processos de ajuste que o sistema produz para sua reconstituição. Tal processo depende da boa sucessão dos recursos do cliente, e sua adaptação, para evitar futuras reações no sistema. Esse nível tem como objetivo dar continuidade às intervenções iniciadas no

nível secundário fortalecendo a resistência aos estressores e prevenindo a evolução de sinais e sintomas que podem interferir novamente na estabilidade do sistema. Outro componente dessa prática se dá pela atividade de educação visando prevenir a recorrência dessas perturbações do bem estar do indivíduo (BRAGA et al., 2020; NEUMAN; FAWCETT, 2011).

No terceiro nível de intervenção o paciente pode ser reavaliado quanto a indicação da recolocação do PICC. Além da atuação do controle institucional dos indicadores de qualidade no uso do cateter e promoção de ações educativas de acordo com a demanda encontrada (BRAGA, et al., 2020; GOMES, et al., 2019). Esses fatores de manutenção do bem estar do indivíduo o leva novamente, de forma circular ao início da prevenção, nas intervenções primárias. Ressalta-se que a avaliação constante do Plano de cuidados é etapa fundamental no cuidado ao RN com cateter PICC.

Como a teoria de Neumann é centrada na estabilização do bem estar, as ações de promoção à saúde, das intervenções primárias devem ser utilizadas em conjunto com a prevenção secundária e terciária, prevenindo complicações e restabelecendo o bem estar ideal do indivíduo (NEUMANN; FAWCETT, 2011).

7. CONCLUSÃO

O presente estudo permitiu a construção de uma nomenclatura de Diagnósticos, Resultados e Intervenções de enfermagem para recém-nascido com PICC utilizando a CIPE® validados por especialistas.

Foram elencados e validados 31 diagnósticos/resultados de enfermagem e foram propostas 154 intervenções de enfermagem. Os principais diagnósticos foram: Risco de Hemorragia, Agitação, Dor, Risco de Infecção, Hipotermia, Edema, Hematoma, Integridade da Pele prejudicada, Sinal Vital alterado.

A utilização do modelo de sistemas de Neuman no cuidado de enfermagem a neonatos em uso de PICC configurou um alicerce conceitual-filosófico que proporcionou uma releitura dessa prática na perspectiva de direcionar a avaliação clínica para a identificação dos stressores e dos DE.

Os estressores intrapessoais estiveram ligados aos fatores intrínsecos do paciente, como o distúrbio de coagulação. Os estressores interpessoais estiveram relacionados ao manuseio do RN e os principais estressores extrapessoais foram a punção endovenosa, as trocas do curativo; à exposição a microrganismos hospitalares, à exposição a baixas temperaturas.

As intervenções a nível primário dizem respeito às ações de educação continuada para os profissionais, aos cuidados com o local do dispositivo invasivo, a identificação de riscos inerentes às condições fisiológicas do paciente, a implementação de guias de condutas, o monitoramento contínuo da pele e a manutenção de sua integridade. As intervenções a nível secundário incluem a administração de medicamentos para o alívio da dor, o gerenciamento das soluções infundidas, além do monitoramento das respostas ao tratamento. A prevenção terciária faz alusão à avaliação do plano de cuidados.

As limitações da pesquisa se referem ao fato que os diagnósticos e as intervenções de enfermagem identificados se aplicam a realidade dos recém-nascidos hospitalizadas na UTIN do referido hospital do estudo. Assim, recomenda-se a aplicação de estudos com neonatos em outros cenários assistenciais.

Na prática clínica de enfermagem, os resultados deste estudo devem facilitar a inferência diagnóstica e o desenvolvimento de intervenções que possam promover resultados de enfermagem positivos e sensíveis. Assim, esses achados podem orientar os enfermeiros quanto à prestação de cuidados com melhor direcionamento

das intervenções de enfermagem e aumentar a clareza na mensuração dos resultados.

Os diagnósticos de enfermagem proporcionam aos enfermeiros que atuam em unidades neonatais a oportunidade de um gerenciamento eficaz do processo assistencial, bem como a disponibilização dos dados necessários para a melhoria contínua de uma assistência de enfermagem de qualidade.

REFERÊNCIAS

ALS, Heidelise. Toward a synactive theory of development: promise for the assessment and support of infant individuality. **Infant Mental Health Journal**, [Internet], v. 3, n. 4, p. 229-243, dez. 1982. Wiley. Disponível em: http://dx.doi.org/10.1002/1097-0355(198224)3:43.0.co;2-h. Acesso em: 06 out. 2020.

ALVES, Albertisa Rodrigues; LOPES, Consuelo Helena Aires de Freitas; JORGE, Maria Salete Bessa. Significado do processo de enfermagem para enfermeiros de uma unidade de terapia intensiva: uma abordagem interacionista. **Rev. Esc. Enferm. USP**, [Internet], v. 42, n. 4, p. 649-655, dez. 2008. FapUNIFESP (SciELO). http://dx.doi.org/10.1590/s0080- 62342008000400006. Disponível em: https://www.scielo.br/pdf/reeusp/v42n4/v42n4a05.pdf. Acesso em: 01 ago. 2020.

AMUTHA, J.; RAMESH, S.; VINOTH, S. Study of neonatal thrombocytopenia etiology, clinical profile, immediate outcome and short term follow up of neonatal thrombocytopenia. International **Journal of Current Medical And Pharmaceutical Research** [Internet], v. 2, n. 10, p. 828-30, oct. 2016. Disponível em: http://journalcmpr.com/sites/default/files/issue-files/CMPR-A- 0317.pdf. Acesso em: 01 out 2020.

AYMAR, Carmen L.G. de et al. Pain assessment and management in the NICU: analysis of an educational intervention for health professionals. **J Pediatr**, [Internet], v. 90, n. 3, p. 308-315, maio 2014. Elsevier BV. http://dx.doi.org/10.1016/j.jped.2013.09.008. Disponível em: https://www.scielo.br/pdf/jped/v90n2/pt_0021-7557-jped-90-03-00308.pdf. Acesso em: 14 ago. 2020.

BAGGIO, Maria Aparecida et al. Utilização do cateter central de inserção periférica em neonatos: análise da indicação à remoção. **Rev Rene**, [Internet], v. 20, p. 01-08, 25 set. 2019. Rev Rene - Revista da Rede de Enfermagem de Nordeste. http://dx.doi.org/10.15253/2175-6783.20192041279. Disponível em: http://repositorio.ufc.br/bitstream/riufc/46197/1/2019_art_mabaggio.pdf. Acesso em: 20 out. 2020.

BAGGIO, Maria Aparecida; BAZZI, Fernanda Cardoso da Silva; BILIBIO, Cassia Alcionara Conte. Cateter central de inserção periférica: descrição da utilização em UTI Neonatal e Pediátrica. **Rev. Gaúcha Enferm**. [Internet], Porto Alegre, v. 31, n. 1, p. 70-76, Mar. 2010. https://doi.org/10.1590/S1983-14472010000100010.Disponível em: http://www.scielo.br/scielo.php?script=sci_arttext&pid=S1983-14472010000100010&lng=en&nrm=iso. Acesso em: 01 set. 2020.

BARROS, Marcela Milrea Araújo; LUIZ, Bruna Viana Scheffer; MATHIAS, Claice Vieira. Pain as the fifth vital sign: nurses practices and challenges in a neonatal intensive unit care. **BrJP**, [Internet], v. 2, n. 3, p. 232-236, 2019. GN1 Genesis Network. http://dx.doi.org/10.5935/2595-0118.20190041. Disponível em:

https://www.scielo.br/pdf/brjp/v2n3/pt_2595-0118-brjp-02-03-0232.pdf. Acesso em: 12 out. 2020.

BASTOS, João Luiz Dornelles; DUQUIA, Rodrigo Pereira. Um dos delineamentos mais empregados em epidemiologia: estudo transversal. **Scientia Medica**, Porto Alegre, v. 17, n. 4, p. 229-232, out./dez. 2007. Disponível em: https://pdfs.semanticscholar.org/7577/438f4f985e091af06e96848ae3031f9c0206.pdf. Acesso em: 15 ago. 2020.

BATISTA, Camila Daiana Moraes et al. Diagnósticos e cuidados de enfermagem ao recém-nascido prematuro em Unidade de Terapia Intensiva Neonatal. **REAS/EJCH**, [Internet], n. 35, p. 01-06, 1 nov. 2019. http://dx.doi.org/10.25248/reas.e1593.2019. Disponível em: https://acervomais.com.br/index.php/saude/article/view/1593/941. Acesso em: 02 nov. 2020.

BELL, Taison; O'GRADY, Naomi P. Prevention of Central Line–Associated Bloodstream Infections. **Infect Dis Clin North Am**., [Internet], v. 31, n. 3, p. 551-559, set. 2017. Elsevier BV. http://dx.doi.org/10.1016/j.idc.2017.05.007. Disponível em: https://pubmed.ncbi.nlm.nih.gov/28687213/. Acesso em: 08 out. 2020.

BITTENCOURT, Rossana Marchese; GAIVA, Maria Aparecida Munhoz. Mortalidade neonatal precoce relacionada a intervenções clínicas. **Rev. bras. enferm**., Brasília, v. 67, n. 2, p. 195-201, Abr. 2014. http://dx.doi.org/10.5935/0034-7167.20140025. Disponível em: http://www.scielo.br/scielo.php?script=sci_arttext&pid=S0034-71672014000200195&lng=en&nrm=iso. Acesso em: 01 out. 2020.

BRAGA, Fernanda Cavalcante et al. Accuracy of clinical indicators of Nursing diagnoses hyperthermia and hypothermia in newborns. Rev Rene, [Internet], v. 15, n. 5, p. 789-795, 21 out. 2014. **Rev Rene**. http://dx.doi.org/10.15253/2175-6783.2014000500008. Disponível em: http://www.periodicos.ufc.br/index.php/rene/article/viewFile/3243/2498. Acesso em: 14 ago. 2020.

BRAGA, Luciene Muniz et al. Adaptação transcultural da Infiltration Scale para o português. **Acta Paul. Enferm**., [Internet], v. 29, n. 1, p. 93-99, fev. 2016. FapUNIFESP (SciELO). http://dx.doi.org/10.1590/1982-0194201600013. Disponível em: https://www.scielo.br/scielo.php?pid=S0103-21002016000100093&script=sci_arttext&tlng=en. Acesso em: 08 ago. 2020.

BRAGA, Luciene Muniz et al. El modelo de Betty Neuman en el cuidado al paciente con catéter venoso periférico. **Rev. Enf. Ref**., Coimbra, v. Ser IV, n. 19, p. 159-167, dez. 2018. http://dx.doi.org/10.12707/RIV18029. Disponível em: http://www.scielo.mec.pt/scielo.php?script=sci_arttext&pid=S0874-02832018000400017&lng=pt&nrm=iso. Acessos em: 01 out. 2020.

BRASIL. Conselho Nacional de Saúde. Resolução nº 466 de 12 de dezembro de 2012. Aprova diretrizes e normas regulamentadoras de pesquisas envolvendo seres humanos. Disponível em:

https://bvsms.saude.gov.br/bvs/saudelegis/cns/2013/res0466_12_12_2012.html. Acesso em 20 ago. 2020.

BRASIL. Ministério da Saúde. Secretaria de Atenção à Saúde. Departamento de Ações Programáticas Estratégicas. Atenção à saúde do recém-nascido: guia para os profissionais de saúde / Ministério da Saúde, Secretaria de Atenção à Saúde, Departamento de Ações Programáticas Estratégicas. – 2. ed. atual. – Brasília: Ministério da Saúde, 2014.

BRITTO, C. D. et al. PAIN--Perception and Assessment of Painful Procedures in the NICU. **Journal Of Tropical Pediatrics**, [Internet], v. 60, n. 6, p. 422-427, 21 jul. 2014. Oxford University Press (OUP). http://dx.doi.org/10.1093/tropej/fmu039. Disponível em: https://academic.oup.com/tropej/article/60/6/422/1681865. Acesso em: 22 ago. 2020.

CAMPOS, Ana Paula Silva. Dor neonatal: conhecimento, atitude e prática da equipe de enfermagem. **Br J Pain**., [Internet], v.1, n.4, p.354-8, out-dez 2018. Disponível em: https://www.scielo.br/pdf/brjp/v1n4/pt_1806-0013-brjp-01-04-0354.pdf. Acesso em: 12 ago. 2020.

CARVALHO, Carina Maris Gaspar; CUBAS, Marcia Regina; NÓBREGA, Maria Miriam Lima da. Brazilian method for the development terminological subsets of ICNP®: limits and potentialities. **Rev. Bras. Enferm**., [Internet], v. 70, n. 2, p. 430-435, abr. 2017. FapUNIFESP (SciELO). http://dx.doi.org/10.1590/0034-7167-2016-0308. Disponível em: https://www.scielo.br/scielo.php?script=sci_arttext&pid=S0034-71672017000200430. Acesso em 24 jun. 2020.

CENTERS FOR DISEASE CONTROL AND PREVENTION. **The National Healthcare Safety Network (NHSN) manual: patient safety component protocol**. Atlanta: CDC, 2015.

COFEN-Conselho Federal de Enfermagem, Parecer COFEN 243/2017. **Atualiza a normatização do procedimento de inserção, fixação, manutenção e retirada de cateter periférico central por Enfermeiro – PICC.** Disponível em: http://www.cofen.gov.br/parecer-de-relator-cofen-no-2432017_57604.html. Acesso em: 12 jun. 2020.

COFEN-Conselho Federal de Enfermagem. Resolução COFEN 258/2001. **Inserção de cateter periférico central pelos enfermeiros**. Disponível em: http://site.portalcofen.gov.br/node/4296. Acesso em: 12 jun. 2020.

COFEN - Conselho Federal de Enfermagem (BR). Resolução n. 348/2009. **Dispõe sobre a Sistematização da Assistência de Enfermagem e a implementação do Processo de Enfermagem em ambientes, públicos ou privados, em que ocorre o cuidado profissional de Enfermagem, e dá outras providências**. Brasília (DF): COFEN; 2009. http://www.cofen.gov.br/resoluo-cofen-3582009_4384.html. Acesso em: 15 jun. 2020.

COFEN - Conselho Federal de Enfermagem. Resolução n.º 311/2007, de 08 de fevereiro de 2007. **Dispõe sobre o Código de Ética dos Profissionais de**

Enfermagem. [resolução na internet]. Diário Oficial da União 2007. Disponível em: http://site.portalcofen.gov.br/node/4345. Acesso em: 01 ago. 2020.

COFEN - Conselho Federal de Enfermagem. Resolução n.º 358/2009, de 15 de outubro de 2009. **Dispõe sobre a Sistematização da Assistência de Enfermagem e a implementação do Processo de enfermagem em ambientes, públicos ou privados, em que ocorre o cuidado profissional de Enfermagem, e dá outras providências**. [resolução na internet]. Diário Oficial da União 2009. Disponível em: http://www.portalcofen.gov.br/sitenovo/node/4384. Acesso em: 01 ago. 2020.

COFEN - Conselho Federal de Enfermagem. Resolução nº. 272/2002, de 27 de agosto de 2002. **Dispõe sobre a sistematização da assistência de enfermagem – SAE- nas instituições de saúde brasileiras**. [resolução na internet]. Diário Oficial da União 27 ago 2002. Disponível em: http://www.coren-df.org.br/portal/index.php?option=com_content&view=article&id=98:resolucao- cofen-2722002&catid=36:resolucaes&Itemid=43. Acesso em: 01 ago. 2020.

Conselho Internacional de Enfermeiros. **Cipe Versão 1 - Classificação Internacional para a Prática de Enfermagem Versão 1.0**. São Paulo: Algol Editora; 2007. [Título original: International Classification for Nursing Practice – ICNP® Version 1.0].

Conselho Internacional de Enfermeiros**. Classificação Internacional para a Prática de Enfermagem Versão 1.0**. Lisboa, Portugal: Ordem dos Enfermeiros de Portugal; 2005. [Título original: International Classification for Nursing Practice – ICNP® Version 1.0]

CORDEIRO, Raquel Alves; COSTA, Roberta. Métodos não farmacológicos para alívio do desconforto e da dor no recém-nascido: uma construção coletiva da enfermagem. **Texto Contexto Enferm**., [Internet], Florianópolis, v.23, n.1, p. 185-92, 2014. Disponível em: https://www.scielo.br/pdf/tce/v23n1/pt_0104-0707-tce-23-01-00185.pdf. Acesso em: 11 out. 2020.

CORREIA, Cintia Quele de Oliveira Soares; DE MENDONÇA, Ana Elza Oliveira; DE SOUZA, Nilba Lima. Scientific production on noise in the neonatal intensive care unit: integrative review. **J Nurs UFPE online**., [Internet], Recife, v. 8(Suppl. 1):2406-12, July., 2014. http://dx.doi.org/10.5205/reuol.5927-50900- 1-SM.0807suppl201428. Disponível em: https://www.semanticscholar.org/paper/Scientific-production-on-noise-in-the- neonatal-care-Correia-Mendon%C3%A7a/2559eb2b8a84ba8e2628402b3bf5bbe2c94bac82?p2df. Acesso em: 11 out. 2020.

COSTA, Anna Caroline Leite et al. CORRELATIONAL ANALYSIS BETWEEN POTENTIALLY PAINFUL PROCEDURES AND PAIN CONTROL STRATEGIES IN A NEONATAL UNIT. **Texto Contexto - Enferm**., [Internet], v. 28, p. 01-12, 2019. FapUNIFESP (SciELO). http://dx.doi.org/10.1590/1980-265x-tce-2018- 0299. Disponível em: https://www.scielo.br/scielo.php?script=sci_arttext&pid=S0104-07072019000100370&lng=en&nrm=iso. Acesso em: 07 out. 2020.

COSTA, Priscila et al. Elaboração de um escore de risco para remoção não eletiva do cateter central de inserção periférica em neonatos. **Rev. Latino-Am. Enfermagem**, Ribeirão Preto, v. 23, n. 3, p. 475-482, 2015. https://doi.org/10.1590/0104-1169.0491.2578. Disponível em: http://www.scielo.br/scielo.php?script=sci_arttext&pid=S0104-11692015000300475&lng=en&nrm=iso. Acesso em 14 out 2020.

CREMER, Malte et al. Thrombocytopenia and platelet transfusion in the neonate. **Semin Fetal Neonatal Med**., [Internet], v. 21, n. 1, p. 10-18, fev. 2016. Elsevier BV. http://dx.doi.org/10.1016/j.siny.2015.11.001. Disponível em: https://pubmed.ncbi.nlm.nih.gov/26712568/. Acesso em: 02 out. 2020.

ÇAGLAR, Seda; GÖZEN, Duygu; İNCE, Zeynep. Heat Loss Prevention (Help) After Birth in Preterm Infants Using Vinyl Isolation Bag or Polyethylene Wrap. **J Obstet Gynecol Neonatal Nurs**., [Internet], v. 43, n. 2, p. 216-223, mar. 2014. Elsevier BV. http://dx.doi.org/10.1111/1552-6909.12291. Disponível em: https://linkinghub.elsevier.com/retrieve/pii/S088421751531515X. Acesso em: 02 ago. 2020.

DANSKI, Mitzy Tannia Reichembach et al. Incidência de complicações locais no cateterismo venoso periférico e fatores de risco associados. **Acta Paul Enferm**., [Internet], v. 28, n. 6, p. 517-523, dez. 2015. FapUNIFESP (SciELO). http://dx.doi.org/10.1590/1982-0194201500087. Disponível em: https://www.scielo.br/pdf/ape/v28n6/1982-0194-ape-28-06-0517.pdf. Acesso em: 01 nov. 2020.

DANTAS, Ana Márcia Nóbrega; GOMES, Gabriela Lisieux Lima; SILVA, Kenya de Lima; NÓBREGA, Maria Miriam Lima da. Diagnósticos de enfermagem para as etapas do crescimento e desenvolvimento de crianças utilizando a CIPE®. **Rev. Eletr. Enf**., [Internet], v. 18, p. 01-10, 30 jun. 2016. Universidade Federal de Goias. http://dx.doi.org/10.5216/ree.v18.35524. Disponível em: https://www.revistas.ufg.br/fen/article/view/35524/21457. Acesso em: 13 set. 2020.

DANTAS, Ana Márcia Nóbrega; SILVA, Kenya de Lima; NÓBREGA, Maria Miriam Lima da. Validation of nursing diagnoses, interventions and outcomes in a pediatric clinic. **Rev. Bras. Enferm**., [Internet], v. 71, n. 1, p. 80-88, fev. 2018. FapUNIFESP (SciELO). http://dx.doi.org/10.1590/0034-7167-2016-0647. Disponível em: https://www.scielo.br/scielo.php?script=sci_arttext&pid=S0034-71672018000100080&lng=en&tlng=en. Acesso em: 01 out. 2020.

DEL-BEN, Cristina Marta et al. Emergências psiquiátricas: manejo de agitação psicomotora e avaliação de risco suicida. **Medicina,** Ribeirão Preto, v.50, Supl 1, p. 98-112, 2017. http://dx.doi.org/10.11606/issn.2176-7262. Disponível em: https://edisciplinas.usp.br/pluginfile.php/4169746/mod_resource/content/2/Emerg%C3%AAncias%20psiqui%C3%A1tricas%20manejo%20de%20agita%C3%A7%C3%A3o%20psicomotora%20e%20avalia%C3%A7%C3%A3o%20de%20risco%20suicida%20.pdf. Acesso em: 20 out. 2020.

DINIZ, Lílian Martins Oliveira; FIGUEIREDO, Bruna de Campos Guimarães e. The newborn's immune system. **Rev Med Minas Gerais**, [Internet], v. 24, n. 2, p. 227-

233, 2014. GN1 Genesis Network. http://dx.doi.org/10.5935/2238-3182.20140056. Disponível em: http://rmmg.org/artigo/detalhes/1604. Acesso em: 01 out. 2020.

FREITAS, Edineia Machado de; NUNES, Zigmar Borges. O Enfermeiro na práxis de cateter central de inserção periférica em neonato. **remE - Rev. Min. Enferm.**, v. 13, n.2, p. 209-214, abr./jun., 2009. Disponível: www.reme.org.br/artigo/detalhes/182. Acesso em: 01 set. 2020.

DI SANTO, Marcelo Kalil et al. Cateteres venosos centrais de inserção periférica: alternativa ou primeira escolha em acesso vascular?. **J. vasc. bras.** [online], vol.16, n.2, p.104-112, 2017. http://dx.doi.org/10.1590/1677- 5449.011516. Disponível em: https://www.scielo.br/scielo.php?pid=S1677-54492017000200104&script=sci_abstract&tlng=pt. Acesso em: 01 set. 2020.

DIAS, Flávia de Souza Barbosa; MARBA, Sérgio Tadeu Martins. The evaluation of prolonged pain in the newborn: adaptation of the edin scale for the brazilian culture. **Texto Contexto - Enferm**., [Internet], v. 23, n. 4, p. 964-970, dez. 2014. FapUNIFESP (SciELO). http://dx.doi.org/10.1590/0104-07072014002100013. Disponível em: https://www.scielo.br/scielo.php?script=sci_arttext&pid=S0104-07072014000400964. Acesso em: 11 out. 2020.

DUESING, Lori A.; FAWLEY, Jason A.; WAGNER, Amy J. Central Venous Access in the Pediatric Population With Emphasis on Complications and Prevention Strategies. **Nutr Clin Pract**, [Internet], v. 31, n. 4, p. 490-501, 9 jul. 2016. Wiley. http://dx.doi.org/10.1177/0884533616640454. Disponível em: https://pubmed.ncbi.nlm.nih.gov/27032770/. Acesso em: 10 jun. 2020.

EMPRESA BRASILEIRA DE SERVIÇOS HOSPITALARES (EBSERH). Maternidade Escola Januário Cicco (MEJC). **Carta de Serviços ao cidadão**. 2019. Disponível em:http://www2.ebserh.gov.br/documents/16952/0/Carta+de+Servi%C3%A7os+ao+Cidad%C3%A3o+Atual+08+10+2019/f8e2ae36-4379-4198-8a11-9ca59efbcda7. Acesso em 15 ago. 2020.

FEHRING, R.J. Methods to validate nursing diagnoses. **Heart and Lung**, v. 16, n. 6, p. 625-9, nov., 1987.

FERREIRA, Carolina Pereira et al. A utilização de cateteres venosos centrais de inserção periférica na Unidade Intensiva Neonatal. **Rev. Eletr. Enferm**., [Internet], v. 22, p. 1-8, 30 jun. 2020. http://dx.doi.org/10.5216/ree.v22.56923. Disponível em: https://revistas.ufg.br/fen/article/view/56923. Acesso em: 27 jun. 2020.

FITZGERALD, Maria. What do we really know about newborn infant pain? **Exp Physiol**, [Internet], v. 100, n. 12, p. 1451-1457, 1 dez. 2015. Wiley. http://dx.doi.org/10.1113/ep085134. Disponível em: https://physoc.onlinelibrary.wiley.com/doi/full/10.1113/EP085134. Acesso em: 07 out. 2020.

GARCIA, Telma Ribeiro; NOBREGA, Maria Miriam Lima da. A terminologia CIPE® e a participação do Centro CIPE® brasileiro em seu desenvolvimento e disseminação. **Rev. Bras. Enferm.**, Brasília, v. 66, n. spe, p. 142-150, Sept. 2013.

https://doi.org/10.1590/S0034-71672013000700018. Disponível em: https://www.scielo.br/scielo.php?pid=S0034-71672013000700018&script=sci_abstract&tlng=pt. Acesso em 20 jun. 2020.

GARCIA, TR. **Classificação Internacional para a Prática de Enfermagem (CIPE®): versão 2019**. Porto Alegre: Artmed, 2019.

GHYSELEN, Laurent et al. Polyethylene bag wrapping to prevent hypothermia during percutaneous central venous catheter insertion in the preterm newborn under 32 weeks of gestation. **J Matern Fetal Neonatal Med**., [Internet], v. 27, n. 18, p. 1922-1925, 12 fev. 2014. Informa UK Limited. http://dx.doi.org/10.3109/14767058.2014.885498. Disponível em: https://pubmed.ncbi.nlm.nih.gov/24438464/. Acesso em: 02 ago. 2020.

GOMES, Bárbara Machado; MENDES, João Luís Lopes; PEDRO, Adriano Dias. Cuidados de Enfermagem associados ao cateterismo venoso periférico. **Revista Ibero-Americana de Saúde e Envelhecimento**, [Internet], v. 6, n. 1, p. 2135-2149, abr. 2020. ISSN 2183-6663. Disponível em: http://www.revistas.uevora.pt/index.php/saude_envelhecimento/article/view/406/674. Acesso em: 17 nov. 2020.

GOMES, Thainá Castro et al. Os cuidados de enfermagem na prevenção de infecção relacionados ao PICC em unidade neonatal. **Saúde Coletiva** (Barueri), [S. l.], v. 9, n. 48, p. 1404 - 1415, 2019. Disponível em: http://revistas.mpmcomunicacao.com.br/index.php/saudecoletiva/article/view/103. Acesso em: 17 nov. 2020.

GORSKI, Lisa et al. Infusion Therapy Standards of Practice. **Journal of Infusion Nursing**. V. 39, n. 1S, 2016. Disponível em: https://source.yiboshi.com/20170417/1492425631944540325.pdf. Acesso em: 02 nov. 2020.

HALABY, Lori P.; STEINKRAUSS, Linda. Hypoglycemia: symptom or diagnosis?. **J Pediatr Nurs**., [Internet], v. 27, n. 1, p. 97-99, fev. 2012. Elsevier BV. http://dx.doi.org/10.1016/j.pedn.2011.10.005. Disponível em: https://pubmed.ncbi.nlm.nih.gov/22064214/. Acesso em: 12 out. 2020.

International Council of Nurses (ICN). **Guidelines for ICNP® Catalogue Development** [Internet]. Genebra, ICN, 2008.

International Council of Nurses (ICN). **International Classification for Nursing Practice – ICNP® Version 1**. Geneva; 2005.

JOHANN, Derdried Athanasio et al. Cuidados com cateter central de inserção periférica no neonato: revisão integrativa da literatura. **Rev. esc. enferm. USP**, São Paulo, v. 46, n. 6, p.1503-1511, dez. 2012. https://doi.org/10.1590/S0080-62342012000600030. Disponível em: http://www.scielo.br/scielo.php?script=sci_arttext&pid=S0080-62342012000600030&lng=en&nrm=iso. Acesso em: 01 set. 2020.

LUCENA, Amália de Fátima et al. Diagnóstico de enfermagem risco de sangramento como indicador de qualidade da assistência para segurança do paciente. **Rev. Gaúcha Enferm**., Porto Alegre, v. 40, n. spe, e20180322, 2019. http://dx.doi.org/10.1590/1983-1447.2019.20180322. Disponível em: http://www.scielo.br/scielo.php?script=sci_arttext&pid=S1983-14472019000200805&lng=en&nrm=is. Acesso em: 01 out. 2020.

KASAP, Tuba et al. Neonatal Thrombocytopenia and the Role of the Platelet Mass Index in Platelet Transfusion in the Neonatal Intensive Care Unit. **Balkan Med J**., [Internet], v. 37, n. 3, p. 150-156, 12 abr. 2020. Galenos Yayinevi. http://dx.doi.org/10.4274/balkanmedj.galenos.2020.2019.7.47. Disponível em: https://www.ncbi.nlm.nih.gov/pmc/articles/PMC7161623/. Acesso em: 01 out. 2020.

KHANNA, Vinay et al. Evaluation of Central Venous Catheter Associated Blood Stream Infections: a microbiological observational study. **Journal Of Pathogens**, [Internet], v. 2013, p. 01-6, 2013. Hindawi Limited. http://dx.doi.org/10.1155/2013/936864. Disponível em: https://www.ncbi.nlm.nih.gov/pmc/articles/PMC3723241/. Acesso em: 08 out. 2020.

KNOBEL, Robin; HOLDITCH-DAVIS, Diane. Thermoregulation and Heat Loss Prevention After Birth and During Neonatal Intensive-Care Unit Stabilization of Extremely Low-Birthweight Infants. **Adv Neonatal Care**., [Internet], v. 10, p. 07- 14, out. 2010. Ovid Technologies (Wolters Kluwer Health). http://dx.doi.org/10.1097/anc.0b013e3181ef7de2. Disponível em: https://pubmed.ncbi.nlm.nih.gov/20838082/. Acesso em: 10 out. 2020.

KONSTANTINIDI, Aikaterini et al. Umbilical Venous Catheters and Peripherally Inserted Central Catheters: are they equally safe in vlbw infants? a non- randomized single center study. **Medicina**, [Internet], v. 55, n. 8, p. 442-453, 6 ago. 2019. MDPI AG. http://dx.doi.org/10.3390/medicina55080442. Disponível em: https://www.ncbi.nlm.nih.gov/pmc/articles/PMC6723053/. Acesso em: 14 jun. 2020.

LEADEBAL, Oriana Deyze Correia Paiva; FONTES, Wilma Dias de; SILVA, César Cavalcanti da. Ensino do processo de enfermagem: planejamento e inserção em matrizes curriculares. **Rev. esc. enferm. USP**, [Internet], v. 44, n. 1, p. 190-198, mar. 2010. FapUNIFESP (SciELO). http://dx.doi.org/10.1590/s0080-62342010000100027. Disponível em: https://www.scielo.br/scielo.php?script=sci_arttext&pid=S0080-62342010000100027. Acesso em: 01 ago. 2020.

LIENEAMANN, Márcia; TAKASHASHI, Luciane da Silva; DOS SANTOS, Reginaldo Passoni. Acesso vascular em neonatologia: cateter central de inserção periférica e cateter venoso periférico. **Rev. Fac. Ciênc. Méd**. Sorocaba, v. 16, n. 1, p. 1 - 3, 2014. Disponível em: http://revistas.pucsp.br/index.php/RFCMS/article/view/17473. Acesso em: 01 set. 2020.

LIU, Jing; CHEN, Xin-Xin; WANG, Xin-Ling. Ethical issues in neonatal intensive care units. **J Matern Fetal Neonatal Med**, [Internet], p. 1-5, 18 set. 2015. Informa UK Limited. http://dx.doi.org/10.3109/14767058.2015.1085016. Disponível em: https://pubmed.ncbi.nlm.nih.gov/26382713/. Acesso em: 10 jun. 2020.

LOPES, Marcos Venícios de Oliveira; SILVA, Viviane Martins da; ARAUJO, Thelma Leite de. Validação de diagnósticos de enfermagem: desafios e alternativas. **Rev. Bras. Enferm.**, [Internet], v. 66, n. 5, p. 649-655, out. 2013. FapUNIFESP (SciELO). http://dx.doi.org/10.1590/s0034-71672013000500002. Disponível em: https://www.scielo.br/pdf/reben/v66n5/02.pdf. Acesso em: 20 ago. 2020.

MATA, Luciana Regina Ferreira da et al. Elaboração de diagnósticos e intervenções à luz de diferentes sistemas de classificações de enfermagem. **Rev. esc. enferm. USP**, São Paulo, v. 46, n. 6, p. 1512-1518, dez. 2012. http://dx.doi.org/10.1590/S0080-62342012000600031. Disponível em: http://www.scielo.br/scielo.php?script=sci_arttext&pid=S0080-62342012000600031&lng=en&nrm=iso. Acesso em 15 ago 2020.

MARTINS, Cláudia; CURADO, Maria. Observation Neonatal Skin Risk Assessment Scale: statistical validation with newborns. **Rev. Enf. Ref.**, [Internet], n. 13, p. 43-52, 14 jun. 2017. Health Sciences Research Unit: Nursing. http://dx.doi.org/10.12707/riv16082. Disponível em: http://www.scielo.mec.pt/scielo.php?script=sci_arttext&pid=S0874-02832017000200005&lng=pt&nrm=iso. Acesso em: 11 out. 2020.

MARTINS, Erika Miller et al. Os Agravos no Desenvolvimento Neurocomportamental do Recém-Nascido Prematuro: ênfase no manuseio. **RETEP - Rev. Tendên. da Enferm. Profis**., [Internet], v. 7, n.1, p. 1481-85, 2015. Disponível em: http://www.coren-ce.org.br/wp- content/uploads/2015/12/retep-7-1-web.pdf. Acesso em: 02 out. 2020.

MATTEI, Francine Dutra et al. Uma visão da produção científica internacional sobre a classificação internacional para a prática de enfermagem. **Rev Gaúcha Enferm**., [Internet], v. 32, n. 4, p. 823-831, dez. 2011. FapUNIFESP (SciELO). http://dx.doi.org/10.1590/s1983-14472011000400025. Disponível em: https://www.scielo.br/scielo.php?script=sci_abstract&pid=S1983-14472011000400025&lng=en&nrm=iso&tlng=pt. Acesso em 20 jun. 2020.

MAZONI, Simone Roque et al. Classificação Internacional para a Prática de Enfermagem e a contribuição brasileira. **Rev. Bras. Enferm**., Brasília, v. 63, n. 2, p. 285-289, abr. 2010. https://doi.org/10.1590/S0034-71672010000200018. Disponível em: http://www.scielo.br/scielo.php?script=sci_arttext&pid=S0034-71672010000200018&lng=en&nrm=iso. Acesso em 10 ago 2020.

MONTES, SF et al. Ocorrência de Complicações relacionadas ao uso do Cateter Venoso Central de Inserção Periférica (PICC) em recém nascidos. **Enferm. glob**., Murcia, n.24, outubro de 2011. Disponível em: http://scielo.isciii.es/pdf/eg/v10n24/pt_clinica1.pdf. Acesso em: 01 set. 2020.

MORAES, Etiene Leticia Leone de; FREIRE, Márcia Helena de Souza. Painful and stressful procedures and analgesia in newborns from the viewpoint of professionals. **Rev. Bras. Enferm**., [Internet], v. 72, n. 3, p. 170-177, dez. 2019. FapUNIFESP (SciELO). http://dx.doi.org/10.1590/0034-7167-2018-0326. Disponível em:

https://www.scielo.br/scielo.php?pid=S0034-71672019000900170&script=sci_arttext&tlng=pt#B5. Acesso em: 02 ago. 2020.

MOTTA, Patrícia das Neves et al. Cateter central de inserção periférica: o papel da Enfermagem na sua utilização em Neonatologia. **HU Revista**, v. 37, n. 2, 23 mar. 2012. Disponível em: https://periodicos.ufjf.br/index.php/hurevista/article/view/1402. Acesso em: 14 ago. 2020.

MOTTA, Giordana de Cássia Pinheiro da; CUNHA, Maria Luzia Chollopetz da. Prevenção e manejo não farmacológicos da dor no recém-nascido. **Rev Bras Enferm**., [Internet], v. 68, n. 1, p. 131-135, fev. 2015. FapUNIFESP (SciELO). Disponível em: https://www.scielo.br/pdf/reben/v68n1/0034-7167-reben-68-01-0131.pdf. Acesso em: 10 ago. 2020.

MCMILLAN, S.S.; KING, M.; TULLY, M.P. How to use the nominal group and Delphi techniques. **Int J Clin Pharm** [Internet]. 2016 [cited dec 30, 2017]; 38:655–62. Available from: https://www.ncbi.nlm.nih.gov/pmc/articles/PMC4909789/

NAKANDAKARI, Regina Ayumi et al. Práticas de enfermagem relacionadas à cateterização intravenosa periférica em recém-nascidos e crianças. **Rev. Soc. Bras. Enferm. Ped**., [Internet], v. 18, n. 1, p. 29-36, jun. 2018. Sociedade Brasileira de Enfermeiros Pediatras. http://dx.doi.org/10.31508/1676-3793201800005. Disponível em: https://sobep.org.br/revista/images/stories/pdf-revista/vol18-n1/vol_18_n_1-artigo_original_4.pdf. Acesso em: 01 nov. 2020.

NEUMAN, B. M. **The Neuman systems model**. 5ª Edition. Ohio: Appleton & Lange, 1995.

NEUMAN, Betty; FAWCETT, Jacqueline. **The Neuman Systems Model**. 5ª Ed. 2011.

NOBREGA, Maria Miriam Lima da; GARCIA, Telma Ribeiro. Perspectivas de incorporação da Classificação Internacional para a Prática de Enfermagem (CIPE®) no Brasil. **Rev. Bras. Enferm**., Brasília, v. 58, n. 2, p. 227-230, 2005. Disponível em: http://www.scielo.br/scielo.php?script=sci_arttext&pid=S0034-71672005000200020&lng=en&nrm=iso. Acesso em 10 ago. 2020.

OLIVEIRA, Cheila Gonçalves de; RODAS, Andrea Cecilia Dorion. Tecnovigilância no Brasil: panorama das notificações de eventos adversos e queixas técnicas de cateteres vasculares. **Ciênc. saúde colet**., [Internet], v. 22, n. 10, p. 3247-3257, out. 2017. FapUNIFESP (SciELO). Disponível em: https://www.scielo.br/scielo.php?pid=S1413-81232017021003247&script=sci_abstract&tlng=pt. Acesso em: 28 jun. 2020.

OLIVEIRA, Cristine Ruviaro de et al. Cateter central de inserção periférica em pediatria e neonatologia: possibilidades de sistematização em hospital universitário. **Esc. Anna Nery**, Rio de Janeiro, v. 18, n. 3, p. 379-385, Sept. 2014. https://doi.org/10.5935/1414-8145.20140054. Disponível em:

http://www.scielo.br/scielo.php?script=sci_arttext&pid=S1414-81452014000300379&lng=en&nrm=iso. Acesso em: 29 jun. 2020.

OLIVEIRA, Mariana Chaves et al. Medidas não farmacológicas para o alívio da dor no recém-nascido prematuro na UTI neonatal. **Saúde coletiva**, [Internet], v.9, n.49, p. 1483-87, 2019. Disponível em: http://revistas.mpmcomunicacao.com.br/index.php/saudecoletiva/article/view/112/96. Acesso em 10 ago. 2020.

ORSI, Kelly Cristina Sbampato Calado et al. Effect of reducing sensory and environmental stimuli during hospitalized premature infant sleep. **Rev. Esc. Enferm. Usp**., [Internet], v. 49, n. 4, p. 0550-0555, ago. 2015. FapUNIFESP (SciELO). http://dx.doi.org/10.1590/s0080-623420150000400003. Disponível em: https://www.scielo.br/scielo.php?script=sci_arttext&pid=S0080-62342015000400550. Acesso em: 11 out. 2020.

PACHECO, Sandra Teixeira de Araújo et al. O cuidado pelo enfermeiro ao recém-nascido prematuro frente à punção venosa. **Rev. enferm. UERJ**, Rio de Janeiro, v. 20, n. 3, p. 306-11, 2012. Disponível em: https://www.e-publicacoes.uerj.br/index.php/enfermagemuerj/article/view/3150. Acesso em: 10 jun. 2020.

PADILLA-SÁNCHEZ, C. et al. Factores de riesgo asociados a eventos adversos en recién nacidos portadores de catéteres centrales de inserción periférica. **Enferm Intensiva**, [Internet], v. 30, n. 4, p. 170-180, out. 2019. Elsevier BV. http://dx.doi.org/10.1016/j.enfi.2018.10.006. Disponível em: https://pesquisa.bvsalud.org/portal/resource/pt/ibc-184481. Acesso em: 10 jun. 2020.

PEREIRA, Fabiola Lima et al. A manipulacao de prematuros em uma Unidade de Terapia Intensiva Neonatal. **Rev. Esc. Enferm. Usp**, [Internet], v. 47, n. 6, p. 1272-1278, 1 dez. 2013. FapUNIFESP (SciELO). http://dx.doi.org/10.1590/s0080-623420130000600003. Disponível em: https://www.scielo.br/scielo.php?script=sci_arttext&pid=S0080-62342013000601272&lng=pt&tlng=pt. Acesso em: 07 out. 2020.

PEREIRA, Maurício Gomes. **Epidemiologia: teoria e prática**. Rio de Janeiro: Guanabara Koogan; 2013.

PERES, Camille Farias et al. As complicações da inserção e manipulação do cateter central de inserção periférica. **Saúde Coletiva** (Barueri), [Internet], v. 9, n. 50, p. 1779 - 1783, 2020. Disponível em: http://revistas.mpmcomunicacao.com.br/index.php/saudecoletiva/article/view/157. Acesso em: 18 nov. 2020

PESUT, Danil; HERMAN, Jo Anne. **Clinical reasoning: the art and science of critical and creative thinking**. Albany (NY): Delmar; 1999.

PET, Gillian C et al. Risk factors for peripherally inserted central catheter complications in neonates. **J Perinatol**., [Internet], v. 40, n. 4, p. 581-588, 7 jan. 2020. Springer Science and Business Media LLC. http://dx.doi.org/10.1038/s41372-

019-0575-7. Disponível em: https://pubmed.ncbi.nlm.nih.gov/31911643/. Acesso em: 12 ago. 2020.

PIMENTA, Adriana de Lima; SOUZA, Maria de Lourdes de. The professional identity of nursing in the papers published by reben. **Texto contexto - enferm.**, Florianópolis, v. 26, n. 1, e4370015, 2017. Epub Feb 06, 2017. https://doi.org/10.1590/0104-07072016004370015. Disponível em: https://www.scielo.br/scielo.php?script=sci_arttext&pid=S0104-07072017000100304. Acesso em: 14 jun. 2020.

POLIT DF, BECK CT. **Fundamentos de pesquisa em enfermagem: avaliação de evidências para a prática da enfermagem**. 7a ed. Porto Alegre: Artmed; 2011.

PRADO, Nanete Caroline da Costa et al. Variables asociadas a eventos adversos en neonatos con catéter venoso central de inserción periférica. **Enferm. glob.**, Murcia, v. 19, n. 59, p. 36-67, 2020. http://dx.doi.org/10.6018/eglobal.387451. Disponível em: http://scielo.isciii.es/scielo.php?script=sci_arttext&pid=S1695-61412020000300036. Acesso em: 29 jun. 2020.

PRESTES, Ana Claudia Yoshikumi et al. Procedimentos dolorosos e analgesia em UTI Neonatal: o que mudou na opinião e na prática profissional em dez anos?. **J. Pediatr**. [Internet], Porto Alegre, v. 92, n. 1, p. 88-95, Fev. 2016. https://doi.org/10.1016/j.jped.2015.04.009. Disponível em: http://www.scielo.br/scielo.php?script=sci_arttext&pid=S0021-75572016000100088&lng=en&nrm=iso. Acesso em: 27 ago. 2020.

REBELATO, Cibele Thomé da Cruz; STUMM, Eniva Miladi Fernandes. Analysis of pain and free cortisol of newborns in intensive therapy with therapeutic procedures. **BrJP,** [Internet], v. 2, n. 2, p. 159-165, 2019. GN1 Genesis Network. http://dx.doi.org/10.5935/2595-0118.20190029. Disponível em: https://www.scielo.br/pdf/brjp/v2n2/pt_2595-0118-brjp-02-02-0159.pdf. Acesso em: 12 out. 2020.

ROSA, Michelly Kim de Oliveira; GAÍVA, Maria Aparecida Munhoz. Qualidade da atenção hospitalar ao recém-nascido. **Rev. Rene**. Fortaleza, v. 10, n. 1, p. 159-165, jan./mar.2009. Disponível em: http://periodicos.ufc.br/rene/article/view/4755/3524. Acesso em 01 out. 2020.

RUSSO, Natalia Conteçote et al. O enfermeiro na prevenção de infecção no cateter central de inserção periférica no neonato. **Vigil. Sanit. Debate**, [Internet], v. 8, n. 2, p. 134-143, 29 maio 2020. Vigilancia Sanitaria em Debate: Sociedade, Ciencia y Tecnologia. http://dx.doi.org/10.22239/2317-269x.01414. Disponível em: https://visaemdebate.incqs.fiocruz.br/index.php/visaemdebate/article/view/1414. Acesso em: 10 nov. 2020.

SILVA, Carolina Barbosa et al. Ocorrência de eventos adversos em Unidade de Terapia Intensiva Neopediátrica: qualidade assistencial de enfermagem. **R Epidemiol Control Infec**, Santa Cruz do Sul, v. 7, n. 4, p. 241-245, 1 out. 2017. APESC - Associacao Pro-Ensino em Santa Cruz do Sul.

http://dx.doi.org/10.17058/reci.v7i4.7564. Disponível em: https://www.e-publicacoes.uerj.br/ojs/index.php/enfermagemuerj/article/view/6076. Acesso em: 27 jun. 2020.

SILVA, Eliane Cristina da et al. Gerenciamento do tempo das ações de enfermagem no cuidado à criança e ao adolescente hospitalizados. **Rev enferm UFPE online**., Recife, v. 8, n. 11, p. :3920-8, nov., 2014. Disponível em: https://periodicos.ufpe.br/revistas/revistaenfermagem/article/view/13616/16452. Acesso em: 02 ago. 2020.

SOARES, Mirelle Inácio et al. Systematization of nursing care: challenges and features to nurses in the care management. **Esc. Anna Nery**, [Internet], v. 19, n. 1, p. 47-53, 2015. FapUNIFESP (SciELO). http://dx.doi.org/10.5935/1414-8145.20150007. Disponível em: https://www.scielo.br/scielo.php?pid=S1414-81452015000100047&script=sci_arttext&tlng=en. Acesso em: 13 jun. 2020.

SOARES, Tamara et al. Prevalence of hypothermia in the first hour of life of premature infants weighing ≤ 1500g. **Rev Gaúcha Enferm**., [Internet], v. 41, (esp):e20190094, p. 01-09, 2020. FapUNIFESP (SciELO). http://dx.doi.org/10.1590/1983-1447.2020.20190094. Disponível em: https://www.seer.ufrgs.br/RevistaGauchadeEnfermagem/article/viewFile/98371/54901. Acesso em: 08 out. 2020.

SOUSA, Fernanda Coura Pena de et al. A participação da família na segurança do paciente em unidades neonatais na perspectiva do enfermeiro. **Texto Contexto Enferm**., [Internet], v.26, n.6, p.1-8, Florianópolis 2017. https://doi.org/10.1590/0104-07072017001180016. Disponível em: https://www.scielo.br/scielo.php?script=sci_arttext&pid=S0104-07072017000300314. Acesso em: 06 out. 2020.

SOUZA, Marília Fernandes Gonzaga de; DOS SANTOS, Ana Dulce Batista; MONTEIRO, Akemi Iwata. O processo de enfermagem na concepção de profissionais de Enfermagem de um hospital de ensino. **Rev. Bras. Enferm**.,[Internet], v. 66, n. 2, p. 167-73, 2013. Disponível em: https://www.scielo.br/pdf/reben/v66n2/03.pdf. Acesso em: 01 ago. 2020.

SOUZA, Raissa Silva et al. Prevenção de infecções associadas a cateteres periféricos: construção e validação de cenário clínico. **Rev. Bras. Enferm**. [Internet]. 2020, vol.73, n.5 [cited 2020-11-17], e20190390. Disponível em: http://www.scielo.br/scielo.php?script=sci_arttext&pid=S0034-71672020000500177&lng=en&nrm=iso. Epub July 06, 2020. ISSN 1984-0446. Acesso em: 11 nov. 2020.

SPIRONELLO, Ricardo Alexandre; CUMAN, Roberto Kenji Nakamura. Caracterização de eventos adversos em uma unidade de terapia intensiva neonatal. **Revista Recien**., São Paulo, v.28, n.9, p. 131-136, 2019. Disponível em: https://recien.com.br/index.php/Recien/article/view/324. Acesso em: 27 jun. 2020.

SPOSITO, Natália Pinheiro Braga et al. Avaliação e manejo da dor em recém-nascidos internados em Unidade de Terapia Intensiva Neonatal: estudo transversal.

Rev. Latino-Am. Enfermagem, [Internet], v. 25, p. 01-09, 12 set. 2017. FapUNIFESP (SciELO). http://dx.doi.org/10.1590/1518-8345.1665.2931. Disponível em: https://www.scielo.br/pdf/rlae/v25/0104-1169-rlae-25-e2931.pdf. Acesso em: 10 ago. 2020. https://doi.org/10.25248/reas.e2268.2020. Disponível em: https://www.acervomais.com.br/index.php/saude/article/view/2268/1282. Acesso em 26 ago. 2020.

SWERTS, Cátia Aline Silva et al. A utilização do cateter central de inserção periférica em uma unidade de terapia intensiva neonatal. **REAS/EJCH**, n. 40, p. e2268, 14 fev. 2020.

TOMEY, Ann Marriner; ALLIGOOD, Martha Raile. **Teóricas de Enfermagem e a Sua Obra (Modelos e Teorias de Enfermagem).** 5ªed. Loures: Lusociência, 2004.

VALADÃO, Vanessa Pereira Caldas et al. Avaliação do uso da manta térmica em recém-nascidos submetidos à instalação do Cateter Venoso Central de Inserção Periférica. **Revista Nursing**., [Internet], v.22, n.259, p.3419-3425, 2019. Disponível em: http://www.revistanursing.com.br/revistas/259/pg61.pdf. Acesso em: 02 ago. 2020.

VIEIRA, Giovana Mereguete et al. Protocolo de enfermagem para assistência à mulher em processo de lactação. **J. res.: fundam. care. online**, [Internet], v. 9, n. 4, p. 1040-1047, oct. 2017. Disponível em: http://www.seer.unirio.br/index.php/cuidadofundamental/article/view/5768. Acesso em 18 jun. 2020.

VIEIRA, Sônia. **Introdução à Bioestatística**. 4.ed. Rio de Janeiro: Elsevier, 2010.

WESTERGAARD, B.; CLASSEN, V.; WALTHER-LARSEN, S. Peripherally inserted central catheters in infants and children - indications, techniques, complications and clinical recommendations. **Acta Anaesthesiol Scand**, [Internet], v. 57, n. 3, p. 278-287, 17 dez. 2012. Wiley. Disponível em: https://pubmed.ncbi.nlm.nih.gov/23252685/. Acesso em: 10 jun. 2020.

World Health Organization (CH). **Maternal and newborn Health. Division of Reproductive Health. Termal protection of the newborn: a practical guide**. Geneva: WHO; 1997. Disponível em: https://apps.who.int/iris/bitstream/handle/10665/63986/WHO_RHT_MSM_97.2. pdf. Acesso em: 10 out. 2020.

YU, Xiaohe et al. Risk Factors Related to Peripherally Inserted Central Venous Catheter Nonselective Removal in Neonates. **Biomed Res Int**, [Internet], v. 2018, p. 1-6, 30 maio 2018. Hindawi Limited. Disponível em: https://www.ncbi.nlm.nih.gov/pmc/articles/PMC5998161/. Acesso em: 10 jun. 2020.

YURA, Helena; WALSH Mary B. **The nursing process: assessment, planning, implementation and evaluation.** New York (USA): Appleton-Century-Crofts; 1967.

ANEXOS

UFRN - HOSPITAL UNIVERSITÁRIO ONOFRE LOPES DA UNIVERSIDADE FEDERAL DO RIO GRANDE DO NORTE - HUOL/UFRN

PARECER CONSUBSTANCIADO DO CEP

DADOS DA EMENDA

Título da Pesquisa: Sistematização da Assistência de Enfermagem em recém-nascidos com Cateter Central de Inserção Periférica em Unidade de Terapia Intensiva.

Pesquisador: NANETE CAROLINE DA COSTA PRADO

Área Temática:

Versão: 2

CAAE: 48990515.0.0000.5292

Instituição Proponente: Maternidade Escola Januário Cicco

Patrocinador Principal: Financiamento Próprio

DADOS DO PARECER

Número do Parecer: 4.199.441

Apresentação do Projeto:

Emenda a projeto Sistematização da Assistência de Enfermagem em recém-nascidos com Cateter Central de Inserção Periférica em Unidade de Terapia Intensiva.

Objetivo da Pesquisa:

Sistematizar o atendimento de bebes na UTI da maternidade.

Avaliação dos Riscos e Benefícios:

Descritos.

Comentários e Considerações sobre a Pesquisa:

Trata-se de uma emenda a projeto da Enfermagem nos bebês da MEJC.

Considerações sobre os Termos de apresentação obrigatória:

Todos presentes

Conclusões ou Pendências e Lista de Inadequações:

Aprovada modificações

Considerações Finais a critério do CEP:

Emenda aprovada pois não houve mudanças em objetivos ou métodos, apenas pequenas modificações incluindo cronograma.

Endereço: Avenida Nilo Peçanha, 620 - Prédio Administrativo - 1º Andar - Espaço João Machado
Bairro: Petrópolis **CEP:** 59.012-300
UF: RN **Município:** NATAL
Telefone: (84)3342-5003 **Fax:** (84)3202-3941 **E-mail:** cep_huol@yahoo.com.br

Este parecer foi elaborado baseado nos documentos abaixo relacionados:

Tipo Documento	Arquivo	Postagem	Autor	Situação
Informações Básicas do Projeto	PB_INFORMAÇÕES_BÁSICAS_1583427_E1.pdf	07/07/2020 15:39:57		Aceito
Outros	MODIFICADOProjetodePesquisa.pdf	07/07/2020 15:37:15	NANETE CAROLINE DA COSTA PRADO	Aceito
Outros	EMENDA_PROJETO_MODIFICADO.pdf	24/06/2020 23:21:39	NANETE CAROLINE DA COSTA PRADO	Aceito
Outros	CARTA_DE_EMENDA_AO_CEP.pdf	24/06/2020 23:19:34	NANETE CAROLINE DA COSTA PRADO	Aceito
Outros	Identificacao_do_Pesquisador.pdf	03/09/2015 10:28:10	NANETE CAROLINE DA COSTA PRADO	Aceito
TCLE / Termos de Assentimento / Justificativa de Ausência	TCLE.docx	03/09/2015 10:24:02	NANETE CAROLINE DA COSTA PRADO	Aceito
Outros	Carta de Anuência.pdf	13/08/2015 20:53:59		Aceito
Folha de Rosto	Folha de Rosto.pdf	13/08/2015 20:53:10		Aceito
Projeto Detalhado / Brochura Investigador	Projeto de Pesquisa.pdf	12/08/2015 20:43:50		Aceito

Situação do Parecer:
Aprovado

Necessita Apreciação da CONEP:
Não

NATAL, 10 de Agosto de 2020

Assinado por:
jose diniz junior
(Coordenador(a))

Endereço: Avenida Nilo Peçanha, 620 - Prédio Administrativo - 1º Andar - Espaço João Machado
Bairro: Petrópolis **CEP:** 59.012-300
UF: RN **Município:** NATAL
Telefone: (84)3342-5003 **Fax:** (84)3202-3941 **E-mail:** cep_huol@yahoo.com.br

APÊNDICES

INSTRUMENTOS DE COLETA DE DADOS

**1.Identificação**			
1.1 RN de (Iniciais):	1.2 DN:	1.3 Sexo: 1.( )feminino 2.( )masculino	
1.4 Tempo de Internação:	1.6 Procedência: 1.( )Capital/RN 2.( )Interior/RN 3.( )Outro:		
1.5 Peso: 1.( )Normal: >2501g e <4000g 2.( )Baixo Peso: >1501g e <2500g<br>3.( )Muito Baixo Peso: >1001g e <1500g 4.( )Extremo Baixo Peso: <1000g<br>5.( )Macrossômico: >4001g.			
1.6 Indicação para uso do PICC:			
**2.Dados do Cateter**			
2.1 Marca:	2.2 Lote:	2.3 Calibre:	2.4 Comp.:
**3.Inserção**			
3.1 Membro:	3.2 Perímetro do membro:		
3.3 Comprimento que foi introduzido:	3.4 Nº de tentativas:		
3.5 Volume de SF 0,9% introduzido:	3.6 RX após inserção:1.( )não 2.( )sim		
3.7 Dificuldade de progressão do cateter: 1.( )não 2.( )sim			
3.8 Localização da ponta do cateter:			
3.9 Intercorrências:1.( )não 2.( )sim, se sim, quais:			
3.10 Data e Hora:			
3.11 Profissional responsável: 1.( )enfermeiro 2.( )médico			
**4.Troca do 1º Curativo**			
4.1 Tipo: 1.( )Gaze com micropore 2.( ) Filme transparente			
4.2 Avaliação do sítio de inserção: 1.( )ausência de sinais flogísticos 2.( )secreção purulenta<br>3.( )hiperemia 4.( )edema 5.( )hematoma 6.( )sangramento 7.( )secreção serosa			
4.3 Solução utilizada para antissepsia:			
4.4 Data e Hora:			
4.5 Profissional responsável: 1.( )enfermeiro 2.( )médico			
**5.Retirada**			
5.1 Motivo:			
5.2 Comprimento retirado:			
5.3 Enviado para cultura:1.( )não 2.( )sim			
5.4 Isolado microorganismo:1.( )não 2.( )sim<br>Se sim, qual:			
5.6 Data e Hora:			
5.7 Profissional responsável: 1.( )enfermeiro 2.( )médico			

**1.Identificação Materna**			
1.1 Iniciais da Mãe:	1.2 Idade:	1.3 Data da Coleta:	1.4 Ocupação:
1.5 Motivo da Internação:<br>1.7 Prontuário:			1.6 União conjugal:<br>1.( )sem cônjuge 2.( )com cônjuge
1.8 Município de Residência: 1.( )Capital/RN 2.( )Interior/RN 3.( )Outro:			
1.9 Escolaridade:<br>1.( )Analfabeto 2.( )Fundamental Incompleto<br>3.( )Fundamental Completo<br>4.( )Médio Incompleto 5.( )Médio Completo<br>6.( )Superior Incompleto 7.( )Superior Completo			1.10 Renda familiar (salários mínimos):<br>1.( )Sem rendimento 2.( )Até 1<br>3.( )Mais de 1 a 2 4.( ) Mais de 2 a 3 5.( )Mais de 3 a 5 6.( )Mais de 5 a 10<br>7.( )Mais de 10

**2.Anamnese**		
2.1 Antecedentes Familiares:<br>2.1.1 Diabetes ( )<br>2.1.2 Hipertensão ( )<br>2.1.3 Cardiopatia ( )<br>2.1.4 Infecções ( )<br>2.1.5 Gemelaridade ( )<br>2.1.6 Internações ( )<br>2.1.7 Outros:	2.2 Antecedentes Pessoais:<br>2.2.1 Diabetes ( )<br>2.2.2 Hipertensão ( )<br>2.2.3 Cardiopatia ( )<br>2.2.4 Infecções ( )<br>2.2.5 Tabagismo ( )<br>2.2.6 Etilismo ( )<br>Outros:	2.3 Antecedentes Obstétricos:<br>2.3.1 G: Pré-termo: Termo:<br>2.3.2 P: Vaginal:Cesárea: Fórceps:<br>2.3. A: Espontâneo: Provocado:<br>2.3.4 Natimortos:<br>2.3.5 Nativivos:<br>2.3.6 Data do último parto: / /
2.4 Grupo Sanguíneo/Fator Rh da Mãe:		2.5 Grupo Sanguíneo/Fator Rh do RN:

**3.Gestação Atual:**			
3.1 DUM:	3.2 IG:	3.3 IG USG:	3.4 DPP:
3.5 VDRL: / /<br>Resultado:	3.6 HIV: / /<br>Resultado:	3.7 Pré-Natal:<br>1.( )Não 2.( )Sim	3.8 Consultas:

**4.Identificação do RN:**			
4.1 DN:/ / 4.2 Hora:	4.3Tipo de Parto:	4.4 APGAR	
4.5Rotura Bolsa:	4.6Sexo:	4.7Peso:	4.8Est.:
4.9 Indicação do Parto:			
4.10 Motivo de Internação:			
4.11 Problema de saúde:			
4.12 Tempo de Internação:			

**5. Sinais Vitais:**			
5.1TAXºC:	5.2 FR(mrpm):	5.3 FC(bpm):	5.4 PA(mmHg):
5.5 Temp. incubadoraºC:	5.6 Sat.O2%:	5.7 Pulso:	5.8 Peso atual:

**6. Aspecto Geral**
1.( )Ativo 2.( )Reativo 3.( )Hipoativo 4.( )Hiporreativo 5.( )Sedado 6.( )Comatoso 7.( )Hipotônico<br>8.( )Edema

**7. Integridade Física/Cutâneo Mucosa**
1.( )Hidratada 2.( )Ressecada 3.( )Normocorada 4.( )Hipocorada 5.( )Pletórica 6.( )Cianose<br>7.( )Mosqueada 8.( )Normotermia 9.( )Hipertermia 10.()Hipotermia 11.( )Extremidades frias<br>12.( )Icterícia 13.( )Anictérica14.( )Fototerapia 15.( ) Infiltrações 16.( )Hematomas 17.( )Dermatite<br>18.( )Úlcera por pressão, Local:
7.1 Turgor: 1.( )Bom 2.( )Regular 3.( )Ruim

8. Cabeça e Pescoço
**8.1 Cabeça:** 1.( )Anormal 2.( )Normal 2.( )Bossa 3.( )Fraturas 4.( )Céfalo hematoma
**8.2 Sujidade:**1.( )não 2.( )sim, Local:
**8.3 Outras anormalidades:**
**8.4 Olhos e Pálpebras:** 1.( )Abertura espontânea 2.( )Fechado 3.( )Fixo 4.( )Secreções 5.( )Edema 6.( )Hemorragia
**8.5 Pupilas:**1.( )Isocóricas 2.( )Anisocóricas<br>**8.6Reflexos à luz**: 1.( )presente 2.( )ausente
**8.7 Fossas Nasais:**1.( )Permeáveis 2.( )Não permeáveis 3.( )Secreções, Características:
**8.8 Boca e Mucosa:** 1.( )Sujidade 2.( )SOG 3.( )Sangramento 4.( )Ressecamento 5.( )Sialorréia 6.( )Secreção 7.( )Sem anormalidades 8.( )Lesão,Qual:
**8.9 Dentição:**1.( )Presente 2.( )Ausente 3.( )
**8.10 Palato**: 1.( )Completo 2.( )Incompleto
**8.11Ouvido:** 1.( )Normal 2.( )Secreção 3.( )Deformidades 4.( )Baixa 5.( )Lesão,Qual?
**8.12Pescoço:** 1.( )Simétrico 2.( )Edema 3.( )Rigidez nuca 4.( )Assimétrico 5.( )Lesão, Qual?
**9. Sistema Nervoso**
**9.1 Fontanelas:**1.( )Normotensa 2.( )Tensa 3.( )Cheia 4.( )Deprimida 5.( )Abaulada 6.( )Fechada
**9.2 Suturas:**1.( )Normais 2.( )Separadas 3.( )Sobrepostas<br>**9.3 Tônus:**1.( )Normal 2.( ) Hipotônico 3.( )Flácido 4.( )Hipertônico
**9.4 Atividade Predominante**: 1.( )Alerta 2.( )Chorando 3.( )Sono Ativo 4.( )Sono Profundo
**9.5 Choro:** 1.( )Normal 2.( )Fraco 3.( )Outros:
**9.6 Resposta ao estímulo:** 1.( )Vocal 2.( )Toque 3.( )Dor 4.( )Nenhum
**9.7 Reflexos:**1.( )Babinski 2.( )Galant 3.( )Preensão 4.( )Moro 5.( )Busca 6.( )Sucção
**10. Sistema Respiratório**
**10.1 Respiração:**1.( )Regular 2.( )Irregular 3.( )Superficial 4.( )Apnéia 5.( )Bat. asas do nariz 6.( )Gemido expiratório 7.( )Taquipnéia 8.( )Bradipnéia
**10.2 Retração:** 1.( )Extrema 2.( )Intercostal 3.( )Subcostal
**10.3 Configuração do tórax**: 1.( )Simétrico 2.( )Assimétrico 3.( )Alteração Anatômica 4.( )Lesão, Qual?
**10.4 Mama:** 1.( )Simétrica 2.( )Assimétrica 3.( )Túrgida 4.( )Outros:
**10.5 Ausculta Pulmonar:** 1.( )Murmúrios vesiculares 2.( )Estertores 3.( )Roncos 4.( )Sibilos
**10.6 Secreção de Vias Aéreas:** ( )sim ( )não Aspecto:
**10.7 Ventilação:** 1.( )Ar ambiente 2.( )Hood 3.( )O2 circulante 4.( )VNI 5.( )CPAP nasal 6.( )CPAP endotraqueal 7.( )VMI, 8.PINSP:______9.PEEP:_____10.FiO2:_____11.Sat.02:______<br>12.Secreção COT/VAS: 1.( )não 2.( )sim,aspecto:
**11. Sistema Cardiovascular**
**11.1 Pulso Apical-Ritmo:** 1.( )Regular 2.( )Irregular
**11.2 Sopro:** 1.( )Ausente 2.( )Presente
**11.3 Perfusão Capilar:** 1.( )Normal - 1 a 3s 2.( )Lento – Maior que 3s
**11.4 Edema:** 1.( )Ausente 2.( )Pés 3.( )Mãos 4.( )Olhos 5.( )Anasarca
**11.5 Acesso Venoso:** 1.( )Umbilical 2.( )PICC 3.( )Periférico 4.( )Hidratado 5.( )Central
**12. Sistema Gastrointestinal**
**12.1 Abdome:** 1.( )Plano 2.( )Globoso 3.( )Tenso 4.( )Distendido 5.( )Timpânico 6.( )Flácido 7.RHA:7.1( )+ 7.2( )-
**12.2 Coto umbilical:** 1.( )Normal 2.( )Anormal 3.( )Secreções 4.( )Hiperemiado 5.( )Outros:
**12.3 Dieta:** 1.Ao seio ( )2.Oral/copinho 3.( )SOG 4.( )SNG 5.( )Zero<br>**12.4 RG**: 1.( )não 2.( )sim Aspecto: 1.( )Claro 2.( )Amarelado 3.( )Verde 4.( )Sanguinolento 5.( )Grumos 6.Volume(ml):
**12.5 Deglutição:** 1.( )Coordenada 2. ( )Insuficiente 3.( )Ausente 4.( )Não observado

**12.6 Evacuação:** 1.( )Espontânea 2.( )Estímulo 3.( )Ausente
**13. Sistema Geniturinário**
**13.1 Diurese:** 1.( )Espontânea 2.( )SVA 3.( )SVD 4.( )Oligúria 5.( )Anúria 6.( )Hematúria 7.( )Piúria
**13.2 Genitália:** 1.( )Normal 2.( )Edemaciada 3.( )Má formação congênita 4.( )Lesões,Qual:
**14. Binômio Mãe-filho**

14.1 Gravidez: 1.( )indesejada 2.( )não planejada	14.9 Estrutura mamária normal: 1.( )não 2.( )sim
14. 2Grande número de gestações:<br>1.( )não 2.( )sim	14.10 Gravidade da doença do RN:<br>1.()não 2.( )sim
14.3 Mãe/Pai não envolvido: 1.( )não 2.( )sim	14.11 Nível socioeconômico baixo:1.( )não 2.( )sim
14.4 Inexperiência com o cuidado ofertado:<br>1.( )não 2.( )sim	14.12 Conhecimento deficiente:<br>1.( )não 2.( )sim
14.5 Presença de estresse:1.( )não 2.( )sim	14.13 Depressão:1.( )não 2.( )sim
14.6 Funcionamento cognitivo limitado:<br>1.( )não 2.( )sim	14.14 Satisfação com amamentação(verbalizou):<br>1.( )não 2.( )sim
14.7 RN satisfeito após dieta: 1.( )não 2.( )sim	14.15 Esvaziamento da mama: 1.( )total 2.( )parcial
14.8 Conhecimento sobre amamentação:<br>1.( )não 2.()sim	14.16 Ordenha:1.( )não 2.( )sim

**15. Exames/Data/Resultados**	**Valores Encontrados (Exame mais recente)**	**Interpretação dos Resultados**<br>1.Normal 2. Alterado	**Data do Resultado**
Hemoglobina (Hg)	15.1	15.1.1	15.1.2
Hematócrito (Ht)	15.2	15.2.1	15.2.2
Plaquetas	15.3	15.3.1	15.3.2
Leucócitos	15.4	15.4.1	15.4.2
SHR	15.5	15.5.1	15.5.2
PCR	15.6	15.6.1	15.6.2
HMC/Culturas	15.7	15.7.1	15.7.2
Líquor	15.8	15.8.1	15.8.2
Gasometria	15.9	15.9.1	15.9.2
Radiografia	15.10	15.10.1	15.10.2
USG	15.11	15.11.1	15.11.2
Outros:	15.12	15.12.1	15.12.2

**16. Medicamentos em uso:**
ATB (esquema/data de início e término):
Drogas vasoativas:
Outros:

SUBCONJUNTO TERMINOLÓGICO DA CLASSIFICAÇÃO INTERNACIONAL PARA A PRÁTICA DE ENFERMAGEM (CIPE®) EM NEONATOS COM CATETER VENOSO CENTRAL DE INSERÇÃO PERIFÉRICA À LUZ DA TEORIA DE BETTY NEUMAN

MENSAGEM AOS LEITORES

O Subconjunto Terminológico da CIPE® para neonatos com Cateter Venoso Central de Inserção Periférica (PICC) à luz da Teoria de Betty Neuman foi estruturado com objetivo de subsidiar a prática de enfermagem a essa população específica na atenção neonatal hospitalar. Compuseram o Subconjunto 31 diagnósticos/resultados e 154 intervenções de enfermagem desenvolvidas com base na CIPE® versão 2019 e com conteúdo validados por especialistas do país.

Os diagnósticos/resultados e intervenções de enfermagem foram organizados utilizando o nível do estressor (intrapessoal, interpessoal e extrapessoal) e as intervenções seguiram as categorias de Primária, Secundária e Terciária.

Assim, 1 diagnóstico compôs o nível de estressor intrapessoal, 1 o nível interpessoal e 29 o nível extrapessoal. Quanto às intervenções de enfermagem, 64 contemplam as intervenções primárias, 56 as intervenções de nível secundário e 34 de nível terciário.

Nesse ínterim, recomenda-se a sua utilização pelos enfermeiros na assistência de enfermagem a recém-nascidos em uso do respectivo dispositivo na atenção hospitalar por meio das etapas do processo de enfermagem, através do raciocínio clínico e pensamento crítico. Destaca-se que durante o cuidado poderão ser identificados fenômenos de enfermagem não contemplados no subconjunto, o que não impossibilita o enfermeiro de inseri-lo em seu processo assistencial.

IMPORTÂNCIA PARA A ENFERMAGEM

A assistência de Enfermagem é definida por uma ferramenta de trabalho baseada em evidências científicas e que promove a eficácia das suas ações, através de resultados promissores na saúde do ser cuidado de uma maneira holística, e promove conforto, bem-estar e qualidade de vida (BAÓ et al., 2019), sendo essa prática embasada por um corpo de conhecimentos próprios da profissão.

Esse cuidado deve ser sistematizado, segundo o preconizado pela Resolução nº 358/2009 do Conselho Federal de Enfermagem (COFEN), que discorre acerca da Sistematização da Assistência de Enfermagem (SAE) e a utilização do Processo de Enfermagem (PE), método esse que orienta o cuidado profissional e o registro da sua prática, perpassa cinco etapas inter-relacionadas, interdependentes e recorrentes, a saber: Coleta de dados de enfermagem (ou Histórico de enfermagem); Diagnóstico de enfermagem; Planejamento de enfermagem; Implementação; e Avaliação de enfermagem (COFEN, 2009).

O PE deve ser orientado por uma teoria/modelo teórico de Enfermagem com base no contexto em que o profissional atua e no contexto de saúde onde o paciente está inserido. O presente subconjunto terá o suporte conceitual da Teoria de Betty Neuman, devido à sua pertinência e aplicabilidade à prática de Enfermagem nos mais diversos cenários de saúde.

Além de suporte teórico, é importante que a exequibilidade do PE seja viabilizada pela utilização de uma classificação dos termos que possam ser empregados na prática profissional no intuito de possibilitar uma linguagem única e padronizada, o que possibilitará uma linguagem universal (DOMINGOS et al., 2019).

No presente subconjunto terminológico, utilizou-se a Classificação Internacional para a Prática de Enfermagem (CIPE®), por se tratar de uma linguagem unificada da enfermagem que abrange diagnósticos/resultados e intervenções de Enfermagem como elementos inter-relacionados e interdependentes. Ressalta-se que a classificação é reconhecida como única terminologia internacional de enfermagem oficialmente reconhecida pela Organização Mundial da Saúde (OMS) (ICN, 2008).

A elaboração de subconjuntos é caracterizada como uma estratégia do International Council of Nurses (ICN) para viabilizar o uso dessa classificação durante a execução e registro do PE em dada situação do cuidado em saúde (ICN, 2008; NÓBREGA; GARCIA, 2009; LINS et al., 2013).

O avanço tecnológico no cuidado neonatal contribuiu para a sobrevida de recém-nascidos gravemente enfermos (LIU; CHEN; WANG, 2015). Como parte do processo assistencial intensivo e especializado, a Unidade de Terapia Intensiva Neonatal é um ambiente onde são efetivados procedimentos essenciais ao tratamento do neonato hospitalizado (PACHECO et al., 2020). Nesse contexto, a

prática assistencial demanda a realização de procedimentos invasivos, entre eles a inserção de cateteres venosos (DUESING; FAWLEY; WAGNER, 2016).

Entre os diversos tipos de cateteres vasculares utilizados em neonatologia, cabe destaque ao Cateter Venoso Central de Inserção Periférica (PICC). Seu uso é amplamente apoiado por reduzir as complicações associadas aos cateteres centrais convencionalmente usados. Por permitir que a terapêutica medicamentosa seja realizada, o dispositivo pode contribuir para salvar a vida de neonatos hospitalizados (YU et al., 2018).

Nesse contexto, o cuidado recém-nascidos com necessidade de terapia intravenosa em uso do PICC exige habilidades e conhecimentos específicos do enfermeiro. O procedimento de inserção do cateter supracitado consiste em prática avançada e de alta complexidade. O enfermeiro com competência técnica e legal capacitado é responsável pela inserção, fixação, manutenção e remoção do cateter. O respaldo ético e legal para realização do procedimento no País encontra-se amparado pela Resolução COFEN nº 258/2001, atualizada pelo Parecer COFEN N°243/2017 (COFEN, 2001; COFEN, 2017).

Apesar da segurança na administração prolongada de soluções intravenosas, o PICC não está isento de riscos e eventos adversos, assim, os principais problemas que podem surgir com o uso do cateter são a infecção ou bacteremia a ele associada, além de complicações de caráter mecânico, como obstrução, extravasamento, ruptura do cateter e remoção não eletiva (PADILLA-SÁNCHEZ et al., 2019).

Nesse ínterim, o manejo do cateter PICC pressupõe habilidades e competências que favoreçam a segurança do recém-nascido durante todo o tratamento e que reduzam os riscos de complicações associadas ao cateter.

INSERÇÃO DA TEORIA DE BETTY NEUMAN NO CUIDADO AO NEONATO COM CATETER VENOSO CENTRAL DE INSERÇÃO PERIFÉRICA

Para o desenvolvimento do presente subconjunto terminológico recorreu-se a Teoria de Betty Neuman com vistas a subsidiar a construção do subconjunto com seus diagnósticos/resultados e intervenções de Enfermagem, além de propiciar uma linguagem própria à Enfermagem a partir de um corpo de conhecimentos próprio advindo das teorias/modelos teóricos de Enfermagem tornando-o, assim, singular à

profissão, além de ser uma recomendação do International Council of Nurses (ICN, 2008),

Aplicável a nível mundial, o Modelo de Sistemas de Neuman, baseado na teoria geral do sistema, é ancorado no relacionamento contínuo do cliente com os fatores de estresse ambientais, os quais têm potencial para causar uma reação ao estresse (NEUMAN, 1995). O Modelo é composto por componentes principais, o estressor e a reação a esse estressor. O indivíduo, família ou grupo de pessoas é visto como um sistema aberto que busca o equilíbrio entre as variáveis estressantes do meio ambiente (NEUMAN, 1995).

Segundo Neuman, os estressores são classificados como intrapessoal (forças ambientais internas que ocorrem dentro dos limites do sistema do cliente), interpessoal (forças externas de interação ambiental que ocorrem fora dos limites do sistema do cliente na faixa proximal) e extrapessoal (forças externas de interação ambiental que ocorrem fora dos limites do sistema do cliente na faixa distal).

A partir do reconhecimento do estressores, são elencados os níveis de proteção a serem alcançados: Prevenção primária: está relacionada à identificação e redução de fatores de risco para prevenir possíveis reações; Prevenção secundária: está relacionada à sintomatologia após uma reação a estressores, classificação apropriada de prioridades de intervenção e tratamento para reduzir seus efeitos nocivos; e Prevenção terciária: está relacionada aos processos de ajuste que ocorrem quando a reconstituição começa e os fatores de manutenção movem o cliente de volta de uma maneira circular em direção à prevenção primária.

As situações estressantes acarretadas pelo Cateter Venoso Central de Inserção Periférica podem levar ao desequilíbrio do sistema, logo se torna importante o conhecimento das barreiras protetoras deste sistema com a intenção de fortalecê-las na busca da estabilidade da saúde do recém-nascido.

Ao entender o contexto do neonato em uso do cateter epicutâneo à luz da Teoria de Betty Neuman, o enfermeiro reconhecerá as necessidades desses pacientes através da identificação dos fatores estressores a que os mesmos estão expostos, como também as potencialidades promovidas pelas linhas de defesa do sistema, com o intuito de possibilitar o desenvolvimento dos diagnósticos de enfermagem e das intervenções necessárias para o enfrentamento satisfatório dos estressores e para a elaboração de um planejamento com vistas ao cuidado integral.

ENUNCIADOS DIAGNÓSTICOS/RESULTADOS E INTERVENÇÕES DE ENFERMAGEM SEGUNDO BETTY NEUMAN

Nível do estressor	Diagnóstico/Resultado de enfermagem	Intervenções de Enfermagem
Intrapessoal	Risco de Hemorragia/Controle de Risco de Hemorragia	Identificar Risco de Hemorragia; Monitorar Débito de Líquidos; Monitorar Equilíbrio de Líquidos (ou Balanço Hídrico); Gerenciar Sangramento; Medir (ou verificar) Débito de Líquidos.
Interpessoal	Agitação/Agitação, Reduzida	Avaliar Sinais de Agitação; Obter Dados sobre Ambiente; Avaliar Presença de Ruído; Obter Dados sobre Nível de Estresse; Monitorar Dor; Posicionar Paciente; Monitorar Sinais Vitais; Diminuir Barulho; Manipular conforme Necessidade de Cuidados;
Extrapessoal	Dor/Controle da Dor	Implementar Guia de Conduta para Dor; Monitorar Dor; Posicionar Paciente; Administrar Medicação para Dor Analgesia Controlada por Enfermeira(o); Avaliar Resposta à Medicação; Avaliar Resposta ao Manejo (Controle) da Dor.
Extrapessoal	Risco de Infecção/Controle de Risco de Infecção	Cuidados com Local de Dispositivo Invasivo; Monitorar Sinais e Sintomas de Infecção; Utilizar Técnica Asséptica; Prevenir Infecção; Obter Dados sobre Sinais e Sintomas de Infecção.
Extrapessoal	Infecção/Infecção, Ausente	Cuidados com Local de Dispositivo Invasivo; Monitorar Sinais e Sintomas de Infecção; Utilizar Técnica Asséptica; Prevenir Infecção; Obter Dados sobre Sinais e Sintomas de Infecção.
Extrapessoal	Hipotermia/Controle da Hipotermia	Promover Termorregulação, Positiva; Obter Dados sobre Risco de Termorregulação, Negativa; Monitorar Temp. Corporal; Avaliar Resposta à Termorregulação.
Extrapessoal	Termorregulação, Prejudicada/Termorregulação, Eficaz	Promover Termorregulação, Positiva; Obter Dados sobre Risco de Termorregulação, Negativa; Monitorar Temp. Corporal Avaliar Resposta à Termorregulação.
Extrapessoal	Bradicardia/Bradicardia, Ausente	Medir (ou Verificar) Frequência Cardíaca; Monitorar Condição Cardíaca; Monitorar Condição Fisiológica; Obter Dados sobre Condição Cardíaca

Extrapessoal	Edema/Edema, Ausente	Cuidados com Local de Dispositivo Invasivo; Implementar Regime de Cuidados com a Pele; Manter Integridade da Pele; Monitorar Equilíbrio de Líquidos (ou Balanço Hídrico); Monitorar Perfusão Tissular; Medir (ou Verificar) Débito de Líquidos.
Extrapessoal	Hematoma/Hematoma, Ausente	Cuidados com Local de Dispositivo Invasivo; Implementar Regime de Cuidados com a Pele; Manter Integridade da Pele; Aplicar Compressa Fria.
Extrapessoal	Integridade da Pele, Prejudicada/Integridade da Pele, Eficaz	Cuidados com Local de Dispositivo Invasivo; Implementar Regime de Cuidados com a Pele; Manter Integridade da Pele.
Extrapessoal	Trauma por Dispositivo Invasivo/Resposta ao Trauma por Dispositivo, Melhorada	Regime de Cuidados com o Dispositivo Invasivo; Executar Medida de Segurança; Prevenir Lesão Mecânica.
Extrapessoal	Integridade Tissular, Prejudicada/ Integridade Tissular, Eficaz	Manter Integridade da Pele; Monitorar a Integridade da Pele; Obter Dados sobre Integridade da Pele; Regime de Cuidados com a Pele; Tratar Condição da Pele
Extrapessoal	Perfusão Tissular Periférica, Prejudicada/ Perfusão Tissular Periférica, Eficaz	Avaliar Perfusão Tissular; Monitorar Perfusão Tissular; Obter Dados sobre Perfusão Tissular, Periférica
Extrapessoal	Risco de Embolia/Controle do Risco de Embolia	Avaliar Presença de Ar em Dispositivo para Infusão; Gerenciar Sintomas de Embolia; Monitorar Saturação de Oxigênio Sanguíneo Usando Oxímetro de Pulso; Prevenir Embolia; Manipular Dispositivo Invasivo com Atenção; Evitar Terapia de Infusão em Dispositivo Invasivo com Resistência; Avaliar Retirada de Dispositivo Invasivo.
Extrapessoal	Risco de Necrose	Avaliar Condição do Dispositivo Invasivo; Tratar Condição da Pele; Implementar Regime de Cuidados com a Pele; Obter Dados sobre Risco de Perfusão Tissular, Ineficaz.
Extrapessoal	Função do Sistema Regulatório, Prejudicada/ Função do Sistema Regulatório, Eficaz	Medir (ou Verificar) Dispositivo Invasivo; Requisitar (ou Requerer) Exame de Serviço de Radiologia; Avaliar Localização de Dispositivo Invasivo pelo Exame; Estabilizar Dispositivo Invasivo com Curativo; Prevenir Trombose Venosa;

Extrapessoal	Sinal Vital (Sinais Vitais), Alterado/Sinal Vital, no limite normal	Identificar condição fisiológica; Monitorar condição fisiológica; Monitorar Sinais Vitais.
Extrapessoal	Risco de Função do Sistema Nervoso, Prejudicada/ Controle do Risco de Função do Sistema Nervoso	Medir (ou Verificar) Dispositivo Invasivo; Requisitar (ou Requerer) Exame de Serviço de Radiologia; Avaliar Localização de Dispositivo Invasivo pelo Exame; Monitorar Sinais Vitais
Extrapessoal	Risco de Função do Sistema Respiratório, Prejudicada/ Controle do Risco de Função do Sistema Respiratório	Medir (ou Verificar) Dispositivo Invasivo; Requisitar (ou Requerer) Exame de Serviço de Radiologia; Avaliar Localização de Dispositivo Invasivo pelo Exame; Estabilizar Dispositivo Invasivo com Curativo; Monitorar Sinais Vitais; Monitorar Saturação de Oxigênio Sanguíneo Usando Oxímetro de Pulso; Avaliar Sintomas de Dispnéia; Avaliar Ritmo Cardíaco; Prevenir Choque. Auscultar o Pulmão; Monitorar Saturação de Oxigênio Sanguíneo Usando Oxímetro de Pulso; Monitorar Sinais Vitais
Extrapessoal	Risco de Perfusão Tissular, Ineficaz/ Controle do Risco de Perfusão Tissular	Monitorar Sinais Vitais; Monitorar Saturação de Oxigênio Sanguíneo Usando Oxímetro de Pulso; Avaliar Sintomas de Dispnéia; Avaliar Ritmo Cardíaco; Prevenir Choque.
Extrapessoal	Risco de Condição Neurológica, Prejudicada/ Controle do Risco de Condição Neurológica	Medir (ou Verificar) Dispositivo Invasivo; Requisitar (ou Requerer) Exame de Serviço de Radiologia; Avaliar Localização de Dispositivo Invasivo pelo Exame; Monitorar Sinais Vitais
Extrapessoal	Risco de Função Cardíaca, Prejudicada/ Controle do Risco de Função Cardíaca	Medir (ou Verificar) Dispositivo Invasivo; Requisitar (ou Requerer) Exame de Serviço de Radiologia; Avaliar Localização de Dispositivo Invasivo pelo Exame; Estabilizar Dispositivo Invasivo com Curativo; Monitorar Sinais Vitais; Monitorar Saturação de Oxigênio Sanguíneo Usando Oxímetro de Pulso; Avaliar Sintomas de Dispnéia; Avaliar Ritmo Cardíaco; Prevenir Choque.
Extrapessoal	Ritmo Cardíaco, Prejudicado/ Ritmo Cardíaco, Eficaz	Medir (ou Verificar) Dispositivo Invasivo; Requisitar (ou Requerer) Exame de Serviço de Radiologia; Avaliar Localização de Dispositivo Invasivo pelo Exame; Estabilizar

		Dispositivo Invasivo com Curativo; Monitorar Sinais Vitais; Monitorar Saturação de Oxigênio Sanguíneo Usando Oxímetro de Pulso; Avaliar Sintomas de Dispnéia; Avaliar Ritmo Cardíaco; Prevenir Choque.
Extrapessoal	Alergia a Látex/ Alergia a Látex, Controlada	Identificar Alergia no Paciente; Evitar Contato ao Látex; Monitorar a Integridade da Pele.
Extrapessoal	Alergia/ Alergia, Ausente	Identificar Alergia no Paciente; Identificar Alergia no Leito do Paciente;
Extrapessoal	Risco de Alergia a Medicação/ Controle do Risco de Alergia a Medicação	Confirmar (ou Comprovar) Alergia; Avaliar Lista de Medicação; Avaliar Necessidade de Exame Laboratorial; Comunicar ao Médico; Avaliar Necessidade de Teste Diagnóstico.
Extrapessoal	Complicação Associada à Atenção à Saúde/ Complicação Associada à Atenção à Saúde, Ausente	Executar Medida de Segurança; Promover Higiene das Mãos; Utilizar Técnica Asséptica; Avaliar Curativo; Implementar Regime de Cuidados com Dispositivo Invasivo; Monitorar Sinais e Sintomas de Infecção.
Extrapessoal	Obstrução do Cateter/ Obstrução do Cateter, Ausente	Avaliar Dispositivo Invasivo; Lavar Dispositivo Invasivo com Solução Isotônica; Implementar Regime para Lavar Dispositivo Invasivo antes e depois das Medicações;
Extrapessoal	Risco de Medicação Adversa/ Risco de Medicação Adversa, Ausente	Implementar Regime de Segurança; Gerenciar o Risco de Efeito Colateral da Medicação; Obter Dados sobre Risco de Interação Medicamentosa, Adversa.
Extrapessoal	Interação Medicamentosa, Adversa/ Interação Medicamentosa Adversa, Ausente	Avaliar Lista de Medicação; Consultar Farmaceutico sobre Lista de Medicação; Consultar Prestador (ou Provedor) de Cuidados de Saúde sobre Efeitos Colaterais da Medicação; Obter Dados sobre Efeitos Colaterais da Medicação; Alterar o Horário da Medicação conforme Orientação Multiprofissional.

TUTORIAL DE UTILIZAÇÃO DO SUBCONJUNTO TERMINOLÓGICO

O subconjunto apresentado deverá ser utilizado na prática assistencial de enfermagem, segundo as etapas do Processo de Enfermagem: Diagnóstico de enfermagem, identificando um dos 31 diagnósticos de enfermagem presentes no subconjunto; Planejamento da assistência, organizando os diagnósticos de enfermagem previamente identificados em ordem prioritária, a partir das necessidades apresentadas pelo recém-nascido em uso do PICC. Ainda, devem ser identificadas as intervenções de enfermagem com o intuito de resolver os diagnósticos de enfermagem; Na implementação, onde são postos em prática as intervenções planejadas anteriormente, com base nas prioridades do neonato; Avaliação, onde serão avaliados se os diagnósticos foram resolvidos, melhorados, piorados ou apresentam-se inalterados. Logo, para a determinação dos resultados de enfermagem, utiliza-se o foco do diagnóstico modificando ou acrescentando apenas um julgamento a este.
